# Condition dependence of (un)predictability in escape behavior of a grasshopper species

**DOI:** 10.1093/beheco/arad047

**Published:** 2023-06-13

**Authors:** Gabe Winter, Luis Wirsching, Holger Schielzeth

**Affiliations:** Population Ecology Group, Institute of Ecology and Evolution, Friedrich Schiller University Jena, Dornburger Straße 159, 07743 Jena, Germany; Population Ecology Group, Institute of Ecology and Evolution, Friedrich Schiller University Jena, Dornburger Straße 159, 07743 Jena, Germany; Population Ecology Group, Institute of Ecology and Evolution, Friedrich Schiller University Jena, Dornburger Straße 159, 07743 Jena, Germany

**Keywords:** condition dependence of animal behavior, double-hierarchical generalized linear model, escape behavior, Orthoptera, residual intra-individual variance, unpredictability

## Abstract

(Un)predictability has only recently been recognized as an important dimension of animal behavior. Currently, we neither know if (un)predictability encompasses one or multiple traits nor how (un)predictability is dependent on individual conditions. Knowledge about condition dependence, in particular, could inform us about whether predictability or unpredictability is costly in a specific context. Here, we study the condition dependence of (un)predictability in the escape behavior of the steppe grasshopper *Chorthippus dorsatus*. Predator–prey interactions represent a behavioral context in which we expect unpredictability to be particularly beneficial. By exposing grasshoppers to an immune challenge, we explore if individuals in poor condition become more or less predictable. We quantified three aspects of escape behavior (flight initiation distance, jump distance, and jump angle) in a standardized setup and analyzed the data using a multivariate double-hierarchical generalized linear model. The immune challenge did not affect (un)predictability in flight initiation distance and jump angle, but decreased unpredictability in jump distances, suggesting that unpredictability can be costly. Variance decomposition shows that 3–7% of the total phenotypic variance was explained by individual differences in (un)predictability. Covariation between traits was found both among averages and among unpredictabilities for one of the three trait pairs. The latter might suggest an (un)predictability syndrome, but the lack of (un)predictability correlation in the third trait suggests modularity. Our results indicated condition dependence of (un)predictability in grasshopper escape behavior in one of the traits, and illustrate the value of mean and residual variance decomposition for analyzing animal behavior.

## Teaser

Choosing a good strategy for escaping predators is essential for the survival of prey species. Having unpredictable escape paths seem to be the preferred strategy among insects, but not much is known about what causes such behavior. We find that aspects of the escape behavior of grasshoppers in poor health conditions decreased in unpredictability. This suggests that unpredictability (sometimes) does not occur by mistake but rather represents a costly and desirable feature.

## INTRODUCTION

Animal behavior varies both between individuals and within individuals. While variation in individual average behavioral responses has been intensely studied under the label of animal personality (Dall et al. 2012; Sih et al. 2015), the (un)predictability component is still not fully recognized as a dimension of animal behavior. Psychologists (Fiske and Rice 1955; Bem and Allen 1974; Ram et al. 2005; Siegler 2007; Wang et al. 2012) and neuroscientists (Hedden and Gabrieli 2004; MacDonald et al. 2006; Tamnes et al. 2012) have decades-long traditions of studying (un)predictability in humans, while its importance has only recently been acknowledged in behavioral and evolutionary ecology (Réale et al. 2010; Stamps et al. 2012; Westneat et al. 2015; Martin et al. 2017). Within the last few years, evidence for individual-level variation in (un)predictability of animal behavior has nonetheless been accumulating (Mitchell et al. 2021).

The still widespread neglect of individual differences in (un)predictability incurs costs of lost opportunities. Meta-analyses show that in animal behavior studies, only about 40% of the behavioral variability is accounted for by differences in average behavior among individuals (Bell et al. 2009), meaning that most of the behavioral variability is left unexplored when the intra-individual levels of variance are ignored. Exploring this residual intra-individual variance (rIIV, here called “(un)predictability” when referring to the predictability-unpredictability axis, and “unpredictability” when referring to high variability in behavior), thus offers opportunities for a better understanding of animal behavior (Westneat et al. 2015). In fact, (un)predictability might represent an integral part of a behavioral syndrome involving plasticity (Briffa et al. 2013; O’Dea et al. 2019; Maskrey et al. 2021) and animal personality (i.e. among individual variance, or average behavioral expression, Highcock and Carter 2014; Mitchell et al. 2016; He et al. 2017; Jolles et al. 2019; Niemela et al. 2019; Hertel et al. 2021), and may even be seen as an animal personality axis itself. We here refer to (un)predictability as an axis of individual variation with some individuals being unpredictable and others predictable. What we do not know (yet) is whether (un)predictability refers to a single general dimension of animal behavior (independent of any specific trait being considered) or if it is better treated as a trait-specific aspect of behavioral variability (e.g. unpredictability in boldness vs. unpredictability in activity).

Unpredictability in behavior can arise for multiple reasons. One reason is that microenvironmental conditions differ causing different optimal trait values in repeated observations. This can lead to differences in (un)predictability if environments experienced by individuals differ, even if plasticity itself is identical across individuals. While this might be a common occurrence under natural conditions, it will apply less to highly controlled experimental settings. However, current evidence does not suggest systematic differences in (un)predictability between field and lab studies (Mitchell et al. 2021). This suggests that (un)predictability could vary according to state variables of individuals (MacDonald et al. 2006; Horvath et al. 2019a; but see Cornwell et al. 2023). Furthermore, individuals can differ in how sensitive they are in perceiving environmental cues (Briffa 2013), and in the accuracy with which they assess the environment (“organismal error,” Westneat et al. 2015). Indeed, brain structure, neurotransmission, and neuronal activity have been linked to behavioral unpredictability (MacDonald et al. 2006). Likewise, metabolic capabilities may also affect an individual’s (un)predictability (Velasque and Briffa 2016; Mitchell and Biro 2017; Biro et al. 2018), and variation in energy reserves has been found to affect unpredictability in a context-specific manner (Horvath et al. 2017, 2019b). Finally, apparent unpredictability may also arise from measurement error on the side of the observer (“observer error,” Westneat et al. 2015), although measurement error alone is not expected to produce individual differences in (un)predictability. Still, the potential for measurement error, state differences, and microenvironmental differences emphasize the need for a well-controlled experimental setup for quantifying (un)predictability.

A few studies have shown that (un)predictability has a heritable basis and can thus evolve by natural selection (Martin et al. 2017; Henriksen et al. 2020; Prentice et al. 2020). Unpredictability is likely favored in some contexts, while consistency (including phenotypic canalization) is more beneficial in others. In some bird species, for example, songs seem to be selected for particularly stereotyped forms with higher reproductive success in individuals with consistent performance (Byers 2007; Botero et al. 2009; Węgrzyn et al. 2010). In other species, however, song variability is selected for (Robinson and Creanza 2019). In fish, individuals that are predictable in risk-taking behavior perform better as group leaders and are more successful foragers (Ioannou and Dall 2016) and individuals with increased predictability in aggression are more attractive as mating partners (Scherer et al. 2018). In spiders, males that are consistent in aggression are more likely to win contests against conspecifics (Kwek et al. 2021). And a more complex relationship was found in deer, where individuals with either the highest or the lowest levels of (un)predictability in fighting have lower mating success than individuals with intermediate (un)predictability (Jennings et al. 2013).

A context in which unpredictability seems particularly advantageous is the interaction between predators and prey (Mitchell et al. 2021). In cases of repeated interactions, it can benefit both the predator and the prey to show variability in behavior and exploit the moment of surprise. If unpredictability represents a neurobiological challenge, then a predator–prey interaction might partly become a competition in the ability to change behavior and unpredictability can be under open-ended selection. Indeed, crustaceans, for example, behave more unpredictably in risk-taking behavior when exposed to predator cues (Briffa 2013) or when in an unfamiliar (potentially riskier) environment (Horvath et al. 2019a). Among insects, erratic (unpredictable) escape paths are common and have been predicted to confuse predators (Humphries and Driver 1970). In fact, a study using humans as model predators in a simulation showed that unpredictable prey are significantly more difficult to catch (Jones et al. 2011), potentially increasing the survival of prey with highly variable escape paths.

It has been hardly explored if being predictable or unpredictable in risky or competitive situations is actually challenging. To our knowledge, only one study looked at the condition dependence of unpredictability (Horvath et al. 2019a). We here use a predator escape context to study if (un)predictability is affected by experimental manipulation of individual condition. We do so in a species of grasshopper, which allows us to repeatedly quantify response behavior to simulated predator attacks. We assume that in a predator–prey interaction context, it is likely beneficial to be unpredictable and that at least some predator attacks happen in pursuit and thus involve repeated interactions. We exposed steppe grasshoppers (*Chorthippus dorsatus*) to an immune challenge that impairs individual condition. If being unpredictable poses a challenge, we predict impaired individuals to become more predictable (stereotyped) in their responses. However, if organismal error makes it challenging to perform in a stereotyped way, we predict control individuals to be more predictable. The manipulation thus allows us to assess if (un)predictability is condition-dependent and if unpredictability is a potentially desirable feature or a sign of failure in performance.

## MATERIALS AND METHODS

In order to test the condition dependence of (un)predictability, we exposed steppe grasshoppers *C. dorsatus* to an immune challenge (*n* = 66) and recorded the effect of the treatment on three escape behavior traits, in comparison to untreated individuals (*n* = 66).

### Subjects and housing

Individuals were sampled as nymphs in June/July 2021 from Jena, Germany (50°56ʹ41.2″N 11°36ʹ37.8″E). Subjects were transferred to the laboratory, separated by sex, and maintained in groups of up to about 60 individuals in large mesh cages of dimensions 47.5 × 47.5 × 93 cm^3^. After their final molt, individuals were transferred in pairs of the same sex and the same final molt date to smaller mesh cages of dimensions 22 × 16 × 16 cm^3^. Individuals were maintained with ad libitum access to freshly cut grass provided in small vials with water and a water tube for moisture. Dead individuals and individuals that lost legs were replaced, if possible, with other individuals that matched the cage mate. When a replacement was impossible, the remaining cage mate was not used in the experiment.

Around 15 days after the final molt, a behavioral assay was performed as described below. To achieve standardized conditions across the behavioral essay, individuals were placed in refrigerators at 8 °C on the evening prior to phenotyping. During overnight cooling, individuals were kept individually in cylindrical vials of dimensions 8 cm height and 5 cm diameter, with 2–3 leaves of grass. For each pair of the same sex and final molt date, one individual was randomly selected for an immune challenge, while the other was used as a control.

### Immune challenge

To cause an impairment in the condition of individuals, we performed an immune challenge. Treated individuals were anesthetized by exposing them for 3 min to temperatures of −20 °C. They were then injected with a solution containing 1.6 × 10^8^ cells of heat-killed *Escherichia coli* per microliter diluted in Ringer’s grasshopper solution. The cell concentration was determined after the efficacy of five different solutions (with 1.6 × 10^8^, 1.6 × 10^7^, 1.6 × 10^6^, 1.6 × 10^5,^ and 1.6 × 10^4^ cells/μL) was tested in 20 individuals each (10 males and 10 females). The chosen concentration caused the death of 25% of the individuals after 48 h and thus seemed sufficiently strong without being immediately fatal. Males received 1 µL of the solution, and females, 2 µL (females weigh around twice as much as males). In order to maximize the differences between control and treated groups (and because we were not interested in the effect of heat-killed *E. coli* as such), control individuals did not receive any injection nor were they anesthetized. After injections, all individuals (treatment and control) were color-marked dorsally with yellow paint (for automated analysis, see below). In order to keep track of individual identity, one of the individuals was marked with a stripe from the wings to the pronotum, and the other with a stripe on the wings and a dot on the pronotum. The type of marking was randomly assigned and thus not associated with the treatment groups. After being marked, individuals were left for 1 h under a heat lamp in mesh cages of dimensions 22 × 16 × 16 cm³, with fresh grass available, as preparation for the behavioral assay.

### Escape behavior phenotyping

Each pair of treated and control individuals was phenotyped simultaneously, with the observer being blind to the treatment. The two individuals were placed into a 5.3 × 3.3 m² arena, where they could acclimatize for 5 min. The arena floor was coated with foam rubber to provide a non-slippery surface for the grasshoppers to jump. The room temperature was maintained constant at 27 °C. Individuals were approached with a moveable device (the “chaser,” see Supplement 1 for details), which was placed on the ground about 50 cm behind the grasshopper and moved smoothly and at a constant speed towards it (approximately 130 cm/s^2^), mimicking an approaching predator. Each individual had ten escape jumps recorded on video, with 2 min time gap between chases. To streamline phenotyping, we alternately simulated attacks on the two individuals in the arena. On average, 14 individuals were phenotyped per phenotyping date. The software Ethovision XT (Noldus, Netherlands), with the Social Interaction Module, was used to track the movements of the chaser and the grasshopper. Effectively, the software tracks the yellow dorsal color applied to all individuals and the dark brown color that marked the chaser. Flight initiation distance (FID), jump distance, and jump angle measurements were extracted from the tracks. FID was defined as the minimum distance (in cm) between the chaser and the grasshopper before the individual jumped. Jump distance was defined as the distance (in cm) between the point where the jump started and where the grasshopper landed. Finally, jump angle was defined as the angle of the jump direction (in degrees) in relation to the initial grasshopper body position. For jump angle, straight jumps scored 0°, while negative values marked jumps to the left, and positive values jumps to the right. When the grasshopper did not move after the simulated attack, the observation was excluded from the analysis.

### Modeling

We used two modeling approaches. In the first two-step approach, we modeled the individual average behavior and the (un)predictability (as standard deviations and coefficients of variation) in separate univariate generalized linear mixed models (LMMs) for each trait. The second approach was done with a multivariate double-hierarchical generalized linear model (DHGLM). While we think the DHGLM model represents the more appropriate analysis, the two-step approach serves as a reference that is closer to traditional analyses. All models were fitted using the R package *brms* version 2.18.0 (Bürkner 2017) with default priors. For both approaches, we ran two MCMC chains for an adaptive phase of 4500 iterations before taking posterior samples every 30 iterations for a total of 30,000 iterations. This produced 2000 samples from the posterior distributions.

### Two-step approach

We used the individual average trait values of flight initiation distance, jump distance, and jump angle in LMMs with sex and treatment fitted as fixed effects, centered to a meaningful zero (Schielzeth 2010), and pair identity as a random effect. For treatment, the control group was set as a reference, and treatment effects were estimated as contrasts. Sex was centered to zero by coding females as −0.5 and males as 0.5, such that effects are estimated as contrasts between males vs. females. Pair ID refers to the pair of a treated and a control individual that was phenotyped simultaneously. Both individuals of a pair belonged to the same home cage and had the same sex and age.

For the (un)predictability, we used the standard deviations of each individual, with the same fixed and random variables used in the average trait models. For FID and jump distance, we additionally modeled the coefficient of variation, as the individual standard deviation and average behavior were correlated in these traits (*r* = 0.67, *P* < 0.001 and *r* = 0.37, *P* < 0.001, respectively).

### Multivariate DHGLM

We fitted a multivariate DHGLM with FID (log-transformed), jump distance, and jump angle as three response variables. DHGLMs consist of a mean and a dispersion part and thus allow modeling hierarchical variation in average behavior and unpredictability within a single modeling framework (Cleasby et al. 2015). Both mean and dispersion parts of the model were fitted with the following fixed effects: treatment (immune-challenged or control), sex, daytime (time of the day converted to decimals), and jump order (sequential number of jumps within the 10 trials). We also fitted an interaction between treatment and sex. Unpredictability was modeled as logarithms of standard deviations.

All fixed effects were centered to a meaningful zero. Treatment and sex were centered as described in the two-step approach. Daytime and jump order were centered on the average value in our data. Phenotyping date (the date when the experiment was conducted) and individual ID were used as random effects in the mean and the dispersion parts of the model with unstructured variance–covariance matrices for estimating covariation among traits and between averages and (un)predictability. At the residual level, we estimated the covariance between trait means because covariances involving means and variances are undefined at the level of residuals. Fixed and random effects were defined a priori based on their biological importance. To assure that variables would not be selected for their significance concerning the condition treatment, we randomized the treatment vector to adjust the model before running the model with the real treatment vector (see Supplement 2 for code details).

### Model fit checks

We assessed convergence using the Gelman-Rubin convergence criterion (all $\hat{R}$ ≤ 1.004) and by inspection of trace plots. The effective sample size was ≥ 1528.4 for all parameters. Model fit was also checked with posterior predictive checks. We used default priors that are non-informative or very weakly informative and should have negligible influence on the posterior distributions (Bürkner 2017). Uninformativeness was checked by comparing prior to posterior distributions, illustrating that the posterior was dominated by the data rather than the priors. Posterior distributions are summarized by their median, standard error, and 95% highest posterior density (HPD). Plots and code for all model fit checks are presented in Supplement 2.

### Model estimates

Posterior samples of fixed effect slopes were standardized for the standard deviation of each covariate (by multiplying estimates by the standard deviation in the covariate) and used to make inferences about the parameters in the model. We considered fixed effects (and covariances among random components) to have a statistically significant effect when its 95% HPD interval did not include zero.

Variance decomposition was done based on posterior distributions from the DHGLM using equations presented in O’Dea et al. (2021) and described in detail in Supplement 2. Differences in repeatabilities among traits were tested with pairwise 2 sample *t*-tests (Supplementary Table S2 and Supplementary Figure S7). Supplement 2 contains the commented code for all analyses. Code and data are also available in Winter et al. (2023a). All analyses were performed in R 4.1.3 (R Core Team 2022).

## RESULTS

We recorded the escape behavior of 132 steppe grasshopper individuals (70 females, 62 males). To test whether unpredictability is condition-dependent, half of the individuals were impaired by an immune challenge (*n* = 66). In 17% of the trials, individuals did not jump after the simulated attack, and this behavior was more frequent in impaired individuals (20.5% vs. 13.3%, χ^2^ = 10.686, df = 1, *P* = 0.001). The following analyses include only trials in which individuals jumped.

**Figure 1.**
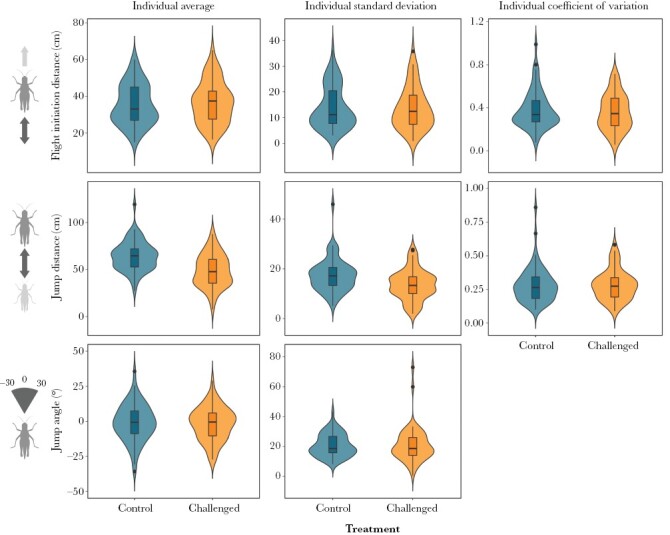
Observed measurements of escape behavior traits for immune-challenged and untreated steppe grasshopper *Chorthippus dorsatus*. Plots represent average (on the left plots), standard deviation (on the center plots), and coefficient of variation (on the right plots) values per individual, based on up to 10 observations per individual.

Flight initiation distance was on average 36.5 ± 18.3 cm for the control group and 37.7 ± 18.8 cm for treated individuals. The average jump distance was 63.8 ± 22.6 cm for the control group and 51.2 ± 21.7 cm for treated individuals. And the average jump angle was −1.2 ± 24.5° and −1.5 ± 24.7° for the control and treatment groups, respectively. The observed individual average as analyzed in the two-step approach (Figure 1), suggest that impaired grasshoppers jumped shorter distances than the ones in good condition (β = −15.44 ± 2.81 [−21.36: −10.30], posterior median ± SE [95% HPD interval]). The treatment also seemed to reduce individual standard deviation in jump distance (β = −3.68 ± 1.12 [−5.94: −1.59]), but not its coefficient of variation (β = 0.00 ± 0.02 [−0.04: 0.03]). In contrast, there was no indication of a difference between treatment groups in either individual average, individual standard deviation, and coefficient of variation for FID (Mean: β = 0.97 ± 1.42 [−1.86: 3.67]; Standard deviation: β = −0.47 ± 1.28 [−2.96: 2.01]; Coefficient of variation: β = −0.02 ± 0.03 [−0.08: 0.07]), or for jump angle (Mean: β = −0.62 ± 2.16 [−5.01: 3.52]; Standard deviation: β = 0.06 ± 1.59 [−3.04: 3.32]).

**Figure 2.**
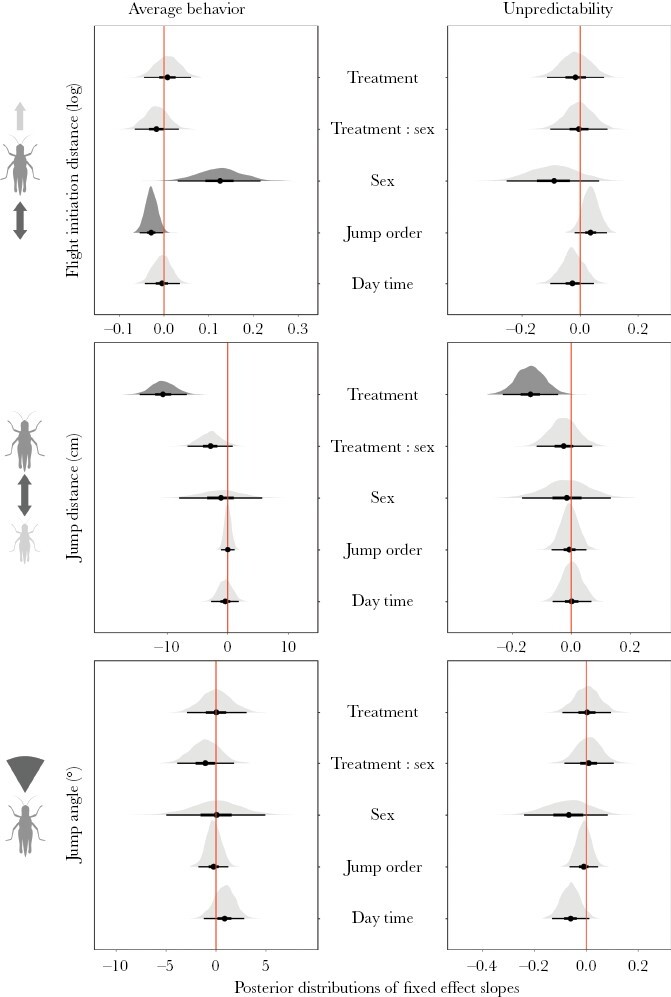
Posterior distributions of fixed effect slopes for escape behavior traits of the steppe grasshopper *C. dorsatus.* Slopes were standardized for the standard deviation of each covariate. Points show the median and thick and thin bars represent 50% and 95% HPD intervals, respectively. The darker shade of gray highlights cases in which 95% HPD intervals do not include zero.

The DHGLM confirmed the negative effect of treatment on the average and unpredictability values of jump distance (Supplementary Table S1 and Figure 2). The model also confirmed the absence of treatment effect on FID and jump angle. Besides the treatment, FID average values were mainly influenced by sex, with males jumping earlier than females when attacked. In trials performed later in a phenotyping session (a higher value for jump order), FID was decreased. No significant predictor was found for the average jump angle.

**Figure 3.**
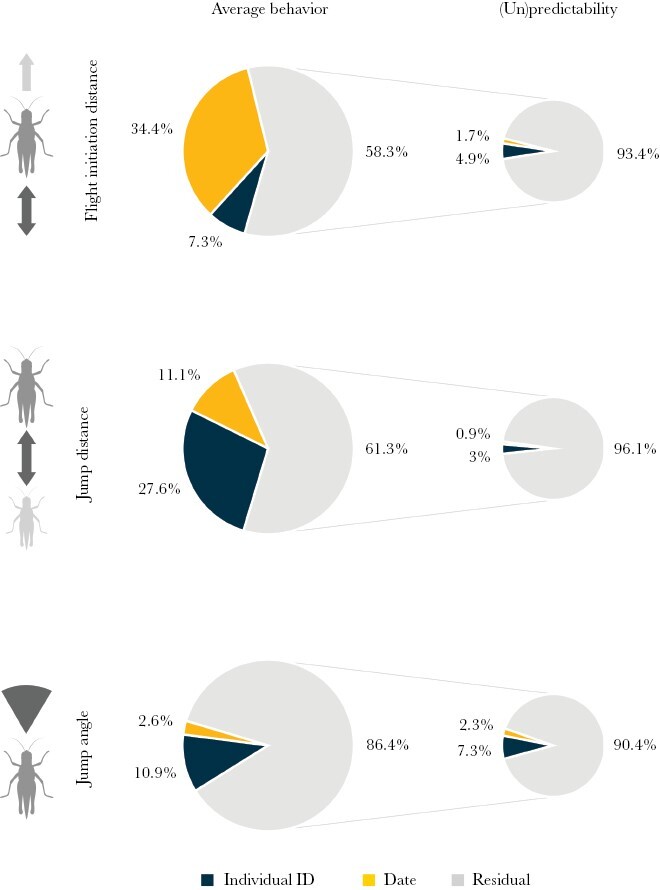
Variance decomposition of escape behavior in the steppe grasshopper *C. dorsatus*. The residual variance of average behavior (gray slice on the left pies) is further decomposed into the amount of variance explained by phenotyping date, individual identity, and residual variance in unpredictability (right pies). Variance components were estimated while accounting for fixed effects.

After controlling for fixed effects, around 28% of the variance in jump distance was explained by average differences among individuals (Figure 3). For FID and jump angle, in contrast, inter-individual differences in average behavior explained only a small part of the phenotypic variance (7.3% and 10..9%). For all traits, around 60–85% of the total variance was unexplained by the mean model. Some of the residual variances were explained by inter-individual differences in (un)predictability (4.9% for FID, 3% for jump distance, and 7.3% for jump angle), but it was mostly left unexplained as residual variance (93.4% for FID, 96.1% for jump distance, and 90.4% for jump angle). Our data also shows that patterns of variances differ between traits (Supplementary Table S2 and Supplementary Figure S7) and likely also between contexts and species. Note that the analysis was based on a single session per individual and therefore does not allow conclusions about the long-term stability of trait values.

**Figure 4.**
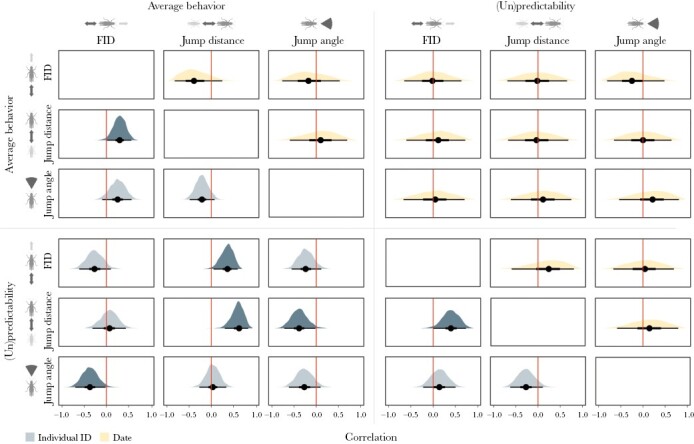
Correlations among traits in the steppe grasshopper *C. dorsatus*. Plots show the posterior distributions of correlations among traits in the mean and dispersion parts of a multivariate DHGLM at the level of individuals (lower triangle) and phenotyping dates (upper triangle). The darker shades highlight cases in which 95% HPD intervals do not include zero.

The multivariate DHGLM also allowed us to assess covariation among traits and between the mean and the dispersion parts of the model (Supplementary Table S3 and Figure 4). At the level of individuals, correlations among average trait values (average posterior mean correlation: 0.11) were significantly positive for FID and jump distance. Correlations among (un)predictabilities (average: 0.09) were also significantly positive for (un)predictability in FID and jump distance. Correlations between averages and (un)predictabilities within traits (average: 0.02) were significantly positive for jump distance only. Correlations between averages and (un)predictabilities among traits were very variable (average: −0.09) with 1) a significantly positive correlation between average jump distance and (un)predictability in FID, 2) a significantly negative correlation between average FID and (un)predictability in jump angle, and 3) a significantly negative correlation between average jump angle and (un)predictability in jump distance. In contrast to the individual level, HPD intervals for correlations at the level of phenotyping date were all broadly overlapping zero (Figure 4).

## DISCUSSION

We aimed to assess if unpredictability in escape behavior is a desirable feature or a performance error by manipulating the individual condition of steppe grasshoppers with an immune challenge. The treatment had a negative effect on individual average and (un)predictability of jump distance. Impaired individuals jumped on average shorter distances and were more consistent. This, along with the increased number of non-jumps in treated individuals and death rates in the pretest, illustrates that the treatment affected escape performance negatively. However, there was no effect of the treatment on (un)predictability in FID and jump angle. Variance decomposition shows that jump distance was primarily explained by inter-individual differences in both average behavior and (un)predictability, while FID and jump angle were mostly explained by inter-individual differences in (un)predictability alone. Correlations between averages and unpredictabilities were significantly positive for one trait (jump distance) and there was a significantly positive covariation between one out of three unpredictability pairs. Covariation among some components of behavior and independent variation of others suggest some modularity in escape behavior.

Animal personality studies have long used models that assume residual variance to be uniformly distributed, thus not differing among individuals (Cleasby and Nakagawa 2011; Westneat et al. 2015). However, we know that a big portion of behavioral variation remains in such residual variation of classical models (Bell et al. 2009). DHGLMs have recently been introduced as a most efficient way to explore behavioral variation including the differences in (un)predictability (Cleasby et al. 2015; O’Dea et al. 2021). Using this method, we found that the proportion of phenotypic variance explained by inter-individual differences in average behavior and (un)predictability differ among traits. Jump distance was mostly explained by inter-individual differences in average behavior. The distance a grasshopper can jump might mostly be determined by morphological features, such as hindleg size and body weight, and energy availability, which vary among individuals. For FID and jump angle, we assume that trait expression depends more on situation-specific decisions rather than on morphological constraints. Only a small portion of the total phenotypic variance in FID and jump angle was explained by inter-individual differences in average values and was rather defined by differences in (un)predictability. Differences in components of variation, therefore, apparently depend on how the traits are expressed mechanistically.

Covariation among (un)predictabilities, and among trait averages and (un)predictabilities can be informative about behavioral syndrome structure. The relationship between average behavior and (un)predictability has been described for several taxa and behavioral contexts (Highcock and Carter 2014; Mitchell et al. 2016; He et al. 2017; Jolles et al. 2019; Niemela et al. 2019; Hertel et al. 2021), although they vary in direction. We here report a significantly positive correlation between average jump distance and (un)predictability in jump distance, suggesting that long-jumpers were also more variable. It might not be too surprising that, given the capability of long jumps, it would be easier to vary in jump distances compared to when jump distances are more limited. Note, however, that there might be a change in (un)predictabilities as a consequence of changes in the mean as we discuss below. Covariation in (un)predictability was significantly positive among FID and jump distance so individuals with variable FID also tended to show variable jump distances. This might indicate some trait-generality of (un)predictability. (Un)predictability in the third trait, however, was uncorrelated, such that there seems to be some modularity in (un)predictability. There are currently so meager data on correlations among (un)predictabilities that it could be premature to draw general conclusions from those associations. More data and different model systems are clearly needed.

Unpredictability could arise simply from performance errors, including erroneous assessment of the environment (Westneat et al. 2015). We aimed to assess this possibility by impairing individual condition with an immune challenge. If unpredictability occurred only by mistake and failure to produce a canalized response, the impaired individuals would have an increased level of unpredictability. On the other hand, if unpredictability is costly in any way—perceptionally, cognitively, or energetically—we expect impaired individuals to be more consistent. We found a negative effect of treatment in unpredictability in jump distance, but not for FID and jump angle, suggesting that unpredictability might be costly for some traits but not for others. In another study on condition dependence of (un)predictability in risk-taking behavior, pill bugs infected with *Wolbachia* took less risk than healthy individuals but did not change their levels of (un)predictability (Horvath et al. 2019a). Whether this pattern of limited or context-specific condition effects on unpredictability is generally valid remains to be explored in more species and contexts.

The energetic or cognitive costs of unpredictability remain poorly explored. Some studies relate high unpredictability to higher energetic levels (Velasque and Briffa 2016; Biro et al. 2018; Horvath et al. 2019b). We found that unpredictability in jump distance decreased in impaired individuals. Our data rather suggest costs of unpredictability than costs of predictability. Unpredictability may therefore be costly in some way that depends on the current condition, even though it is difficult to pinpoint the costs based on the current state of evidence. Since we do not think that variability in the actual trait expression is energetically costly, we rather suspect that it requires efficient cognitive performance to generate variability. This might depend on structures established during development (expressed in individual differences) as well as current capabilities (expressed in condition dependencies), to perform unpredictable behavior.

We controlled for additional biologically relevant variables that could potentially affect average escape behavior and (un)predictability. We found that males showed higher FID, jumping earlier than females, which might be interpreted as reduced boldness. This could be related to lower costs of escape jumps in lighter males as compared to significantly heavier females. In trials recorded later in the phenotyping session (which consisted of 10 chases), grasshoppers allowed the chaser to get closer before jumping, which indicates some habituation to the stimulus. Besides these statistically significant effects, we found trends that—although not statistically significant—can be instructive for the most likely direction of potential effects. Males tended to respond more strongly to the treatment, jumping even shorter distances than females when immune-challenged. The effect may, therefore, depend on sex or correlates thereof. In the dispersion part of the model, there was a trend for males to be more consistent than females in both FID and jump angle. Females might compensate for increased boldness (if this is reflected in reduced FID) by increased unpredictability (Stamps et al. 2012).

It might be biological or coincidental that increased unpredictability co-occurs with increased trait expression (as, for example, in the sex effect on jump distance). However, the dependency may also be a statistical by-product of increased variability as trait values increase. It is conceivable that traits like jump distance (and maybe also FID) have fixed boundaries and that a reduction of unpredictability is simply a consequence of a reduction of the upper boundary (with consequential reduction of mean and variance). Cause and effect remain unknown since we cannot distinguish if individuals aim to reduce their mean trait values or variability in trait expression. Changes in unpredictability (reductions, in particular) may possibly be caused by changes in mean trait values. This interpretation might apply to our manipulations of condition that reduced the mean as well as the unpredictability in jump distance, but also to the sex difference with reduced distances and variability in males. The analyses of coefficients of variation suggest that changes in unpredictability are not explained by changes in the mean alone. Besides dependencies of unpredictabilities on the mean, differences in predictability in our experimental setup could have resulted from individual-specific reaction norms in response to habituation to repeated chases.

Our results support the emerging trend that individual differences in behavior should not be summarized by individual average values, but that (un)predictability should be explicitly considered. Appropriate statistical methods have already been developed to explore the hierarchical nature of individual differences in behavior. Our data suggest both costs of unpredictability, at least in some traits, and some modularity in (un)predictability as a trait. These important questions certainly deserve further study. Our data also shows that patterns of variances differ between traits and likely also between contexts and species. Our results rather suggest that unpredictability does not arise (solely) from organismal errors. Further studies are needed to identify the costs and challenges that make individuals unpredictable in contexts where unpredictability appears beneficial.

## Supplementary Material

arad047_suppl_Supplementary_Material_1

arad047_suppl_Supplementary_Material_2

## Data Availability

Analyses reported in this article can be reproduced using the data provided by Winter et al. (2023b).
