# Supplementary material for: Condition dependence of (un)predictability in escape behavior of a grasshopper species
Source: Behav Ecol. 2023 Jun 13;34(5):741–50. doi: 10.1093/beheco/arad047 (PMC10516674; doi:10.1093/beheco/arad047)
Supplement: arad047_suppl_Supplementary_Material_1 [file arad047_suppl_supplementary_material_1.pdf]

Condition-dependence of (un)predictability in escape behavior of a grasshopper species

Supplement 1: Additional methods and results

Phenotyping protocol

Individuals were taken from their home cages on the evening before phenotyping. They were placed in cylindrical vials of dimensions 8 cm height and 5 cm diameter containing 2-3 leaves of grass and their individual and pair identities were recorded on their vials. Vials of paired individuals with the same home cage, age, and sex were kept together at all times. The vials were then placed inside a tray and put in the fridge at 8ºC.

Phenotyping happened the following morning. Every 30 minutes a randomly chosen pair was taken from the fridge. One randomly chosen individual of the pair was anesthetized and injected with a solution as described in the Materials and Methods section. Both individuals were then marked with a yellow paint marker (Edding 750). One randomly chosen individual of the pair was marked with an "i" on the dorsal side of the wings and pronotum, while the other individual was marked with an "I" (Figure S1). Since the type of marking was randomly assigned independent of the treatment, the observer was blind regarding the condition treatment of individuals, while individuals could be easily distinguished.

Both grasshoppers were placed in individual cages under a heating lamp for 1h, with water and food available. The pair was then taken to the arena to acclimatize. One individual was placed in the center of the left side of the arena, while the other individual was placed in the center of the right side. The temperature in the arena was maintained at approximately 27ºC, using air conditioning and heat lamps placed above the arena. Trials started after an acclimatization period of 5 min.

A camera on a tripod with wheels (Figure S2) was slowly moved and placed on top of the grasshopper on the left side of the arena. The "chaser" (Figure S3) was then placed on the floor behind the grasshopper at approximately 40-50 cm of distance. Using a smartphone to remotely control the software Ethovision on the computer, the video started to be recorded and a 2 minutes timer was set. The chaser was then moved toward the grasshopper until it jumped. After the jump, the recording was interrupted and the camera was slowly moved on top of the grasshopper on the right side of the arena, where the same procedure was repeated. After the timer of the first individual ended, the second trial of that individual was started. The same procedure was followed alternatively until both individuals had 10 chases recorded.

When a grasshopper jumped away from the arena, it was gently captured with a vial and placed in the original spot in the center of the left or right side of the arena. This happened in about 15% of the cases. After the 10 trials of each individual were completed, the same protocol was followed for the second pair of grasshoppers, until all pairs from the fridge had been processed.

Figure S1: Grasshopper marked with the yellow marker.

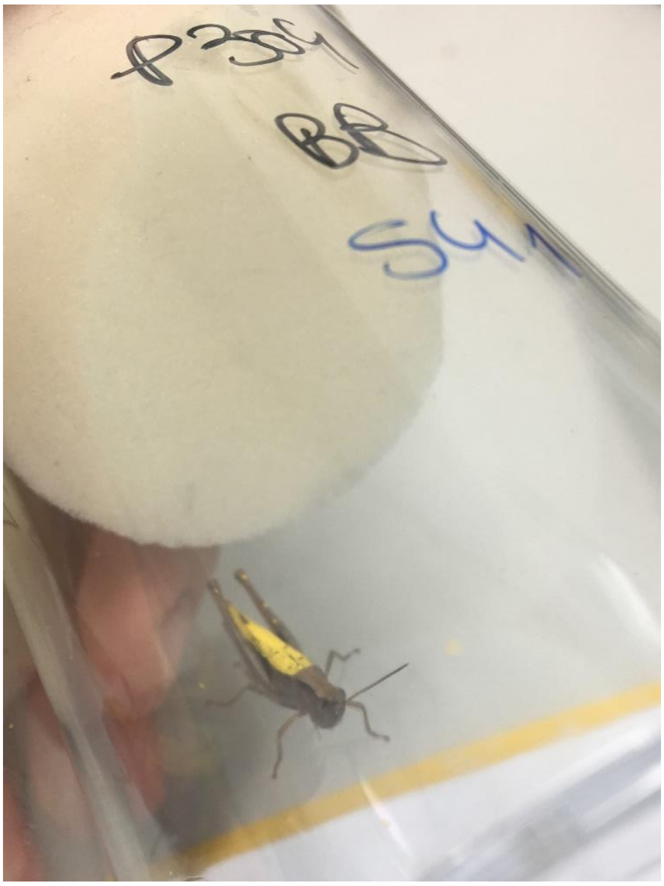

Figure S2: Tripod and camera setup.

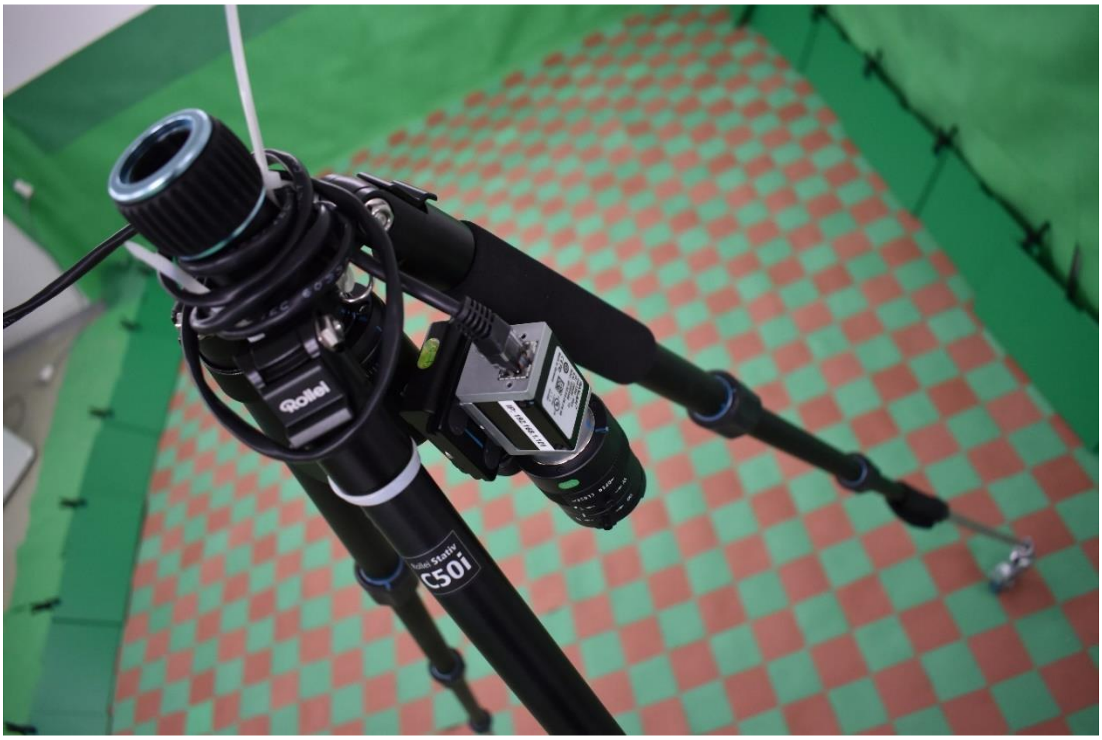

Figure S3: Chaser used to trigger escape jumps.

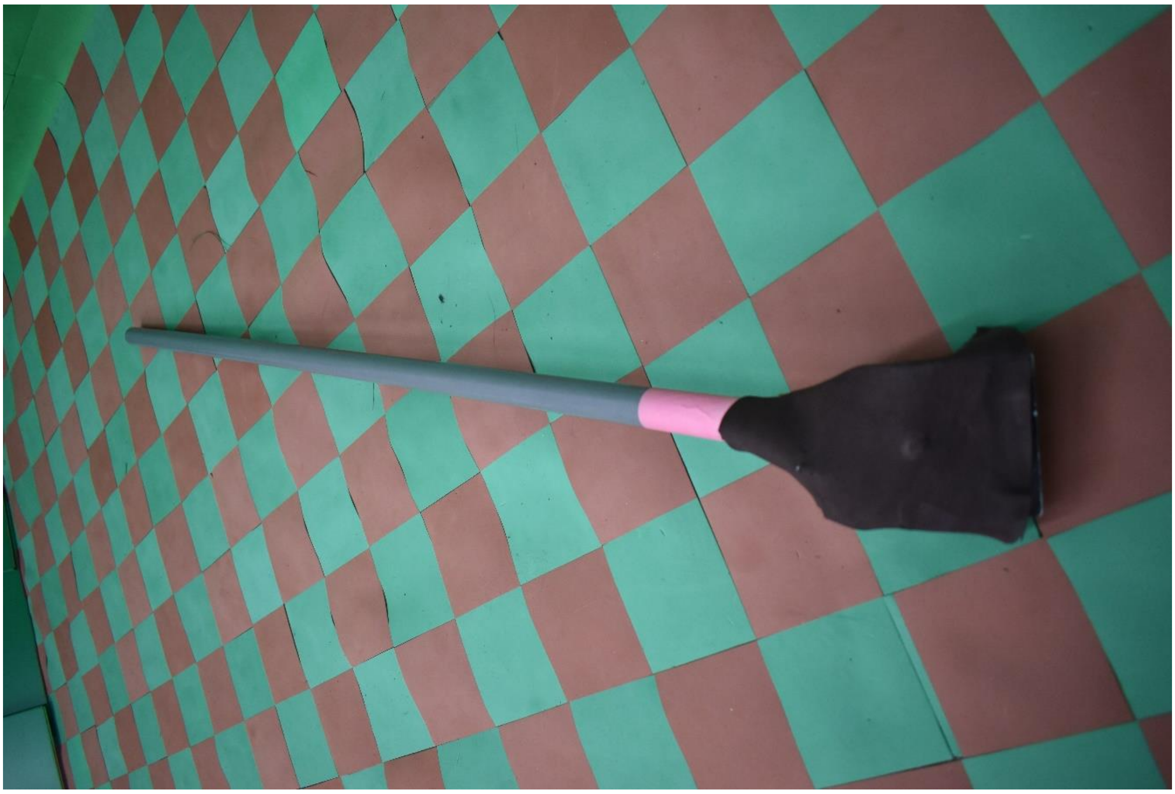

Figure S4: Camera point of view.

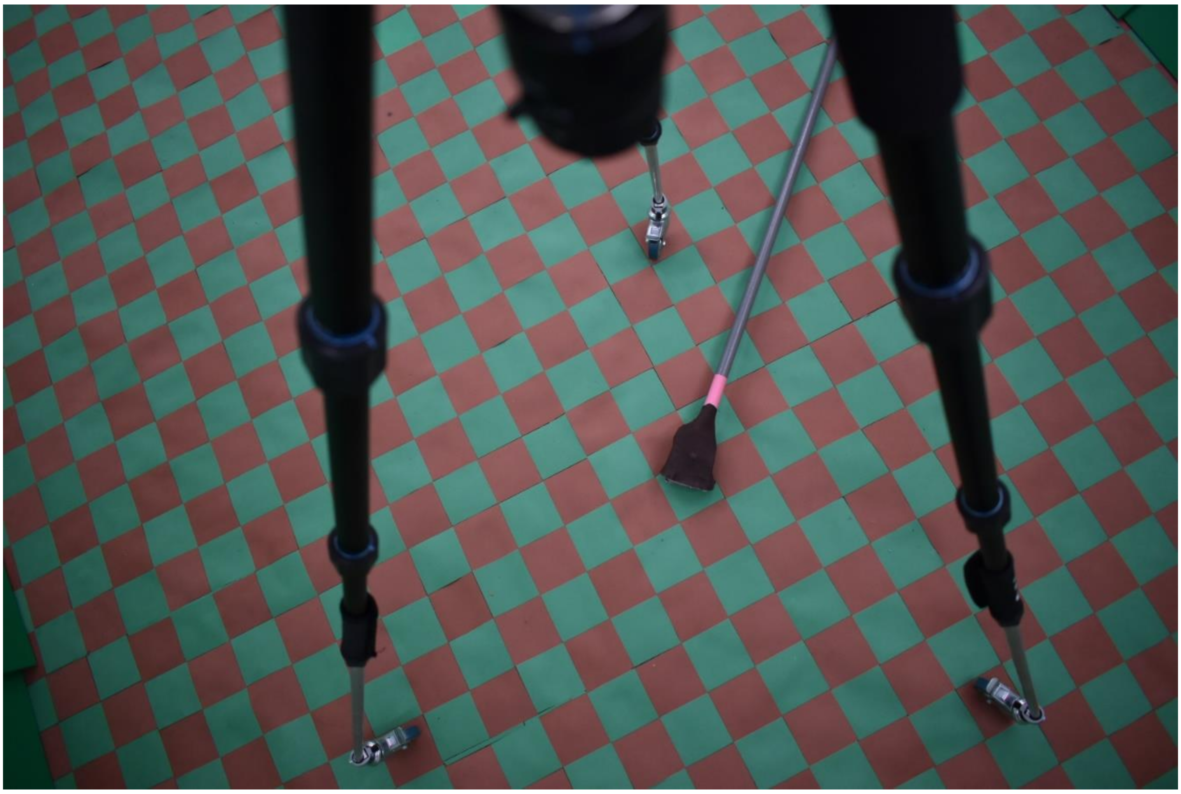

Figure S5: Experimenter point of view.

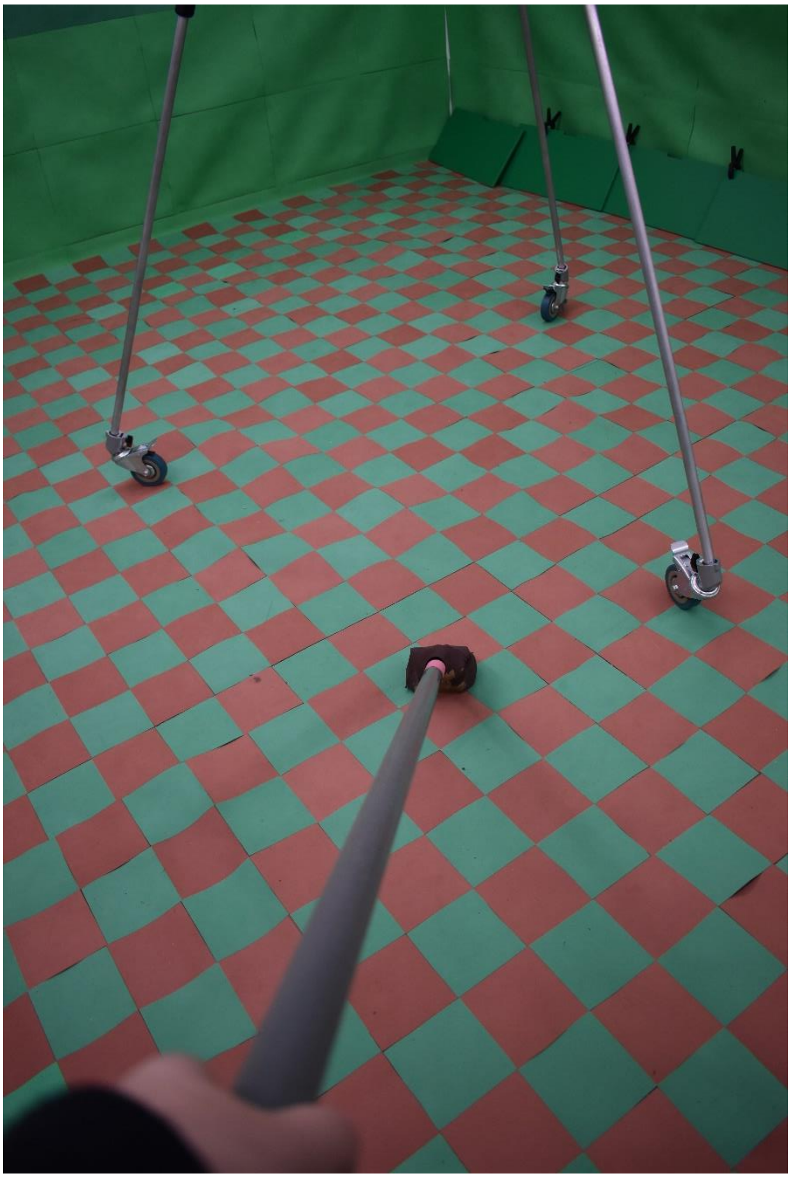

Figure S6: Example of chaser and grasshopper tracks in the software Ethovision.

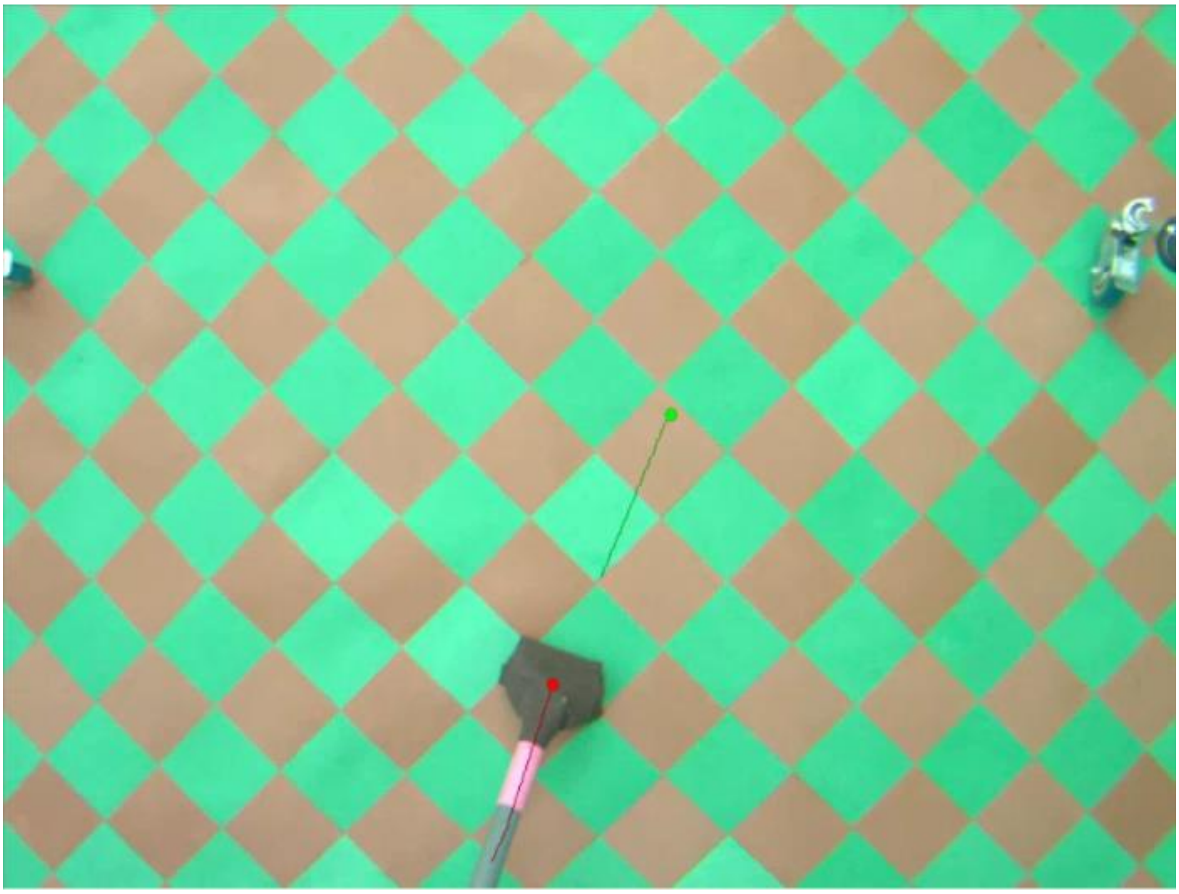

Table S1: Model estimates based on a multivariate double-hierarchical generalized linear model applied to three aspects of escape behavior in steppe grasshoppers *Chorthippus dorsatus*.

**Flight initiation distance**						
	Average behavior			Unpredictability		
Fixed effects	Median estimate	Est. Error	95% HPD interval	Median estimate	Est. Error	95% HPD interval
Intercept	3.5241	0.1207	[3.2891 : 3.7700]	-0.9631	0.0761	[-1.1051 : -0.8077]
Treatment	0.0109	0.0380	[-0.0661 : 0.0820]	-0.0227	0.0713	[-0.1619 : 0.1174]
Sex	0.1774	0.0682	[0.0445 : 0.3059]	-0.1269	0.1172	[-0.3605 : 0.0901]
Daytime	-0.0027	0.0117	[-0.0236 : 0.0214]	-0.0155	0.0223	[-0.0581 : 0.0293]
Jump order	-0.0095	0.0044	[-0.0182 : -0.0012]	0.0119	0.0093	[-0.0055 : 0.0311]
Treatment : Sex	-0.0495	0.0748	[-0.1950 : 0.0888]	-0.0123	0.1424	[-0.2841 : 0.2792]
Random effects	R^2		95% HPD interval	R^2		95% HPD interval
Individual ID	0.0700		[0.0279 : 0.1209]	0.0470		[0.0115 : 0.0940]
Phenotyping Date	0.3287		[0.1301 : 0.6132]	0.0092		[0.0000 : 0.0571]
Residual	0.5972		[0.3491 : 0.7964]	0.9383		[0.8690 : 0.9832]
	CV_{ID}		95% HPD interval	CV_{ID}		95% HPD interval
Coefficient of individual variation	0.0437		[0.0330 : 0.0577]	0.6241		[0.4216 : 0.8438]
**Jump distance**						
	Average behavior			Unpredictability		
Fixed effects	Median estimate	Est. Error	95% HPD interval	Median estimate	Est. Error	95% HPD interval
Intercept	63.6241	3.6431	[55.8055 : 70.1339]	2.8520	0.0678	[2.7228 : 2.9969]
Treatment	-15.1139	2.7912	[-20.2912 : -9.3886]	-0.1957	0.0675	[-0.3247 : -0.0621]
Sex	-1.5894	4.8381	[-11.7247 : 7.4029]	-0.0215	0.1079	[-0.2396 : 0.1828]
Daytime	-0.2360	0.6829	[-1.6259 : 1.0241]	0.0008	0.0197	[-0.0380 : 0.0383]
Jump order	0.0005	0.1859	[-0.3312 : 0.3926]	-0.0022	0.0100	[-0.0218 : 0.0169]
Treatment : Sex	-8.1850	5.4654	[-18.5668 : 2.8259]	-0.0739	0.1384	[-0.3252 : 0.2147]
Random effects	R^2		95% HPD interval	R^2		95% HPD interval
Individual ID	0.2767		[0.1822 : 0.3748]	0.0285		[0.0083 : 0.0548]
Phenotyping Date	0.0923		[0.0000 : 0.2632]	0.0044		[0.0000 : 0.0316]
Residual	0.6182		[0.4786 : 0.7283]	0.9645		[0.9264 : 0.9908]
	CV_{ID}		95% HPD interval	CV_{ID}		95% HPD interval
Coefficient of individual variation	0.2189		[0.1754 : 0.2608]	0.5106		[0.3345 : 0.7345]
**Jump angle**						
	Average behavior			Unpredictability		
Fixed effects	Median estimate	Est. Error	95% HPD interval	Median estimate	Est. Error	95% HPD interval
Intercept	-1.6621	2.0746	[-5.5017 : 2.7293]	3.0638	0.0686	[2.9336 : 3.2047]
Treatment	0.0335	2.1284	[-4.1088 : 4.2167]	0.0047	0.0687	[-0.1278 : 0.1365]
Sex	0.0763	3.4993	[-6.5866 : 7.3184]	-0.0946	0.1195	[-0.3329 : 0.1200]
Daytime	0.5059	0.5991	[-0.6940 : 1.6503]	-0.0351	0.0211	[-0.0744 : 0.0085]

Jump order	-0.0817	0.2470	[-0.5015 : 0.4652]	-0.0031	0.0092	[-0.0215 : 0.0144]
Treatment : Sex	-3.1045	4.1996	[-11.4984 : 4.7648]	0.0276	0.1395	[-0.2405 : 0.3040]
Random effects	R^2		95% HPD interval	R^2		95% HPD interval
Individual ID	0.1066		[0.0508 : 0.1724]	0.0719		[0.0345 : 0.1150]
Phenotyping Date	0.0169		[0.0000 : 0.0831]	0.0142		[0.0000 : 0.0759]
Residual	0.8692		[0.7786 : 0.9332]	0.9070		[0.8495 : 0.9541]
	CV_{ID}		95% HPD interval	CV_{ID}		95% HPD interval
Coefficient of individual variation	-3.1206		[-43.3806 : 45.0626]	0.5453		[0.3501 : 0.7336]

Table S2: Pairwise two-sample t-test for trait repeatabilities.

			t	df	Difference 95% CI
**Average behavior**					
Individual ID					
Jump distance	x	Flight initiation distance	164.9	2983.5	[0.2010 : 0.2059]
Jump angle	x	Flight initiation distance	40.42	3834.3	[0.0344 : 0.0379]
Jump angle	x	Jump distance	-128.79	3378.8	[-0.1699 : -0.1648]
Phenotyping date					
Jump distance	x	Flight initiation distance	-68.74	3270.7	[-0.2399 : -0.2265]
Jump angle	x	Flight initiation distance	-106.38	2210.7	[-0.3235 : -0.3118]
Jump angle	x	Jump distance	-45.21	2577.4	[-0.0881 : -0.0808]
Residual					
Jump distance	x	Flight initiation distance	9.852	3099.5	[0.0238 : 0.03564]
Jump angle	x	Flight initiation distance	100.76	2452.3	[0.2760 : 0.2869]
Jump angle	x	Jump distance	147.72	3334.1	[0.2484 : 0.2551]
**Unpredictability**					
			t	df	Difference 95% CI
Individual ID					
Jump distance	x	Flight initiation distance	-33.07	3162.2	[-0.0202 : -0.0179]
Jump angle	x	Flight initiation distance	35.07	3976.2	[0.0227 : 0.0254]
Jump angle	x	Jump distance	78.94	3306	[0.0420 : 0.0442]
Phenotyping date					
Jump distance	x	Flight initiation distance	-12.97	3251.9	[-0.0087 : -0.0064]
Jump angle	x	Flight initiation distance	8.298	3841	[0.0050 : 0.0081]
Jump angle	x	Jump distance	20.69	2885.2	[0.0128 : 0.0154]
Residual					
Jump distance	x	Flight initiation distance	31.83	3248.6	[0.0249 : 0.0282]
Jump angle	x	Flight initiation distance	-31.62	3953.5	[-0.0325 : -0.0287]
Jump angle	x	Jump distance	-73.85	3460	[-0.0587 : -0.0557]

Figure S7: Distribution of the repeatabilities in the random effects.

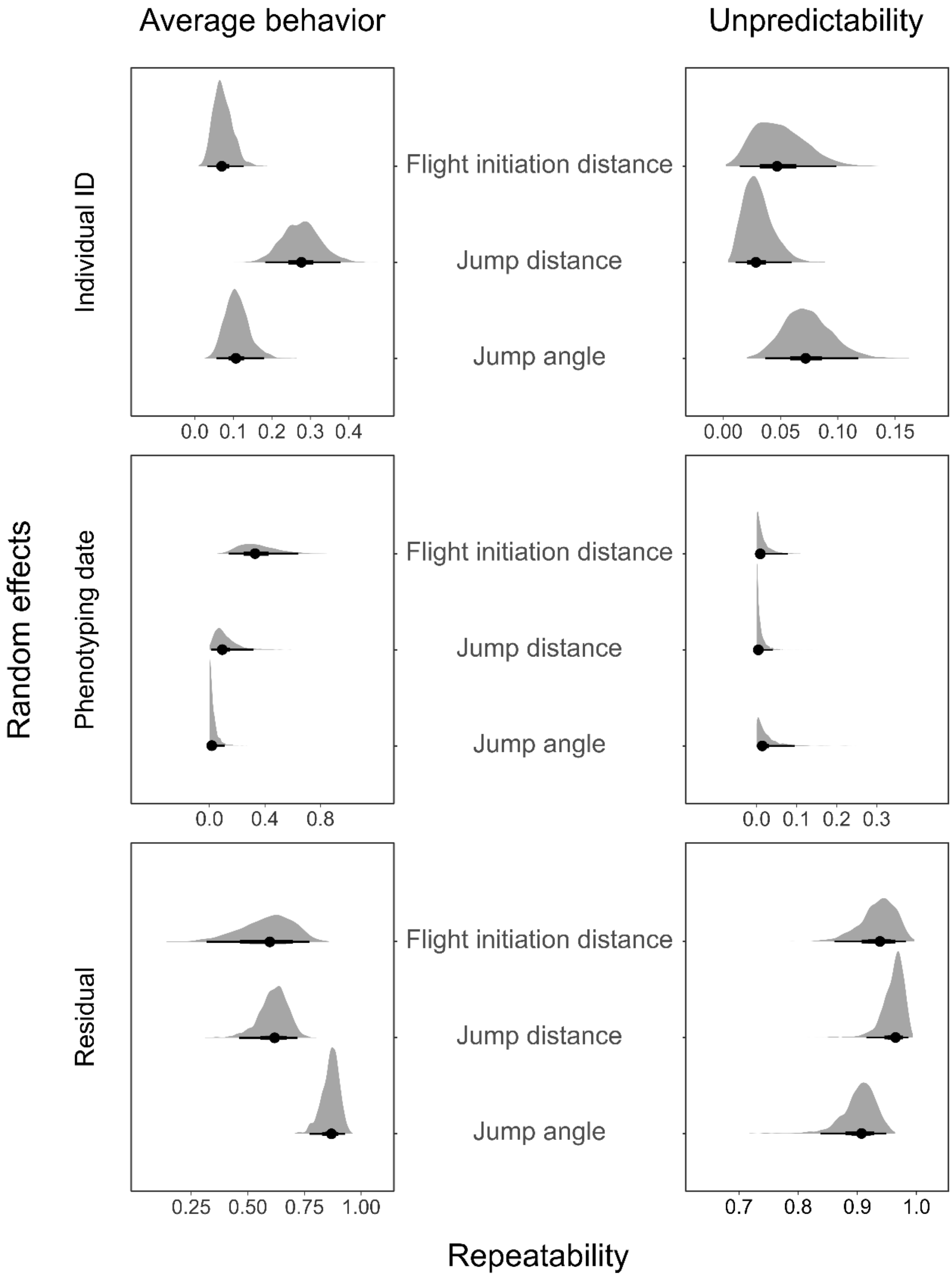

Table S3: Correlation values from the multivariate double-hierarchical generalized linear model applied to three aspects of escape behavior in steppe grasshoppers *Chorthippus dorsatus*.

**Individual ID**					
			Median estimate	Est. Error	95% HPD interval
Flight initiation distance average	x	Flight initiation distance (un)predictability	-0.2674	0.1828	[-0.6327 : 0.0678]
Flight initiation distance average	x	Jump distance average	0.2992	0.1391	[0.0452 : 0.5843]
Flight initiation distance (un)predictability	x	Jump distance average	0.3641	0.1402	[0.0720 : 0.6079]
Flight initiation distance average	x	Jump distance (un)predictability	0.0715	0.1892	[-0.3314 : 0.4139]
Flight initiation distance (un)predictability	x	Jump distance (un)predictability	0.4020	0.1849	[0.0193 : 0.7412]
Jump distance average	x	Jump distance (un)predictability	0.6008	0.1363	[0.3170 : 0.8407]
Flight initiation distance average	x	Jump angle average	0.2543	0.1689	[-0.0871 : 0.5686]
Flight initiation distance (un)predictability	x	Jump angle average	-0.2386	0.1707	[-0.5485 : 0.1184]
Jump distance average	x	Jump angle average	-0.2106	0.1428	[-0.4762 : 0.0777]
Jump distance (un)predictability	x	Jump angle average	-0.3860	0.1788	[-0.7052 : -0.0134]
Flight initiation distance average	x	Jump angle (un)predictability	-0.3706	0.1735	[-0.7192 : -0.0461]
Flight initiation distance (un)predictability	x	Jump angle (un)predictability	0.1415	0.1890	[-0.2211 : 0.5043]
Jump distance average	x	Jump angle (un)predictability	0.0333	0.1524	[-0.2474 : 0.3565]
Jump distance (un)predictability	x	Jump angle (un)predictability	-0.2702	0.1903	[-0.6323 : 0.0977]
Jump angle average	x	Jump angle (un)predictability	-0.2690	0.1836	[-0.6300 : 0.0777]
**Phenotyping date**					
			Median estimate	Est. Error	95% HPD interval
Flight initiation distance average	x	Flight initiation distance (un)predictability	-0.0154	0.3393	[-0.6556 : 0.6346]
Flight initiation distance average	x	Jump distance average	-0.3902	0.2888	[-0.8495 : 0.1911]
Flight initiation distance (un)predictability	x	Jump distance average	0.1173	0.3525	[-0.5777 : 0.7349]
Flight initiation distance average	x	Jump distance (un)predictability	-0.0169	0.3590	[-0.7190 : 0.6193]
Flight initiation distance (un)predictability	x	Jump distance (un)predictability	0.2423	0.3685	[-0.4955 : 0.8658]

Jump distance average	x	Jump distance (un)predictability	-0.0342	0.3609	[-0.6723 : 0.6817]
Flight initiation distance average	x	Jump angle average	-0.1759	0.3477	[-0.7906 : 0.4827]
Flight initiation distance (un)predictability	x	Jump angle average	0.0499	0.3658	[-0.6232 : 0.7393]
Jump distance average	x	Jump angle average	0.0990	0.3452	[-0.5634 : 0.7097]
Jump distance (un)predictability	x	Jump angle average	0.1134	0.3591	[-0.6137 : 0.7401]
Flight initiation distance average	x	Jump angle (un)predictability	-0.2481	0.3411	[-0.8465 : 0.4263]
Flight initiation distance (un)predictability	x	Jump angle (un)predictability	0.0454	0.3602	[-0.6665 : 0.6954]
Jump distance average	x	Jump angle (un)predictability	0.0015	0.3491	[-0.6277 : 0.6612]
Jump distance (un)predictability	x	Jump angle (un)predictability	0.1456	0.3616	[-0.5158 : 0.8420]
Jump angle average	x	Jump angle (un)predictability	0.2171	0.3611	[-0.4803 : 0.8399]

Residual correlations

			Median estimate	Est. Error	95% HPD interval
Flight initiation distance average	x	Jump distance average	-0.0188	0.0364	[-0.0914 : 0.0505]
Flight initiation distance average	x	Jump angle average	0.0251	0.0363	[-0.0494 : 0.0920]
Jump distance average	x	Jump angle average	0.0004	0.0364	[-0.0692 : 0.0713]
