# Supplementary material for: Condition dependence of (un)predictability in escape behavior of a grasshopper species
Source: Behav Ecol. 2023 Jun 13;34(5):741–50. doi: 10.1093/beheco/arad047 (PMC10516674; doi:10.1093/beheco/arad047)
Supplement: arad047_suppl_Supplementary_Material_2 [file arad047_suppl_supplementary_material_2.html]

Condition-dependence of (un)predictability in escape behavior of a grasshopper species


# Condition-dependence of (un)predictability in escape behavior of a grasshopper species

### Supplement 2: analyses

#### 2023-04-04

- Loading
  packages
- Preparing
  dataset
- Blind
  model tuning
- Jumps vs
  non-jumps
- Data visualization and
  transformations
  - Descriptive
    stats
- Analysis of
  (un)predictability: two-steps approach
  - Data
    preparation
    - Checking
      correlation between sd and average values
    - Models
      - FID
      - Jump
        Distance
      - Jump Angle
- Multivariate
  double hierarchical mixed-effects model (DHGLM)
  - Models
  - Variance decomposition
    - Equations
    - FID
    - Jump
      Distance
    - Jump Angle
  - Coefficient of individual
    variation
    - Equations
    - FID
    - Jump
      Distance
    - Jump Angle
  - Results
  - Model fit
    checks
    - Gelman-Rubin convergence
      criteria
    - Effective sample size
    - Trace plots
      - FID
      - Jump
        Distance
      - Jump Angle
    - Posterior predictive checks
      - FID
      - Jump
        Distance
      - Jump Angle
    - Prior samples
      - FID
      - Jump
        Distance
      - Jump Angle
  - Figures
    - Fixed Effects
      - FID
      - JumpDistance
      - Jump Angle
    - Correlations
    - Random Effects
      - FID
      - Jump
        Distance
      - Jump Angle
    - Repeatabilities
      - IndID
      - Phenotyping
        Date
      - Residual
- References

---

##### Loading packages

Install also R tools 4.0: https://cran.r-project.org/bin/windows/Rtools/rtools40.html

```
library(plyr);library(tidyverse); library(tidybayes);library(brms); 
library(parallel);library(coda); library(ggdist);library(rstan); 
library(bayesplot);library(ggpubr); library(broom)
```

Print versions to improve reproducibility

```
print(sessionInfo(), locale = FALSE, RNG = TRUE)
```

```
## R version 4.1.3 (2022-03-10)
## Platform: x86_64-w64-mingw32/x64 (64-bit)
## Running under: Windows 10 x64 (build 19045)
## 
## Matrix products: default
## 
## Random number generation:
##  RNG:     Mersenne-Twister 
##  Normal:  Inversion 
##  Sample:  Rejection 
##  
## attached base packages:
## [1] parallel  stats     graphics  grDevices utils     datasets  methods  
## [8] base     
## 
## other attached packages:
##  [1] broom_1.0.3          ggpubr_0.5.0         bayesplot_1.10.0    
##  [4] rstan_2.21.7         StanHeaders_2.21.0-7 ggdist_3.2.0        
##  [7] coda_0.19-4          brms_2.18.0          Rcpp_1.0.9          
## [10] tidybayes_3.0.2      forcats_0.5.2        stringr_1.4.1       
## [13] dplyr_1.0.10         purrr_0.3.5          readr_2.1.3         
## [16] tidyr_1.2.1          tibble_3.1.8         ggplot2_3.4.0       
## [19] tidyverse_1.3.2      plyr_1.8.8          
## 
## loaded via a namespace (and not attached):
##   [1] googledrive_2.0.0    colorspace_2.0-3     ggsignif_0.6.4      
##   [4] ellipsis_0.3.2       markdown_1.4         base64enc_0.1-3     
##   [7] fs_1.5.2             rstudioapi_0.14      farver_2.1.1        
##  [10] svUnit_1.0.6         DT_0.26              fansi_1.0.3         
##  [13] mvtnorm_1.1-3        lubridate_1.9.0      xml2_1.3.3          
##  [16] codetools_0.2-18     bridgesampling_1.1-2 cachem_1.0.6        
##  [19] knitr_1.41           shinythemes_1.2.0    jsonlite_1.8.3      
##  [22] dbplyr_2.2.1         shiny_1.7.3          compiler_4.1.3      
##  [25] httr_1.4.4           backports_1.4.1      assertthat_0.2.1    
##  [28] Matrix_1.5-3         fastmap_1.1.0        gargle_1.2.1        
##  [31] cli_3.4.1            later_1.3.0          prettyunits_1.1.1   
##  [34] htmltools_0.5.3      tools_4.1.3          igraph_1.3.5        
##  [37] gtable_0.3.1         glue_1.6.2           reshape2_1.4.4      
##  [40] posterior_1.3.1      carData_3.0-5        cellranger_1.1.0    
##  [43] jquerylib_0.1.4      vctrs_0.5.1          nlme_3.1-155        
##  [46] crosstalk_1.2.0      tensorA_0.36.2       xfun_0.35           
##  [49] ps_1.7.2             rvest_1.0.3          timechange_0.1.1    
##  [52] mime_0.12            miniUI_0.1.1.1       lifecycle_1.0.3     
##  [55] gtools_3.9.4         rstatix_0.7.1        googlesheets4_1.0.1 
##  [58] zoo_1.8-11           scales_1.2.1         colourpicker_1.2.0  
##  [61] hms_1.1.2            promises_1.2.0.1     Brobdingnag_1.2-9   
##  [64] inline_0.3.19        shinystan_2.6.0      yaml_2.3.6          
##  [67] gridExtra_2.3        loo_2.5.1            sass_0.4.4          
##  [70] stringi_1.7.6        dygraphs_1.1.1.6     checkmate_2.1.0     
##  [73] pkgbuild_1.4.0       rlang_1.0.6          pkgconfig_2.0.3     
##  [76] matrixStats_0.63.0   distributional_0.3.1 evaluate_0.18       
##  [79] lattice_0.20-45      rstantools_2.2.0     htmlwidgets_1.5.4   
##  [82] processx_3.8.0       tidyselect_1.2.0     magrittr_2.0.3      
##  [85] R6_2.5.1             generics_0.1.3       DBI_1.1.3           
##  [88] pillar_1.8.1         haven_2.5.1          withr_2.5.0         
##  [91] xts_0.12.2           abind_1.4-5          car_3.1-1           
##  [94] modelr_0.1.10        crayon_1.5.2         arrayhelpers_1.1-0  
##  [97] utf8_1.2.2           tzdb_0.3.0           rmarkdown_2.18      
## [100] grid_4.1.3           readxl_1.4.1         callr_3.7.3         
## [103] threejs_0.3.3        reprex_2.0.2         digest_0.6.30       
## [106] xtable_1.8-4         httpuv_1.6.6         RcppParallel_5.1.5  
## [109] stats4_4.1.3         munsell_0.5.0        bslib_0.4.1         
## [112] shinyjs_2.1.0
```

---

#### Preparing dataset

Loading dataset, which is referred as “md” from now on.

```
rm(list=ls()) #removes everything in the environment
md=read.table(choose.files(),h=T,sep="")
```

---

#### Blind model tuning

When tuning the models for first time, we used a randomized vector
for the treatment variable in order to avoid unconsciously picking
variables that favor an outcome that fit our expectations for treatment
effects. We do not present here the analysis made with the randomized
vector, but, for replicability, we present the code for randomizing the
treatment vector, which can then be used for tuning the models being
blind to the real treatment effects.

```
md = md %>% 
  mutate(TreatmentRandom = sample(Treatment))
```

---

#### Jumps vs non-jumps

Chi-squared test for number of jumps (J) and non-jumps (N) per
treatment (C= control, T= treatment)

```
table(md$Treatment,md$JumpYN)
```

```
##    
##       N   Y
##   C  77 501
##   T 120 465
```

```
chisq.test(md$Treatment, md$JumpYN, correct=FALSE)
```

```
## 
##  Pearson's Chi-squared test
## 
## data:  md$Treatment and md$JumpYN
## X-squared = 10.686, df = 1, p-value = 0.00108
```

**Removing non-jumps**

```
md=subset(md, !JumpDistance==0)
```

---

### Data visualization and transformations

**Flight Initiation Distance (FID)**

```
ggplot(md, aes(x=FID))+
  geom_histogram(color="#929292", fill="#E4E4E4",bins=30)+
labs(x="Flight initiation distance (cm)", y="Count")+
theme_test()
```

Log transformation of FID

```
md$logFID=log(md$FID)

ggplot(md, aes(x=logFID))+
  geom_histogram(color="#929292", fill="#E4E4E4",bins=30)+
labs(x="Flight initiation distance (log)", y="Count")+
theme_test()
```

Create a variable with the average individual log FID

```
md$AvIndlogFID = ave(md$logFID, md$IndID)
```

**Jump Distance**

```
ggplot(md, aes(x=JumpDistance))+
  geom_histogram(color="#929292", fill="#E4E4E4",bins=30)+
labs(x="Jump distance (cm)", y="Count")+
theme_test()
```

Create a variable with the average individual log FID

```
md$AvIndJumpDistance = ave(md$JumpDistance, md$IndID)
```

**Jump Angle**

```
ggplot(md, aes(x=JumpAngle))+
  geom_histogram(color="#929292", fill="#E4E4E4",bins=30)+
labs(x="Jump angle (°)", y="Count")+
theme_test()
```

**Day Time**

0 centered to average time

```
md$DayTimeC=c(scale(md$DayTime,scale=F))

ggplot(md, aes(x=DayTimeC))+
  geom_histogram(color="#929292", fill="#E4E4E4",bins=30)+
labs(x="Daytime (centered)", y="Count")+
theme_test()
```

**Jump order** 0 centered to average jump

```
md = md %>% 
  mutate(JumpOrderC = recode(JumpOrder, 
          "1" = -4.5, "2" = -3.5, "3" = -2.5, "4" = -1.5, "5" = -0.5, 
          "6" = 0.5, "7" = 1.5, "8" = 2.5, "9" = 3.5, "10" = 4.5))

ggplot(md, aes(factor(JumpOrderC))) +
geom_bar(stat="count", color="#929292", fill="#E4E4E4") +
labs(x="Jump order (centered)", y="Count")+
theme_test()
```

**Sex**

Female = -0.5 Male = 0.5

```
md = md %>% 
  mutate(SexC = recode(Sex, "F" = -0.5, "M" = 0..5))

table((ddply(md, .(SexC, IndID), nrow))$SexC)
```

```
## 
## -0.5  0.5 
##   70   61
```

**Treatment**

Control = 0 Immune-challenge treatment = 1

```
md = md %>% 
  mutate(TreatmentC = recode(Treatment, "C" = 0, "T" = 1))

table((ddply(md, .(TreatmentC, IndID), nrow))$TreatmentC)
```

```
## 
##  0  1 
## 66 65
```

#### Descriptive stats

```
DescriptiveStats = md %>% 
  select(Treatment, FID, JumpDistance,JumpAngle) %>% 
  group_by(Treatment) %>% 
  summarize_all(list(Mean = mean,SD = sd), na.rm = TRUE) %>% 
  pivot_longer(cols = 2:7, names_to = "Trait", values_to= "Value") %>%
  mutate(Value = round(Value, digits = 2)) %>% 
separate(col = Trait, into = c("Trait","Stat"), sep ="_") %>% 
  pivot_wider(names_from = "Stat", values_from = "Value") %>% 
  arrange(Trait) %>% 
  select(Trait, Treatment, everything());DescriptiveStats
```

---

## Analysis of (un)predictability: two-steps approach

Analysis of treatment effect on (un)predictability, using individual
mean values for average behavior and standard deviations/coefficient of
variation for (un)predictability.

### Data preparation

Calculating average and standard deviation values per individual:

```
mdSumm = md %>%
    select(IndID, SexC, Treatment, TreatmentC, PairID, FID, JumpDistance,JumpAngle) %>%
    group_by(IndID) %>%
    mutate(AvFID = mean(FID), 
           AvJumpDistance = mean(JumpDistance), 
           AvJumpAngle = mean(JumpAngle),
           SDFID = sd(FID),
           SDJumpDistance = sd(JumpDistance),
           SDJumpAngle = sd(JumpAngle),
           FID=NULL,
           JumpDistance=NULL,
           JumpAngle=NULL)%>%
    distinct()
```

#### Checking correlation between sd and average values

**FID**

```
ggplot(mdSumm, aes(x=AvFID, y=SDFID))+
  geom_point(fill="#E4E4E4",na.rm = TRUE)+
  geom_smooth(method=lm, color="#929292",na.rm = TRUE)+
  stat_cor()+
 labs(x="Individual FID average (cm)", y="Individual FID standard deviation (cm)")+
  theme_test()
```

**Jump Distance**

```
ggplot(mdSumm, aes(x=AvJumpDistance, y=SDJumpDistance))+
  geom_point(fill="#E4E4E4",na.rm = TRUE)+
  geom_smooth(method=lm, color="#929292",na.rm = TRUE)+
  stat_cor()+
 labs(x="Individual jump distance average (cm)", y="Individual jump distance standard deviation (cm)")+
  theme_test()
```

**Jump Angle**

```
ggplot(mdSumm, aes(x=AvJumpAngle, y=SDJumpAngle))+
  geom_point(fill="#E4E4E4",na.rm = TRUE)+
  geom_smooth(method=lm, color="#929292",na.rm = TRUE)+
  stat_cor()+
 labs(x="Individual jump angle average (°)", y="Individual jump angle standard deviation (°)")+
  theme_test()
```

For the traits that had a correlation between individual average and
individual standard deviation (FID and jump distance), we additionally
calculate the coefficient of variation.

```
mdSumm$CVFID=mdSumm$SDFID/mdSumm$AvFID
mdSumm$CVJumpDistance=mdSumm$SDJumpDistance/mdSumm$AvJumpDistance
```

---

#### Models

##### FID

**Average behavior**

```
#modAvFID = brm((bf(AvFID ~ TreatmentC + SexC + (1|PairID))), data = mdSumm, warmup = 4500,iter = 34500, thin=30, chains = 2, init = "random", seed = 12345, cores = detectCores(), control = list(adapt_delta = 0.9))

#save(modAvFID, file=paste0("modAvFID_230201.RData"))
load("modAvFID_230201.RData")
```

```
EstmodAvFID = summarise_draws(as.data.frame(modAvFID)[,1:5]) %>% 
  select(variable, median, sd, rhat, ess_bulk) 
  
HPDmodAvFID = as.data.frame(HPDinterval(as.mcmc(as.data.frame(modAvFID)[,1:5], combine_chains = TRUE))) %>% 
  rename(lower_HPD = lower, upper_HPD = upper) %>% 
  rownames_to_column(var = "variable")

EstmodAvFID = left_join(EstmodAvFID, HPDmodAvFID, by = "variable");EstmodAvFID
```

```
ggplot(mdSumm, aes(x=Treatment, y=AvFID, fill=Treatment)) +
  geom_violin(alpha=0.7,width=0.6,trim = FALSE) +
  scale_fill_manual(values=c("#126782", "#FB8500"))+ 
  scale_x_discrete(labels=c("Control","Treatment"))+
  geom_boxplot(alpha=1, width = 0.1)+
 labs(x="Treatment", y="Individual flight initiation distance average (cm)")+
  theme_test()+
  theme(legend.position="none")
```

**(Un)predictability (SD)**

```
#modSDFID = brm((bf(SDFID ~ TreatmentC + SexC + (1|PairID))), data = mdSumm, warmup = 4500,iter = 34500, thin=30, chains = 2, init = "random", seed = 12345, cores = detectCores(), control = list(adapt_delta = 0.9))

#save(modSDFID, file=paste0("modSDFID_230201.RData"))
load("modSDFID_230201.RData")
```

```
EstmodSDFID = summarise_draws(as.data.frame(modSDFID)[,1:5]) %>% 
  select(variable, median, sd, rhat, ess_bulk) 
  
HPDmodSDFID = as.data.frame(HPDinterval(as.mcmc(as.data.frame(modSDFID)[,1:5], combine_chains = TRUE))) %>% 
  rename(lower_HPD = lower, upper_HPD = upper) %>% 
  rownames_to_column(var = "variable")

 
EstmodSDFID = left_join(EstmodSDFID, HPDmodSDFID, by = "variable");EstmodSDFID
```

```
ggplot(mdSumm, aes(x=Treatment, y=SDFID, fill=Treatment)) +
  geom_violin(alpha=0.7,width=0.6,trim = FALSE) +
  scale_fill_manual(values=c("#126782", "#FB8500"))+ 
  scale_x_discrete(labels=c("Control","Treatment"))+
  geom_boxplot(alpha=1, width = 0.1)+
 labs(x="Treatment", y="Individual flight initiation distance standard deviation (cm)")+
  theme_test()+
  theme(legend.position="none")
```

**(Un)predictability (CV)**

```
#modCVFID = brm((bf(CVFID ~ TreatmentC + SexC + (1|PairID))), data = mdSumm, warmup = 4500,iter = 34500, thin=30, chains = 2, init = "random", seed = 12345, cores = detectCores(), control = list(adapt_delta = 0.9))

#save(modCVFID, file=paste0("modCVFID_230201.RData"))
load("modCVFID_230201.RData")
```

```
EstmodCVFID = summarise_draws(as.data.frame(modCVFID)[,1:5]) %>% 
  select(variable, median, sd, rhat, ess_bulk) 
  
HPDmodCVFID = as.data.frame(HPDinterval(as.mcmc(as.data.frame(modCVFID)[,1:5], combine_chains = TRUE))) %>% 
  rename(lower_HPD = lower, upper_HPD = upper) %>% 
  rownames_to_column(var = "variable")

 
EstmodCVFID = left_join(EstmodCVFID, HPDmodCVFID, by = "variable");EstmodCVFID
```

```
ggplot(mdSumm, aes(x=Treatment, y=CVFID, fill=Treatment)) +
  geom_violin(alpha=0.7,width=0.6,trim = FALSE) +
  scale_fill_manual(values=c("#126782", "#FB8500"))+ 
  scale_x_discrete(labels=c("Control","Treatment"))+
  geom_boxplot(alpha=1, width = 0.1)+
 labs(x="Treatment", y="Individual flight initiation distance coefficient of variation")+
  theme_test()+
  theme(legend.position="none")
```

---

##### Jump Distance

**Average behavior**

```
#modAvJumpDistance = brm((bf(AvJumpDistance ~ TreatmentC + SexC + (1|PairID))), data = mdSumm, warmup = 4500,iter = 34500, thin=30, chains = 2, init = "random", seed = 12345, cores = detectCores(), control = list(adapt_delta = 0.9))

#save(modAvJumpDistance,file=paste0("modAvJumpDistance_230201.RData"))
load("modAvJumpDistance_230201.RData")
```

```
EstmodAvJumpDistance = summarise_draws(as.data.frame(modAvJumpDistance)[,1:5]) %>%   select(variable, median, sd, rhat, ess_bulk) 
  
HPDmodAvJumpDistance = as.data.frame(HPDinterval(as.mcmc(as.data.frame(modAvJumpDistance)[,1:5], combine_chains = TRUE))) %>% 
  rename(lower_HPD = lower, upper_HPD = upper) %>% 
  rownames_to_column(var = "variable")

 
EstmodAvJumpDistance = left_join(EstmodAvJumpDistance, HPDmodAvJumpDistance, by = "variable"); EstmodAvJumpDistance
```

```
ggplot(mdSumm, aes(x=Treatment, y=AvJumpDistance, fill=Treatment)) +
  geom_violin(alpha=0.7,width=0.6,trim = FALSE) +
  scale_fill_manual(values=c("#126782", "#FB8500"))+ 
  scale_x_discrete(labels=c("Control","Treatment"))+
  geom_boxplot(alpha=1, width = 0.1)+
 labs(x="Treatment", y="Individual jump distance average (cm)")+
  theme_test()+
  theme(legend.position="none")
```

**(Un)predictability (SD)**

```
#modSDJumpDistance = brm((bf(SDJumpDistance ~ TreatmentC + SexC + (1|PairID))), data = mdSumm, warmup = 4500,iter = 34500, thin=30, chains = 2, init = "random", seed = 12345, cores = detectCores(), control = list(adapt_delta = 0.9))

#save(modSDJumpDistance,file=paste0("modSDJumpDistance_230201.RData"))
load("modSDJumpDistance_230201.RData")
```

```
EstmodSDJumpDistance = summarise_draws(as.data.frame(modSDJumpDistance)[,1:5]) %>%
  select(variable, median, sd, rhat, ess_bulk) 
  
HPDmodSDJumpDistance = as.data.frame(HPDinterval(as.mcmc(as.data.frame(modSDJumpDistance)[,1:5], combine_chains = TRUE))) %>% 
  rename(lower_HPD = lower, upper_HPD = upper) %>% 
  rownames_to_column(var = "variable")


EstmodSDJumpDistance=left_join(EstmodSDJumpDistance, HPDmodSDJumpDistance, by = "variable");EstmodSDJumpDistance
```

```
ggplot(mdSumm, aes(x=Treatment, y=SDJumpDistance, fill=Treatment)) +
  geom_violin(alpha=0.7,width=0.6,trim = FALSE) +
  scale_fill_manual(values=c("#126782", "#FB8500"))+ 
  scale_x_discrete(labels=c("Control","Treatment"))+
  geom_boxplot(alpha=1, width = 0.1)+
 labs(x="Treatment", y="Individual jump distance standard deviation (cm)")+
  theme_test()+
  theme(legend.position="none")
```

**(Un)predictability (CV)**

```
#modCVJumpDistance = brm((bf(CVJumpDistance ~ TreatmentC + SexC + (1|PairID))), data = mdSumm, warmup = 4500,iter = 34500, thin=30, chains = 2, init = "random", seed = 12345, cores = detectCores(), control = list(adapt_delta = 0.9))

#save(modCVJumpDistance,file=paste0("modCVJumpDistance_230201.RData"))
load("modCVJumpDistance_230201.RData")
```

```
EstmodCVJumpDistance = summarise_draws(as.data.frame(modCVJumpDistance)[,1:5]) %>%
  select(variable, median, sd, rhat, ess_bulk) 
  
HPDmodCVJumpDistance = as.data.frame(HPDinterval(as.mcmc(as.data.frame(modCVJumpDistance)[,1:5], combine_chains = TRUE))) %>% 
  rename(lower_HPD = lower, upper_HPD = upper) %>% 
  rownames_to_column(var = "variable")


EstmodCVJumpDistance = left_join(EstmodCVJumpDistance, HPDmodCVJumpDistance, by = "variable");EstmodCVJumpDistance
```

```
ggplot(mdSumm, aes(x=Treatment, y=CVJumpDistance, fill=Treatment)) +
  geom_violin(alpha=0.7,width=0.6,trim = FALSE) +
  scale_fill_manual(values=c("#126782", "#FB8500"))+ 
  scale_x_discrete(labels=c("Control","Treatment"))+
  geom_boxplot(alpha=1, width = 0.1)+
 labs(x="Treatment", y="Individual jump distance coefficient of variation")+
  theme_test()+
  theme(legend.position="none")
```

---

##### Jump Angle

**Average behavior**

```
#modAvJumpAngle = brm((bf(AvJumpAngle ~ TreatmentC + SexC + (1|PairID))), data = mdSumm, warmup = 4500,iter = 34500, thin=30, chains = 2, init = "random", seed = 12345, cores = detectCores(), control = list(adapt_delta = 0.9))

#save(modAvJumpAngle,file=paste0("modAvJumpAngle_230201.RData"))
load("modAvJumpAngle_230201.RData")
```

```
EstmodAvJumpAngle = summarise_draws(as.data.frame(modAvJumpAngle)[,1:5]) %>% 
  select(variable, median, sd, rhat, ess_bulk) 

HPDmodAvJumpAngle = as.data.frame(HPDinterval(as.mcmc(as.data.frame(modAvJumpAngle)[,1:5], combine_chains = TRUE))) %>% 
  rename(lower_HPD = lower, upper_HPD = upper) %>% 
  rownames_to_column(var = "variable")


EstmodAvJumpAngle = left_join(EstmodAvJumpAngle, HPDmodAvJumpAngle, by = "variable"); EstmodAvJumpAngle
```

```
ggplot(mdSumm, aes(x=Treatment, y=AvJumpAngle, fill=Treatment)) +
  geom_violin(alpha=0.7,width=0.6,trim = FALSE) +
  scale_fill_manual(values=c("#126782", "#FB8500"))+ 
  scale_x_discrete(labels=c("Control","Treatment"))+
  geom_boxplot(alpha=1, width = 0.1)+
 labs(x="Treatment",y="Individual jump angle average (°)")+
  theme_test()+
  theme(legend.position="none")
```

**(Un)predictability (SD)**

```
#modSDJumpAngle = brm((bf(SDJumpAngle ~ TreatmentC + SexC + (1|PairID))), data = mdSumm, warmup = 4500,iter = 34500, thin=30, chains = 2, init = "random", seed = 12345, cores = detectCores(), control = list(adapt_delta = 0.9))

#save(modSDJumpAngle,file=paste0("modSDJumpAngle_230201.RData"))
load("modSDJumpAngle_230201.RData")
```

```
EstmodSDJumpAngle = summarise_draws(as.data.frame(modSDJumpAngle)[,1:5]) %>% 
  select(variable, median, sd, rhat, ess_bulk)
  
HPDmodSDJumpAngle = as.data.frame(HPDinterval(as.mcmc(as.data.frame(modSDJumpAngle)[,1:5], combine_chains = TRUE))) %>% 
  rename(lower_HPD = lower, upper_HPD = upper) %>% 
  rownames_to_column(var = "variable")


EstmodSDJumpAngle = left_join(EstmodSDJumpAngle, HPDmodSDJumpAngle, by = "variable"); EstmodSDJumpAngle
```

```
ggplot(mdSumm, aes(x=Treatment, y=SDJumpAngle, fill=Treatment)) +
  geom_violin(alpha=0.7,width=0.6,trim = FALSE) +
  scale_fill_manual(values=c("#126782", "#FB8500"))+ 
  scale_x_discrete(labels=c("Control","Treatment"))+
  geom_boxplot(alpha=1, width = 0.1)+
 labs(x="Treatment",y="Individual jump angle standard deviation (°)")+
  theme_test()+
  theme(legend.position="none")
```

---

## Multivariate double hierarchical mixed-effects model (DHGLM)

### Models

| Item | Description |
| --- | --- |
| \(y^{t1}\_{ji}\) | logFID of individual \(j\) on instance \(i\) |
| \(y^{t2}\_{ji}\) | jump distance of individual \(j\) on instance \(i\) |
| \(y^{t3}\_{ji}\) | jump angle of individual \(j\) on instance \(i\) |
| \(\beta\_{m0}\) | population intercept for the mean model |
| \(\beta\_{sd0,exp}\) | population intercept for the dispersion model, estimated on the ln scale |
| \(x\_{1j}\) | input variable for the treatment group of individual \(j\) (\(X\_{1j} = 0\) for control, \(1\) for treatment) |
| \(x\_{2j}\) | input variable for sex of individual \(j\) (\(X\_{2j} = -0.5\) for female, \(0.5\) for male) |
| \(x\_{3ji}\) | input variable for daytime for individual \(j\) on instance \(i\) |
| \(x\_{4ji}\) | input variable for jump order for individual \(j\) on instance \(i\) |
| \(e\) | residual error |
| \(\sigma^2\_{e\_{ij}}\) | residual variance for double hierarchical models: unique value for individual \(j\) on instance \(i\) |
| \(ID\_{m0j}\) | difference between the population intercept \(\beta\_{m0}\) and the random intercept for individual \(j\) for the mean model |
| \(ID\_{sd0j,exp}\) | difference between the population intercept \(\beta\_{sd0,exp}\) and the random intercept for individual \(j\) for the dispersion model, estimated on the ln scale |
| \(Date\_{m0d}\) | difference between the population intercept \(\beta\_{m0}\) and the random intercept for phenotyping date \(d\) for the mean model |
| \(Date\_{sd0d,exp}\) | difference between the population intercept \(\beta\_{sd0,exp}\) and the random intercept for phenotyping date \(d\) for the dispersion model, estimated on the ln scale |
| \(\rho\) | correlation between random variables |

**Models for the average traits**

*FID* \[ y^{t1}\_{ji} =
(\beta^{t1}\_{m0}+ID^{t1}\_{m0j}+Date^{t1}\_{m0d}) + \beta^{t1}\_{m1}x\_{1j}
+
\beta^{t1}\_{m\_{2}}x\_{2j}+\beta^{t1}\_{m\_{3}}x\_{3ji}+\beta^{t1}\_{m\_{4}}x\_{4ji}+\beta^{t1}\_{m\_{5}}x\_{1j}x\_{2j}+e^{t1}\_{ji},\]

*Jump distance* \[ y^{t2}\_{ji} =
(\beta^{t2}\_{m0}+ID^{t2}\_{m0j}+Date^{t2}\_{m0d}) + \beta^{t2}\_{m1}x\_{1j}
+
\beta^{t2}\_{m\_{2}}x\_{2j}+\beta^{t2}\_{m\_{3}}x\_{3ji}+\beta^{t2}\_{m\_{4}}x\_{4ji}+\beta^{t2}\_{m\_{5}}x\_{1j}x\_{2j}+e^{t2}\_{ji},\]

*Jump angle* \[ y^{t3}\_{ji} =
(\beta^{t3}\_{m0}+ID^{t3}\_{m0j}+Date^{t3}\_{m0d}) + \beta^{t3}\_{m1}x\_{1j}
+
\beta^{t3}\_{m\_{2}}x\_{2j}+\beta^{t3}\_{m\_{3}}x\_{3ji}+\beta^{t3}\_{m\_{4}}x\_{4ji}+\beta^{t3}\_{m\_{5}}x\_{1j}x\_{2j}+e^{t3}\_{ji},\]

Based on equations 25-26 in O’Dea et al, 2022.

**Models for the variance in traits**

*FID* \[ ln(\sigma^2\_{e^{t1}\_{ji}})
= (\beta^{t1}\_{sd0,exp}+ID^{t1}\_{sd0j,exp}+Date^{t1}\_{sd0d,exp}) +
\beta^{t1}\_{sd\_{1},exp}x\_{1j}+\beta^{t1}\_{sd\_{2},exp}x\_{2j}+\beta^{t1}\_{sd\_{3},exp}x\_{3ji}+\beta^{t1}\_{sd\_{4},exp}x\_{4ji}
+ \beta^{t1}\_{sd\_{5},exp}x\_{1j}x\_{2j},\]

*Jump distance* \[
ln(\sigma^2\_{e^{t2}\_{ji}})
= (\beta^{t2}\_{sd0,exp}+ID^{t2}\_{sd0j,exp}+Date^{t2}\_{sd0d,exp}) +
\beta^{t2}\_{sd\_{1},exp}x\_{1j}+\beta^{t2}\_{sd\_{2},exp}x\_{2j}+\beta^{t2}\_{sd\_{3},exp}x\_{3ji}+\beta^{t2}\_{sd\_{4},exp}x\_{4ji}
+ \beta^{t2}\_{sd\_{5},exp}x\_{1j}x\_{2j},\]

*Jump angle* \[
ln(\sigma^2\_{e^{t3}\_{ji}})
= (\beta^{t3}\_{sd0,exp}+ID^{t3}\_{sd0j,exp}+Date^{t3}\_{sd0d,exp}) +
\beta^{t3}\_{sd\_{1},exp}x\_{1j}+\beta^{t3}\_{sd\_{2},exp}x\_{2j}+\beta^{t3}\_{sd\_{3},exp}x\_{3ji}+\beta^{t3}\_{sd\_{4},exp}x\_{4ji}
+ \beta^{t3}\_{sd\_{5},exp}x\_{1j}x\_{2j},\]

Based on equations 27-28 in O’Dea et al, 2022.

**Residual variance**

\[ \left[\begin{array}
{r message=FALSE, warning=FALSE}
e^{t1}\_{ji}\\
e^{t2}\_{ji}\\
e^{t3}\_{ji}
\end{array}\right]
\sim MVN
\left(
\left[\begin{array}
{r message=FALSE, warning=FALSE}
0\\
0\\
0
\end{array}\right],
\left[\begin{array}
{rrr}
\sigma^2\_{e^{t1}\_{ji}} &
\rho(e^{t1}\_{ji}, e^{t2}\_{ji})\sigma\_{e^{t1}\_{ji}}\sigma\_{e^{t2}\_{ji}}
&
\rho(e^{t1}\_{ji}, e^{t3}\_{ji})\sigma\_{e^{t1}\_{ji}}\sigma\_{e^{t3}\_{ji}}
\\
..... &
\sigma^2\_{e^{t2}\_{ji}} &
\rho(e^{t2}\_{ji}, e^{t3}\_{ji})\sigma\_{e^{t1}\_{ji}}\sigma\_{e^{t2}\_{ji}}
\\
..... &
..... &
\sigma^2\_{e^{t3}\_{ji}}
\end{array}\right]
\right),\]

Based on equation 29 in O’Dea et al, 2022.

**Variance-covariance matrix**

\[ \left[\begin{array}
{l}
ID^{t1}\_{m0j}\\
ID^{t1}\_{sd0j,exp}\\
Date^{t1}\_{m0d}\\
Date^{t1}\_{sd0d,exp}\\
ID^{t2}\_{m0j}\\
ID^{t2}\_{sd0j,exp}\\
Date^{t2}\_{m0d}\\
Date^{t2}\_{sd0d,exp}\\
ID^{t3}\_{m0j}\\
ID^{t3}\_{sd0j,exp}\\
Date^{t3}\_{m0d}\\
Date^{t3}\_{sd0d,exp}
\end{array}\right]
\sim MVN
\left(
\left[\begin{array}
{r message=FALSE, warning=FALSE}
0\\
0\\
0\\
0\\
0\\
0\\
0\\
0\\
0\\
0\\
0\\
0
\end{array}\right],
\left[\begin{array}
{rrrrrrrrrrrr}
\sigma^2\_{ID^{t1}\_{m0}} &
\rho(ID^{t1}\_{m0j},ID^{t1}\_{sd0j,exp})\sigma\_{ID^{t1}\_{m0j}}
\sigma\_{ID^{t1}\_{sd0j,exp}} &
\rho(ID^{t1}\_{m0j},Date^{t1}\_{m0d})\sigma\_{ID^{t1}\_{m0j}}
\sigma\_{Date^{t1}\_{m0d}} &
\rho(ID^{t1}\_{m0j},Date^{t1}\_{sd0d,exp})\sigma\_{ID^{t1}\_{m0j}}
\sigma\_{Date^{t1}\_{sd0d,exp}} &
\rho(ID^{t1}\_{m0j},ID^{t2}\_{m0j})\sigma\_{ID^{t1}\_{m0j}}
\sigma\_{ID^{t2}\_{m0j}} &
\rho(ID^{t1}\_{m0j},ID^{t2}\_{sd0j,exp})\sigma\_{ID^{t1}\_{m0j}}
\sigma\_{ID^{t2}\_{sd0j,exp}} &
\rho(ID^{t1}\_{m0j},Date^{t2}\_{m0d})\sigma\_{ID^{t1}\_{m0j}}
\sigma\_{Date^{t2}\_{m0d}} &
\rho(ID^{t1}\_{m0j},Date^{t2}\_{sd0d,exp})\sigma\_{ID^{t1}\_{m0j}}
\sigma\_{Date^{t2}\_{sd0d,exp}} &
\rho(ID^{t1}\_{m0j},ID^{t3}\_{m0j})\sigma\_{ID^{t1}\_{m0j}}
\sigma\_{ID^{t3}\_{m0j}} &
\rho(ID^{t1}\_{m0j},ID^{t3}\_{sd0j,exp})\sigma\_{ID^{t1}\_{m0j}}
\sigma\_{ID^{t3}\_{sd0j,exp}} &
\rho(ID^{t1}\_{m0j},Date^{t3}\_{m0d})\sigma\_{ID^{t1}\_{m0j}}
\sigma\_{Date^{t3}\_{m0d}} &
\rho(ID^{t1}\_{m0j},Date^{t3}\_{sd0d,exp})\sigma\_{ID^{t1}\_{m0j}}
\sigma\_{Date^{t3}\_{sd0d,exp}}\\
..... &
\sigma^2\_{ID^{t1}\_{sd0,exp}} &
\rho(ID^{t1}\_{sd0j,exp},Date^{t1}\_{m0d})\sigma\_{ID^{t1}\_{sd0j,exp}}
\sigma\_{Date^{t1}\_{m0d}} &
\rho(ID^{t1}\_{sd0j,exp},Date^{t1}\_{sd0d,exp})\sigma\_{ID^{t1}\_{sd0j,exp}}
\sigma\_{Date^{t1}\_{sd0d,exp}} &
\rho(ID^{t1}\_{sd0j,exp},ID^{t2}\_{m0j})\sigma\_{ID^{t1}\_{sd0j,exp}}
\sigma\_{ID^{t2}\_{m0j}} &
\rho(ID^{t1}\_{sd0j,exp},ID^{t2}\_{sd0j,exp})\sigma\_{ID^{t1}\_{sd0j,exp}}
\sigma\_{ID^{t2}\_{sd0j,exp}} &
\rho(ID^{t1}\_{sd0j,exp},Date^{t2}\_{m0d})\sigma\_{ID^{t1}\_{sd0j,exp}}
\sigma\_{Date^{t2}\_{m0d}} &
\rho(ID^{t1}\_{sd0j,exp},Date^{t2}\_{sd0d,exp})\sigma\_{ID^{t1}\_{sd0j,exp}}
\sigma\_{Date^{t2}\_{sd0d,exp}} &
\rho(ID^{t1}\_{sd0j,exp},ID^{t3}\_{m0j})\sigma\_{ID^{t1}\_{sd0j,exp}}
\sigma\_{ID^{t3}\_{m0j}} &
\rho(ID^{t1}\_{sd0j,exp},ID^{t3}\_{sd0j,exp})\sigma\_{ID^{t1}\_{sd0j,exp}}
\sigma\_{ID^{t3}\_{sd0j,exp}} &
\rho(ID^{t1}\_{sd0j,exp},Date^{t3}\_{m0d})\sigma\_{ID^{t1}\_{sd0j,exp}}
\sigma\_{Date^{t3}\_{m0d}} &
\rho(ID^{t1}\_{sd0j,exp},Date^{t3}\_{sd0d,exp})\sigma\_{ID^{t1}\_{sd0j,exp}}
\sigma\_{Date^{t3}\_{sd0d,exp}}\\
..... &
..... &
\sigma^2\_{Date^{t1}\_{m0}} &
\rho(Date^{t1}\_{m0d},Date^{t1}\_{sd0d,exp})\sigma\_{Date^{t1}\_{m0d}}
\sigma\_{Date^{t1}\_{sd0d,exp}} &
\rho(Date^{t1}\_{m0d},ID^{t2}\_{m0j})\sigma\_{Date^{t1}\_{m0d}}
\sigma\_{ID^{t2}\_{m0j}} &
\rho(Date^{t1}\_{m0d},ID^{t2}\_{sd0j,exp})\sigma\_{Date^{t1}\_{m0d}}
\sigma\_{ID^{t2}\_{sd0j,exp}} &
\rho(Date^{t1}\_{m0d},Date^{t2}\_{m0d})\sigma\_{Date^{t1}\_{m0d}}
\sigma\_{Date^{t2}\_{m0d}} &
\rho(Date^{t1}\_{m0d},Date^{t2}\_{sd0d,exp})\sigma\_{Date^{t1}\_{m0d}}
\sigma\_{Date^{t2}\_{sd0d,exp}} &
\rho(Date^{t1}\_{m0d},ID^{t3}\_{m0j})\sigma\_{Date^{t1}\_{m0d}}
\sigma\_{ID^{t3}\_{m0j}} &
\rho(Date^{t1}\_{m0d},ID^{t3}\_{sd0j,exp})\sigma\_{Date^{t1}\_{m0d}}
\sigma\_{ID^{t3}\_{sd0j,exp}} &
\rho(Date^{t1}\_{m0d},Date^{t3}\_{m0d})\sigma\_{Date^{t1}\_{m0d}}
\sigma\_{Date^{t3}\_{m0d}} &
\rho(Date^{t1}\_{m0d},Date^{t3}\_{sd0d,exp})\sigma\_{Date^{t1}\_{m0d}}
\sigma\_{Date^{t3}\_{sd0d,exp}}\\
..... &
..... &
..... &
\sigma^2\_{Date^{t1}\_{sd0,exp}} &
\rho(Date^{t1}\_{sd0d,exp},ID^{t2}\_{m0j})\sigma\_{Date^{t1}\_{sd0d,exp}}
\sigma\_{ID^{t2}\_{m0j}} &
\rho(Date^{t1}\_{sd0d,exp},ID^{t2}\_{sd0j,exp})\sigma\_{Date^{t1}\_{sd0d,exp}}
\sigma\_{ID^{t2}\_{sd0j,exp}} &
\rho(Date^{t1}\_{sd0d,exp},Date^{t2}\_{m0d})\sigma\_{Date^{t1}\_{sd0d,exp}}
\sigma\_{Date^{t2}\_{m0d}} &
\rho(Date^{t1}\_{sd0d,exp},Date^{t2}\_{sd0d,exp})\sigma\_{Date^{t1}\_{sd0d,exp}}
\sigma\_{Date^{t2}\_{sd0d,exp}} &
\rho(Date^{t1}\_{sd0d,exp},ID^{t3}\_{m0j})\sigma\_{Date^{t1}\_{sd0d,exp}}
\sigma\_{ID^{t3}\_{m0j}} &
\rho(Date^{t1}\_{sd0d,exp},ID^{t3}\_{sd0j,exp})\sigma\_{Date^{t1}\_{sd0d,exp}}
\sigma\_{ID^{t3}\_{sd0j,exp}} &
\rho(Date^{t1}\_{sd0d,exp},Date^{t3}\_{m0d})\sigma\_{Date^{t1}\_{sd0d,exp}}
\sigma\_{Date^{t3}\_{m0d}} &
\rho(Date^{t1}\_{sd0d,exp},Date^{t3}\_{sd0d,exp})\sigma\_{Date^{t1}\_{sd0d,exp}}
\sigma\_{Date^{t3}\_{sd0d,exp}}\\
..... &
..... &
..... &
..... &
\sigma^2\_{ID^{t2}\_{m0}} &
\rho(ID^{t2}\_{m0j},ID^{t2}\_{sd0j,exp})\sigma\_{ID^{t2}\_{m0j}}
\sigma\_{ID^{t2}\_{sd0j,exp}} &
\rho(ID^{t2}\_{m0j},Date^{t2}\_{m0d})\sigma\_{ID^{t2}\_{m0j}}
\sigma\_{Date^{t2}\_{m0d}} &
\rho(ID^{t2}\_{m0j},Date^{t2}\_{sd0d,exp})\sigma\_{ID^{t2}\_{m0j}}
\sigma\_{Date^{t2}\_{sd0d,exp}} &
\rho(ID^{t2}\_{m0j},ID^{t3}\_{m0j})\sigma\_{ID^{t2}\_{m0j}}
\sigma\_{ID^{t3}\_{m0j}} &
\rho(ID^{t2}\_{m0j},ID^{t3}\_{sd0j,exp})\sigma\_{ID^{t2}\_{m0j}}
\sigma\_{ID^{t3}\_{sd0j,exp}} &
\rho(ID^{t2}\_{m0j},Date^{t3}\_{m0d})\sigma\_{ID^{t2}\_{m0j}}
\sigma\_{Date^{t3}\_{m0d}} &
\rho(ID^{t2}\_{m0j},Date^{t3}\_{sd0d,exp})\sigma\_{ID^{t2}\_{m0j}}
\sigma\_{Date^{t3}\_{sd0d,exp}}\\
..... &
..... &
..... &
..... &
..... &
\sigma^2\_{ID^{t2}\_{sd0,exp}} &
\rho(ID^{t2}\_{sd0j,exp},Date^{t2}\_{m0d})\sigma\_{ID^{t2}\_{sd0j,exp}}
\sigma\_{Date^{t2}\_{m0d}} &
\rho(ID^{t2}\_{sd0j,exp},Date^{t2}\_{sd0d,exp})\sigma\_{ID^{t2}\_{sd0j,exp}}
\sigma\_{Date^{t2}\_{sd0d,exp}} &
\rho(ID^{t2}\_{sd0j,exp},ID^{t3}\_{m0j})\sigma\_{ID^{t2}\_{sd0j,exp}}
\sigma\_{ID^{t3}\_{m0j}} &
\rho(ID^{t2}\_{sd0j,exp},ID^{t3}\_{sd0j,exp})\sigma\_{ID^{t2}\_{sd0j,exp}}
\sigma\_{ID^{t3}\_{sd0j,exp}} &
\rho(ID^{t2}\_{sd0j,exp},Date^{t3}\_{m0d})\sigma\_{ID^{t2}\_{sd0j,exp}}
\sigma\_{Date^{t3}\_{m0d}} &
\rho(ID^{t2}\_{sd0j,exp},Date^{t3}\_{sd0d,exp})\sigma\_{ID^{t2}\_{sd0j,exp}}
\sigma\_{Date^{t3}\_{sd0d,exp}}\\
..... &
..... &
..... &
..... &
..... &
..... &
\sigma^2\_{Date^{t2}\_{m0}} &
\rho(Date^{t2}\_{m0d},Date^{t2}\_{sd0d,exp})\sigma\_{Date^{t2}\_{m0d}}
\sigma\_{Date^{t2}\_{sd0d,exp}} &
\rho(Date^{t2}\_{m0d},ID^{t3}\_{m0j})\sigma\_{Date^{t2}\_{m0d}}
\sigma\_{ID^{t3}\_{m0j}} &
\rho(Date^{t2}\_{m0d},ID^{t3}\_{sd0j,exp})\sigma\_{Date^{t2}\_{m0d}}
\sigma\_{ID^{t3}\_{sd0j,exp}} &
\rho(Date^{t2}\_{m0d},Date^{t3}\_{m0d})\sigma\_{Date^{t2}\_{m0d}}
\sigma\_{Date^{t3}\_{m0d}} &
\rho(Date^{t2}\_{m0d},Date^{t3}\_{sd0d,exp})\sigma\_{Date^{t2}\_{m0d}}
\sigma\_{Date^{t3}\_{sd0d,exp}}\\
..... &
..... &
..... &
..... &
..... &
..... &
..... &
\sigma^2\_{Date^{t2}\_{sd0,exp}} &
\rho(Date^{t2}\_{sd0d,exp},ID^{t3}\_{m0j})\sigma\_{Date^{t2}\_{sd0d,exp}}
\sigma\_{ID^{t3}\_{m0j}} &
\rho(Date^{t2}\_{sd0d,exp},ID^{t3}\_{sd0j,exp})\sigma\_{Date^{t2}\_{sd0d,exp}}
\sigma\_{ID^{t3}\_{sd0j,exp}} &
\rho(Date^{t2}\_{sd0d,exp},Date^{t3}\_{m0d})\sigma\_{Date^{t2}\_{sd0d,exp}}
\sigma\_{Date^{t3}\_{m0d}} &
\rho(Date^{t2}\_{sd0d,exp},Date^{t3}\_{sd0d,exp})\sigma\_{Date^{t2}\_{sd0d,exp}}
\sigma\_{Date^{t3}\_{sd0d,exp}}\\
..... &
..... &
..... &
..... &
..... &
..... &
..... &
..... &
\sigma^2\_{ID^{t3}\_{m0}} &
\rho(ID^{t3}\_{m0j},ID^{t3}\_{sd0j,exp})\sigma\_{ID^{t3}\_{m0j}}
\sigma\_{ID^{t3}\_{sd0j,exp}} &
\rho(ID^{t3}\_{m0j},Date^{t3}\_{m0d})\sigma\_{ID^{t3}\_{m0j}}
\sigma\_{Date^{t3}\_{m0d}} &
\rho(ID^{t3}\_{m0j},Date^{t3}\_{sd0d,exp})\sigma\_{ID^{t3}\_{m0j}}
\sigma\_{Date^{t3}\_{sd0d,exp}}\\
..... &
..... &
..... &
..... &
..... &
..... &
..... &
..... &
..... &
\sigma^2\_{ID^{t3}\_{sd0,exp}} &
\rho(ID^{t3}\_{sd0j,exp},Date^{t3}\_{m0d})\sigma\_{ID^{t3}\_{sd0j,exp}}
\sigma\_{Date^{t3}\_{m0d}} &
\rho(ID^{t3}\_{sd0j,exp},Date^{t3}\_{sd0d,exp})\sigma\_{ID^{t3}\_{sd0j,exp}}
\sigma\_{Date^{t3}\_{sd0d,exp}}\\
..... &
..... &
..... &
..... &
..... &
..... &
..... &
..... &
..... &
..... &
\sigma^2\_{Date^{t3}\_{m0}} &
\rho(Date^{t3}\_{m0d},Date^{t3}\_{sd0d,exp})\sigma\_{Date^{t3}\_{m0d}}
\sigma\_{Date^{t3}\_{sd0d,exp}}\\
..... &
..... &
..... &
..... &
..... &
..... &
..... &
..... &
..... &
..... &
..... &
\sigma^2\_{Date^{t3}\_{sd0,exp}} &
\end{array}\right]
\right),\]

Based on equation 30 in O’Dea et al, 2022.

---

**FID**

```
modFID = bf(logFID 
          ~ TreatmentC + SexC + TreatmentC:SexC + DayTimeC + JumpOrderC 
                          + (1|p|PhenotypingDate) + (1|q|IndID), 
          sigma 
        ~ TreatmentC + SexC + TreatmentC:SexC +DayTimeC + JumpOrderC 
                          + (1|p|PhenotypingDate) + (1|q|IndID))
```

**Jump Distance**

```
modJumpDistance = bf(JumpDistance 
          ~ TreatmentC + SexC + TreatmentC:SexC +DayTimeC + JumpOrderC 
                          + (1|p|PhenotypingDate) + (1|q|IndID), 
          sigma 
        ~ TreatmentC + SexC + TreatmentC:SexC +DayTimeC + JumpOrderC 
                          + (1|p|PhenotypingDate) + (1|q|IndID))
```

**Jump Angle**

```
modJumpAngle = bf(JumpAngle 
          ~ TreatmentC + SexC + TreatmentC:SexC +DayTimeC + JumpOrderC 
                          + (1|p|PhenotypingDate) + (1|q|IndID), 
          sigma 
        ~ TreatmentC + SexC + TreatmentC:SexC +DayTimeC + JumpOrderC 
                          + (1|p|PhenotypingDate) + (1|q|IndID))
```

**Multivariate model**

Multivariate double hierarchical mixed effects model, with lines to
calculate the time taken to run the model.

```
#start.time = Sys.time()

#MultiMod = brm(modFID + modJumpDistance + modJumpAngle + set_rescor(TRUE), 
#               data = md, warmup = 10000,iter = 60000, thin=50, chains = 2, 
#               init = "random", seed = 12345, cores = detectCores(), 
#               control = list(adapt_delta = 0.95), sample_prior = TRUE)

#end.time = Sys.time()
#save(MultiMod, file=paste0("MultiMod_221221.RData"))
#time.taken = end.time - start.time; time.taken
```

Load model file

```
load("MultiMod_221221.RData")
```

Create a dataset with the posterior samples

```
PS=as.data.frame(MultiMod)[,1:81]
```

---

### Variance decomposition

#### Equations

**1. Variance due to fixed effects in the mean
model**

Sum of variances due to fixed effects in the mean model (variance in
the sum of slopes multiplied by design).

\[\sigma^2\_{fixed\_{m}}=var(\beta\_{m\_{1}}x\_{1j}+\beta\_{m\_{2}}x\_{2j}+\beta\_{m\_{3}}x\_{3ij}+\beta\_{m\_{4}}x\_{4ij}+\beta\_{m\_{5}}x\_{1j}x\_{2j})\]

where \(\sigma^2\_{fixed\_{m}}\) is
the variance due to fixed effects for the mean model, \(\beta\_{m\_{1}}\) is the population slope for
control-treatment contrast for the mean model, \(x\_{1j}\) is the categorical input variable
for the treatment group of individual \(j\) (\(x\_{1j}\ =
0\) for control, \(1\) for
treatment), \(\beta\_{m\_{2}}\) is the
population slope for female-male contrast for the mean model, \(x\_{2j}\) is the categorical input variable
for the treatment group of individual \(j\) (\(x\_{2j}\ =
-0.5\) for female, \(0.5\) for
male), \(\beta\_{m\_{3}}\) is the
population slope for daytime for the mean model, \(x\_{3ij}\) is the continuous input variable
for the daytime of individual \(j\) on
instance \(i\), \(\beta\_{m\_{4}}\) is the population slope for
jump order for the mean model, \(x\_{4ij}\) is the continuous input variable
for the jump order of individual \(j\)
on instance \(i\), and \(\beta\_{m\_{5}}\) is the population slope for
the interaction between the variables ‘treatment’ and ‘sex’ for the mean
model.

Based on equation 4 in O’Dea et al, 2022.

**2. Variance due to random effects in the mean
model**

\[
\sigma^2\_{random\_{m}}=\sigma^2\_{ID\_{m0}}+\sigma^2\_{date\_{m0}}\]

where \(\sigma^2\_{random\_{m}}\) is
the variance in random effects in the mean model, \(\sigma^2\_{ID\_{m0}}\) is the between
individual variance for individual intercept for the mean model, and
\(\sigma^2\_{date\_{m0}}\) is the between
phenotyping date variance for date intercept for the mean model.

**3. Variance due to fixed effects in the dispersion
model**

Sum of variances due to fixed effects in the dispersion model
(Variance in the sum of slopes multiplied by design).

\[\sigma^2\_{fixed\_{sd,exp}}=var(\beta\_{sd\_{1},exp}x\_{1j}+\beta\_{sd\_{2},exp}x\_{2j}+\beta\_{sd\_{3},exp}x\_{3ij}+\beta\_{sd\_{4},exp}x\_{4ij}
+ \beta\_{sd\_{5},exp}x\_{1j}x\_{2j})\]

where \(\sigma^2\_{fixed\_{sd,exp}}\)
is the variance due to fixed effects for the dispersion model, estimated
on the \(ln\) scale, \(\beta\_{sd\_{1},exp}\) is the population
slope for control-treatment contrast for the dispersion model, estimated
on the \(ln\) scale, \(\beta\_{sd\_{2},exp}\) is the population
slope for female-male contrast for the dispersion model, estimated on
the \(ln\) scale, \(\beta\_{sd\_{3},exp}\) is the population
slope for daytime for the dispersion model, estimated on the \(ln\) scale, \(\beta\_{sd\_{4},exp}\) is the population
slope for jump order for the dispersion model, estimated on the \(ln\) scale, and \(\beta\_{sd\_{5},exp}\) is the population
slope for the interaction between the variables ‘treatment’ and ‘sex’,
estimated in the \(ln\) scale.

Based on equation 4 in O’Dea et al, 2022.

**4. Variance due to random effects in the dispersion
model**

\[ \sigma^2\_{random\_{sd,exp}}=
\sigma^2\_{ID\_{sd0,exp}}+ \sigma^2\_{date\_{sd0,exp}} \]

where \(\sigma^2\_{random\_{sd,exp}}\)
is the variance in random effects in the dispersion model, estimated on
the \(ln\) scale, \(\sigma^2\_{ID\_{sd0,exp}}\) is the between
individual variance for individual intercept for dispersion model, on
the \(ln\) scale, and \(\sigma^2\_{date\_{sd0,exp}}\) is the between
phenotyping date variance for date intercept for dispersion model, on
the \(ln\) scale.

**5. Within individual variance**

\[\bar \sigma^2\_{w}=
exp(2\beta\_{psd0,exp}+2(\sigma^2\_{random\_{sd,exp}}+\sigma^2\_{fixed\_{sd,exp}}))\]

where \(\bar \sigma^2\_{w}\) is the
within individual variance in the observed scale, \(\beta\_{psd0,exp}\) is the population
intercept for dispersion model, estimated on the \(ln\) scale, \(\sigma^2\_{random\_{sd,exp}}\) is the
variance in random effects in the dispersion model, estimated on the
\(ln\) scale, and \(\sigma^2\_{fixed\_{sd,exp}}\) is the variance
due to fixed effects for the dispersion model, estimated on the \(ln\) scale.

Equation 56 of supplementary materials in O’Dea et al, 2022.

**6. Total phenotypic variance**

\[ \sigma^2\_{p} = \sigma^2\_{random\_{m}} +
\sigma^2\_{fixed\_{m}} + \bar\sigma^2\_{\_{w}}\]

where $ ^2\_{p}$ is the total phenotypic variance, \(\sigma^2\_{random\_{m}}\) is the variance in
random effects in the mean model, \(\sigma^2\_{fixed\_{m}}\) is the variance due
to fixed effects for the mean model and \(\bar\sigma^2\_{\_{w}}\) is the within
individual variance in the observed scale.

Based on equation 40 in O’Dea et al, 2022.

**7. Repeatabilities in the mean model**

*Individual ID*

\[R\_{p\_{ID\_{m}}}=\frac{\sigma^2\_{ID\_{m0}}}{\sigma^2\_{p}}\]

where \(R\_{p\_{ID\_{m}}}\) is the
individual repeatability in the mean model, \(\sigma^2\_{ID\_{m0}}\) is the between
individual variance for individual intercept for the mean model, and
\(\sigma^2\_{p}\) is the total
phenotypic variance.

Equation 32 in O’Dea et al, 2022.

*Phenotyping Date* \[R\_{p\_{date\_{m}}}=\frac{\sigma^2\_{date\_{m0}}}{\sigma^2\_{p}}\]

where \(R\_{p\_{date\_{m}}}\) is the
phenotyping date repeatability in the mean model, \(\sigma^2\_{date\_{m0}}\) is the between
phenotyping dates variance for date intercept for the mean model, and
\(\sigma^2\_{p}\) is the total
phenotypic variance.

Based on equation 32 in O’Dea et al, 2022.

*Residual* \[R\_{p\_{res\_{m}}}=1-(R\_{p\_{ID\_{m}}}+R\_{p\_{date\_{m}}})\]

where \(R\_{p\_{res\_{m}}}\) is the
residual in the mean model, \(R\_{p\_{ID\_{m}}}\) is the individual
repeatability in the mean model, and \(R\_{p\_{date\_{m}}}\) is the phenotyping date
repeatability in the mean model.

**8. Variance of within individual variance**

\[
\sigma^2\_{\sigma^2\_{w}}=exp(4(\sigma^2\_{random\_{sd,exp}}+\sigma^2\_{fixed\_{sd,exp}})-1)exp(4(\beta\_{sdv0,exp}+\sigma^2\_{random\_{sd,exp}}+\sigma^2\_{fixed\_{sd,exp}}))\]

where $ 2\_{\_{w}}$ is the variance of variance on the
observed scale, \(\sigma^2\_{random\_{sd,exp}}\) is the
variance in random effects in the dispersion model, estimated on the
\(ln\) scale, \(\sigma^2\_{fixed\_{sd,exp}}\) is the variance
due to fixed effects for the dispersion model, estimated on the \(ln\) scale, and \(\beta\_{sdv0,exp}\) is the population
intercept for dispersion model, estimated on the \(ln\) scale.

Based on equation 57 of supplementary materials in O’Dea et al,
2022.

**9. Variance of the total phenotypic variance**

\[\sigma^2\_{\sigma^2\_{p}}=2\sigma^4\_{p}+3\sigma^2\_{\sigma^2\_{w}}\]

where \(\sigma^2\_{\sigma^2\_{p}}\) is
the variance of total phenotypic variance, \(\sigma^2\_{p}\) is the total phenotypic
variance, and \(\sigma^2\_{\sigma^2\_{w}}\) is the variance
of variance on the observed scale.

Equation 43 in O’Dea et al, 2022.

**10. Repeatabilities in the dispersion model**

*Individual ID* First calculate between individual variance
for within individual variance in the observed scale

\[
\sigma^2\_{ID\_{v}}=\sigma^2\_{\sigma^2\_{w}}(\frac{\sigma^2\_{ID\_{sd0,exp}}}{\sigma^2\_{random\_{sd,exp}}+\sigma^2\_{fixed\_{sd,exp}}})\]

where \(\sigma^2\_{ID\_{v}}\) is the
between individual variance for the dispersion model, in the observed
scale, \(\sigma^2\_{\sigma^2\_{w}}\) is
the variance of variance on the observed scale, \(\sigma^2\_{ID\_{sd0,exp}}\) is the between
individual variance for individual intercept for dispersion model, on
the \(ln\) scale, \(\sigma^2\_{random\_{sd,exp}}\) is the
variance in random effects in the dispersion model, estimated on the
\(ln\) scale, and \(\sigma^2\_{fixed\_{sd,exp}}\) is the variance
due to fixed effects for the dispersion model, estimated on the \(ln\) scale.

Based on equation 58 of supplementary materials in O’Dea et al,
2022.

Then calculate repeatability:

\[R\_{p\_{ID\_{v}}}=\frac{\sigma^2\_{ID\_{v}}}{\sigma^2\_{\sigma^2\_{p}}}\]

where \(R\_{p\_{ID\_{v}}}\) is the
individual repeatability in the dispersion model, \(\sigma^2\_{ID\_{v}}\) is the between
individual variance for the dispersion model, in the observed scale, and
\(\sigma^2\_{\sigma^2\_{p}}\) is the
variance of total phenotypic variance.

Equation 33 in O’Dea et al, 2022.

*Phenotyping Date* First calculate between phenotyping dates
variance for within individual variance in the observed scale

\[
\sigma^2\_{date\_{v}}=\sigma^2\_{\sigma^2\_{w}}(\frac{\sigma^2\_{date\_{sd0,exp}}}{\sigma^2\_{random\_{sd,exp}}+\sigma^2\_{fixed\_{sd,exp}}})\]

where \(\sigma^2\_{date\_{v}}\) is the
between phenotyping dates variance for the dispersion model, in the
observed scale, \(\sigma^2\_{\sigma^2\_{w}}\) is the variance
of variance on the observed scale, \(\sigma^2\_{date\_{sd0,exp}}\) is the between
phenotyping dates variance for date intercept for dispersion model, on
the \(ln\) scale, \(\sigma^2\_{random\_{sd,exp}}\) is the
variance in random effects in the dispersion model, estimated on the
\(ln\) scale, and \(\sigma^2\_{fixed\_{sd,exp}}\) is the variance
due to fixed effects for the dispersion model, estimated on the \(ln\) scale.

Then calculate repeatability:

\[R\_{p\_{date\_{v}}}=\frac{\sigma^2\_{date\_{v}}}{\sigma^2\_{\sigma^2\_{p}}}\]

where \(R\_{p\_{date\_{v}}}\) is the
phenotyping date repeatability in the dispersion model, \(\sigma^2\_{date\_{v}}\) is the between
phenotyping dates variance for the dispersion model, in the observed
scale, and \(\sigma^2\_{\sigma^2\_{p}}\)
is the variance of total phenotypic variance.

Based on equation 33 in O’Dea et al, 2022.

*Residual* \[R\_{p\_{res\_{v}}}=1-(R\_{p\_{ID\_{v}}}+R\_{p\_{date\_{v}}})\]

where \(R\_{p\_{res\_{v}}}\) is the
residual in the dispersion model, \(R\_{p\_{ID\_{v}}}\) is the individual
repeatability in the dispersion model, and \(R\_{p\_{date\_{v}}}\) is the phenotyping date
repeatability in the dispersion model.

---

#### FID

**1. Variance due to fixed effects in the mean
model**

```
PS$fixed_m_logFID=0

for(i in 1:length(PS$fixed_m_logFID)){
  PS$fixed_m_logFID[i] = var(
                          (PS$b_logFID_TreatmentC[i]*md$TreatmentC) + 
                          (PS$b_logFID_SexC[i]*md$SexC) +
                          (PS$b_logFID_DayTimeC[i]*md$DayTimeC) + 
                          (PS$b_logFID_JumpOrderC[i]*md$JumpOrderC) + 
                          (PS$`b_logFID_TreatmentC:SexC`[i] 
                                                      *md$TreatmentC*md$SexC)
                          )
}
```

**2. Variance due to random effects in the mean
model**

```
PS$random_m_logFID = PS$sd_IndID__logFID_Intercept^2 +
                       PS$sd_PhenotypingDate__logFID_Intercept^2
```

**3. Variance due to fixed effects in the dispersion
model**

```
PS$fixed_sd_logFID=0

for(i in 1:length(PS$fixed_sd_logFID)){
  PS$fixed_sd_logFID[i] = var(
                          (PS$b_sigma_logFID_TreatmentC[i]*md$TreatmentC) + 
                          (PS$b_sigma_logFID_SexC[i]*md$SexC) +
                          (PS$b_sigma_logFID_DayTimeC[i]*md$DayTimeC) + 
                          (PS$b_sigma_logFID_JumpOrderC[i]*md$JumpOrderC) + 
                          (PS$`b_sigma_logFID_TreatmentC:SexC`[i]
                                                     *md$TreatmentC*md$SexC)
                          )
}
```

**4. Variance due to random effects in the dispersion
model**

```
PS$random_sd_logFID = PS$sd_IndID__sigma_logFID_Intercept^2 +
                        PS$sd_PhenotypingDate__sigma_logFID_Intercept^2
```

**5. Within individual variance**

```
PS$within_v_logFID = 
  exp((2*PS$b_sigma_logFID_Intercept) + 2*(PS$random_sd_logFID +                                                   PS$fixed_sd_logFID))
```

**6. Total phenotypic variance**

```
PS$total_pv_logFID = PS$random_m_logFID + PS$fixed_m_logFID + 
                                                        PS$within_v_logFID
```

**7. Repeatabilities in the mean model**

*Individual ID*

```
PS$Rp_IndID_m_logFID = PS$sd_IndID__logFID_Intercept^2 / PS$total_pv_logFID

median(PS$Rp_IndID_m_logFID); HPDinterval(as.mcmc(PS$Rp_IndID_m_logFID),0.95)
```

```
## [1] 0.07003669
```

```
##           lower    upper
## var1 0.02788033 0.120908
## attr(,"Probability")
## [1] 0.95
```

*Phenotyping Date*

```
PS$Rp_PhenotypingDate_m_logFID = PS$sd_PhenotypingDate__logFID_Intercept^2 / 
                                                          PS$total_pv_logFID

median(PS$Rp_PhenotypingDate_m_logFID);
```

```
## [1] 0.3287168
```

```
HPDinterval(as.mcmc(PS$Rp_PhenotypingDate_m_logFID),0.95)
```

```
##          lower     upper
## var1 0.1301163 0.6132458
## attr(,"Probability")
## [1] 0.95
```

*Residual*

```
PS$Rp_Res_m_logFID = 1 -( PS$Rp_IndID_m_logFID + 
                                 PS$Rp_PhenotypingDate_m_logFID )

median(PS$Rp_Res_m_logFID);
```

```
## [1] 0.5972007
```

```
HPDinterval(as.mcmc(PS$Rp_Res_m_logFID),0.95)
```

```
##          lower     upper
## var1 0.3491431 0.7964295
## attr(,"Probability")
## [1] 0.95
```

**8. Variance of within individual variance**

```
PS$v_within_v_logFID=(exp(4*(PS$fixed_sd_logFID + PS$random_sd_logFID)) 
                           -1) * (exp ((4*(PS$b_sigma_logFID_Intercept + 
                                             PS$fixed_sd_logFID + 
                                             PS$random_sd_logFID))))
```

**9. Variance of the total phenotypic variance**

```
PS$v_total_pv_logFID= (2*(PS$total_pv_logFID^2)) + 
                                      (3*PS$v_within_v_logFID)
```

**10. Repeatabilities in the dispersion model**

*Individual ID*

```
PS$v_IndID_v_logFID = PS$v_within_v_logFID * 
                               ((PS$sd_IndID__sigma_logFID_Intercept^2)/
                               (PS$random_sd_logFID + PS$fixed_sd_logFID))


PS$Rp_IndID_v_logFID = PS$v_IndID_v_logFID / 
                                              PS$v_total_pv_logFID

median(PS$Rp_IndID_v_logFID); HPDinterval(as.mcmc(PS$Rp_IndID_v_logFID),0.95)
```

```
## [1] 0.04702628
```

```
##           lower      upper
## var1 0.01145173 0.09396906
## attr(,"Probability")
## [1] 0.95
```

*Phenotyping Date*

```
PS$v_PhenotypingDate_v_logFID = PS$v_within_v_logFID *
                        ((PS$sd_PhenotypingDate__sigma_logFID_Intercept^2)/
                              (PS$random_sd_logFID + PS$fixed_sd_logFID))

PS$Rp_PhenotypingDate_v_logFID = PS$v_PhenotypingDate_v_logFID /
                                    PS$v_total_pv_logFID

median(PS$Rp_PhenotypingDate_v_logFID);
```

```
## [1] 0.009262551
```

```
HPDinterval(as.mcmc(PS$Rp_PhenotypingDate_v_logFID),0.95)
```

```
##                  lower      upper
## var1 0.000000001266471 0.05709211
## attr(,"Probability")
## [1] 0.95
```

*Residual*

```
PS$Rp_Res_v_logFID = 1 -( PS$Rp_IndID_v_logFID + 
                                   PS$Rp_PhenotypingDate_v_logFID)

median(PS$Rp_Res_v_logFID);
```

```
## [1] 0.9383308
```

```
HPDinterval(as.mcmc(PS$Rp_Res_v_logFID),0.95)
```

```
##          lower     upper
## var1 0.8690191 0.9831775
## attr(,"Probability")
## [1] 0.95
```

---

#### Jump Distance

**1. Variance due to fixed effects in the mean
model**

```
PS$fixed_m_JumpDistance=0

for(i in 1:length(PS$fixed_m_JumpDistance)){
  PS$fixed_m_JumpDistance[i] = var(
                          (PS$b_JumpDistance_TreatmentC[i]*md$TreatmentC) + 
                          (PS$b_JumpDistance_SexC[i]*md$SexC) +
                          (PS$b_JumpDistance_DayTimeC[i]*md$DayTimeC) + 
                          (PS$b_JumpDistance_JumpOrderC[i]*md$JumpOrderC) + 
                          (PS$`b_JumpDistance_TreatmentC:SexC`[i] 
                                                      *md$TreatmentC*md$SexC)
                          )
}
```

**2. Variance due to random effects in the mean
model**

```
PS$random_m_JumpDistance = PS$sd_IndID__JumpDistance_Intercept^2 +
                       PS$sd_PhenotypingDate__JumpDistance_Intercept^2
```

**3. Variance due to fixed effects in the dispersion
model**

```
PS$fixed_sd_JumpDistance=0

for(i in 1:length(PS$fixed_sd_JumpDistance)){
  PS$fixed_sd_JumpDistance[i] = (var(
                          (PS$b_sigma_JumpDistance_TreatmentC[i]*md$TreatmentC) + 
                          (PS$b_sigma_JumpDistance_SexC[i]*md$SexC) +
                          (PS$b_sigma_JumpDistance_DayTimeC[i]*md$DayTimeC) + 
                          (PS$b_sigma_JumpDistance_JumpOrderC[i]*md$JumpOrderC) + 
                          (PS$`b_sigma_JumpDistance_TreatmentC:SexC`[i]
                                                     *md$TreatmentC*md$SexC)
                          ))
}
```

**4. Variance due to random effects in the dispersion
model**

```
PS$random_sd_JumpDistance = PS$sd_IndID__sigma_JumpDistance_Intercept^2 +
                        PS$sd_PhenotypingDate__sigma_JumpDistance_Intercept^2
```

**5. Within individual variance**

```
PS$within_v_JumpDistance = 
  exp((2*PS$b_sigma_JumpDistance_Intercept) + 2*(PS$random_sd_JumpDistance + 
                                                      PS$fixed_sd_JumpDistance))
```

**6. Total phenotypic variance**

```
PS$total_pv_JumpDistance = PS$random_m_JumpDistance + PS$fixed_m_JumpDistance + 
                                                        PS$within_v_JumpDistance
```

**7. Repeatabilities in the mean model**

*Individual ID*

```
PS$Rp_IndID_m_JumpDistance = PS$sd_IndID__JumpDistance_Intercept^2 / PS$total_pv_JumpDistance

median(PS$Rp_IndID_m_JumpDistance); HPDinterval(as.mcmc(PS$Rp_IndID_m_JumpDistance),0.95)
```

```
## [1] 0.2767868
```

```
##          lower     upper
## var1 0.1822195 0.3747933
## attr(,"Probability")
## [1] 0.95
```

*Phenotyping Date*

```
PS$Rp_PhenotypingDate_m_JumpDistance = PS$sd_PhenotypingDate__JumpDistance_Intercept^2 / 
                                                          PS$total_pv_JumpDistance

median(PS$Rp_PhenotypingDate_m_JumpDistance);
```

```
## [1] 0.09238505
```

```
HPDinterval(as.mcmc(PS$Rp_PhenotypingDate_m_JumpDistance),0.95)
```

```
##              lower     upper
## var1 0.00000386966 0.2631651
## attr(,"Probability")
## [1] 0.95
```

*Residual*

```
PS$Rp_Res_m_JumpDistance = 1 -( PS$Rp_IndID_m_JumpDistance + 
                                 PS$Rp_PhenotypingDate_m_JumpDistance )

median(PS$Rp_Res_m_JumpDistance);
```

```
## [1] 0.6182533
```

```
HPDinterval(as.mcmc(PS$Rp_Res_m_JumpDistance),0.95)
```

```
##          lower     upper
## var1 0.4786198 0.7283232
## attr(,"Probability")
## [1] 0.95
```

**8. Variance of within individual variance**

```
PS$v_within_v_JumpDistance=(exp(4*(PS$fixed_sd_JumpDistance + PS$random_sd_JumpDistance)) 
                           -1) * (exp ((4*(PS$b_sigma_JumpDistance_Intercept + 
                                             PS$fixed_sd_JumpDistance + 
                                             PS$random_sd_JumpDistance))))
```

**9. Variance of the total phenotypic variance**

```
PS$v_total_pv_JumpDistance= (2*(PS$total_pv_JumpDistance^2)) + 
                                      (3*PS$v_within_v_JumpDistance)
```

**10. Repeatabilities in the dispersion model**

*Individual ID*

```
PS$v_IndID_v_JumpDistance = PS$v_within_v_JumpDistance * 
                               ((PS$sd_IndID__sigma_JumpDistance_Intercept^2)/
                               (PS$random_sd_JumpDistance + PS$fixed_sd_JumpDistance))


PS$Rp_IndID_v_JumpDistance = PS$v_IndID_v_JumpDistance / 
                                              PS$v_total_pv_JumpDistance

median(PS$Rp_IndID_v_JumpDistance); HPDinterval(as.mcmc(PS$Rp_IndID_v_JumpDistance),0.95)
```

```
## [1] 0.0285729
```

```
##            lower      upper
## var1 0.008286083 0.05479567
## attr(,"Probability")
## [1] 0.95
```

*Phenotyping Date*

```
PS$v_PhenotypingDate_v_JumpDistance = PS$v_within_v_JumpDistance *
                        ((PS$sd_PhenotypingDate__sigma_JumpDistance_Intercept^2)/
                              (PS$random_sd_JumpDistance + PS$fixed_sd_JumpDistance))

PS$Rp_PhenotypingDate_v_JumpDistance = PS$v_PhenotypingDate_v_JumpDistance /
                                    PS$v_total_pv_JumpDistance

median(PS$Rp_PhenotypingDate_v_JumpDistance);
```

```
## [1] 0.004457932
```

```
HPDinterval(as.mcmc(PS$Rp_PhenotypingDate_v_JumpDistance),0.95)
```

```
##                  lower      upper
## var1 0.000000002155855 0.03163259
## attr(,"Probability")
## [1] 0.95
```

*Residual*

```
PS$Rp_Res_v_JumpDistance = 1 -( PS$Rp_IndID_v_JumpDistance + 
                                   PS$Rp_PhenotypingDate_v_JumpDistance)

median(PS$Rp_Res_v_JumpDistance);
```

```
## [1] 0.9645167
```

```
HPDinterval(as.mcmc(PS$Rp_Res_v_JumpDistance),0.95)
```

```
##          lower     upper
## var1 0.9264292 0.9907947
## attr(,"Probability")
## [1] 0.95
```

---

#### Jump Angle

**1. Variance due to fixed effects in the mean
model**

```
PS$fixed_m_JumpAngle=0

for(i in 1:length(PS$fixed_m_JumpAngle)){
  PS$fixed_m_JumpAngle[i] = var(
                          (PS$b_JumpAngle_TreatmentC[i]*md$TreatmentC) + 
                          (PS$b_JumpAngle_SexC[i]*md$SexC) +
                          (PS$b_JumpAngle_DayTimeC[i]*md$DayTimeC) + 
                          (PS$b_JumpAngle_JumpOrderC[i]*md$JumpOrderC) + 
                          (PS$`b_JumpAngle_TreatmentC:SexC`[i] 
                                                      *md$TreatmentC*md$SexC)
                          )
}
```

**2. Variance due to random effects in the mean
model**

```
PS$random_m_JumpAngle = PS$sd_IndID__JumpAngle_Intercept^2 +
                       PS$sd_PhenotypingDate__JumpAngle_Intercept^2
```

**3. Variance due to fixed effects in the dispersion
model**

```
PS$fixed_sd_JumpAngle=0

for(i in 1:length(PS$fixed_sd_JumpAngle)){
  PS$fixed_sd_JumpAngle[i] = (var(
                          (PS$b_sigma_JumpAngle_TreatmentC[i]*md$TreatmentC) + 
                          (PS$b_sigma_JumpAngle_SexC[i]*md$SexC) +
                          (PS$b_sigma_JumpAngle_DayTimeC[i]*md$DayTimeC) + 
                          (PS$b_sigma_JumpAngle_JumpOrderC[i]*md$JumpOrderC) + 
                          (PS$`b_sigma_JumpAngle_TreatmentC:SexC`[i]
                                                     *md$TreatmentC*md$SexC)
                          ))
}
```

**4. Variance due to random effects in the dispersion
model**

```
PS$random_sd_JumpAngle = PS$sd_IndID__sigma_JumpAngle_Intercept^2 +
                        PS$sd_PhenotypingDate__sigma_JumpAngle_Intercept^2
```

**5. Within individual variance**

```
PS$within_v_JumpAngle = 
  exp((2*PS$b_sigma_JumpAngle_Intercept) + 2*(PS$random_sd_JumpAngle + 
                                                      PS$fixed_sd_JumpAngle))
```

**6. Total phenotypic variance**

```
PS$total_pv_JumpAngle = PS$random_m_JumpAngle + PS$fixed_m_JumpAngle + 
                                                        PS$within_v_JumpAngle
```

**7. Repeatabilities in the mean model**

*Individual ID*

```
PS$Rp_IndID_m_JumpAngle = PS$sd_IndID__JumpAngle_Intercept^2 / PS$total_pv_JumpAngle

median(PS$Rp_IndID_m_JumpAngle); HPDinterval(as.mcmc(PS$Rp_IndID_m_JumpAngle),0.95)
```

```
## [1] 0.1066656
```

```
##           lower     upper
## var1 0.05079086 0.1723614
## attr(,"Probability")
## [1] 0.95
```

*Phenotyping Date*

```
PS$Rp_PhenotypingDate_m_JumpAngle = PS$sd_PhenotypingDate__JumpAngle_Intercept^2 / 
                                                          PS$total_pv_JumpAngle

median(PS$Rp_PhenotypingDate_m_JumpAngle);
```

```
## [1] 0.01694691
```

```
HPDinterval(as.mcmc(PS$Rp_PhenotypingDate_m_JumpAngle),0.95)
```

```
##                lower      upper
## var1 0.0000001942283 0.08311293
## attr(,"Probability")
## [1] 0.95
```

*Residual*

```
PS$Rp_Res_m_JumpAngle = 1 -( PS$Rp_IndID_m_JumpAngle + 
                                 PS$Rp_PhenotypingDate_m_JumpAngle )

median(PS$Rp_Res_m_JumpAngle);
```

```
## [1] 0.8692622
```

```
HPDinterval(as.mcmc(PS$Rp_Res_m_JumpAngle),0.95)
```

```
##         lower     upper
## var1 0.778606 0.9332243
## attr(,"Probability")
## [1] 0.95
```

**8. Variance of within individual variance**

```
PS$v_within_v_JumpAngle=(exp(4*(PS$fixed_sd_JumpAngle + PS$random_sd_JumpAngle)) 
                           -1) * (exp ((4*(PS$b_sigma_JumpAngle_Intercept + 
                                             PS$fixed_sd_JumpAngle + 
                                             PS$random_sd_JumpAngle))))
```

**9. Variance of the total phenotypic variance**

```
PS$v_total_pv_JumpAngle= (2*(PS$total_pv_JumpAngle^2)) + 
                                      (3*PS$v_within_v_JumpAngle)
```

**10. Repeatabilities in the dispersion model**

*Individual ID*

```
PS$v_IndID_v_JumpAngle = PS$v_within_v_JumpAngle * 
                               ((PS$sd_IndID__sigma_JumpAngle_Intercept^2)/
                               (PS$random_sd_JumpAngle + PS$fixed_sd_JumpAngle))

PS$Rp_IndID_v_JumpAngle = PS$v_IndID_v_JumpAngle / 
                                              PS$v_total_pv_JumpAngle

median(PS$Rp_IndID_v_JumpAngle); HPDinterval(as.mcmc(PS$Rp_IndID_v_JumpAngle),0.95)
```

```
## [1] 0.07191078
```

```
##           lower     upper
## var1 0.03449153 0.1150014
## attr(,"Probability")
## [1] 0.95
```

*Phenotyping Date*

```
PS$v_PhenotypingDate_v_JumpAngle = PS$v_within_v_JumpAngle *
                        ((PS$sd_PhenotypingDate__sigma_JumpAngle_Intercept^2)/
                              (PS$random_sd_JumpAngle + PS$fixed_sd_JumpAngle))

PS$Rp_PhenotypingDate_v_JumpAngle = PS$v_PhenotypingDate_v_JumpAngle /
                                    PS$v_total_pv_JumpAngle

median(PS$Rp_PhenotypingDate_v_JumpAngle);
```

```
## [1] 0.01425965
```

```
HPDinterval(as.mcmc(PS$Rp_PhenotypingDate_v_JumpAngle),0.95)
```

```
##               lower      upper
## var1 0.000000302043 0.07585896
## attr(,"Probability")
## [1] 0.95
```

*Residual*

```
PS$Rp_Res_v_JumpAngle = 1 -( PS$Rp_IndID_v_JumpAngle + 
                                   PS$Rp_PhenotypingDate_v_JumpAngle)

median(PS$Rp_Res_v_JumpAngle);
```

```
## [1] 0.9070612
```

```
HPDinterval(as.mcmc(PS$Rp_Res_v_JumpAngle),0.95)
```

```
##          lower     upper
## var1 0.8495031 0.9540897
## attr(,"Probability")
## [1] 0.95
```

---

### Coefficient of individual variation

#### Equations

**Coefficient of individual variation for the mean
model**

\[CV\_{ID\_{m}}=\frac{\sigma\_{ID\_{m}}}{\beta\_{pm0}}\]
where \(CV\_{ID\_{m}}\) is the
coefficient of individual variation for the mean model, \(\sigma\_{ID\_{m}}\) is the among individual
standard deviation for the mean model and \(\beta\_{pm0}\) is the population intercept
for the mean model.

Based on equation 34 in O’Dea et al, 2022.

**Coefficient of individual variation for the mean
model**

\[CV\_{ID\_{v}}=\frac{\sigma\_{ID\_{v}}}{\bar{\sigma}^2\_{w}}\]

where \(CV\_{ID\_{v}}\) is the
coefficient of individual variation in the dispersion model, estimated
on the observed scale, \(\sigma\_{ID\_{v}}\) is the between individual
standard deviation for the dispersion model, in the observed scale, and
\(\bar \sigma^2\_{w}\) is the within
individual variance in the observed scale.

Based on equation 55 in the supplementary material of O’Dea et al,
2022.

---

#### FID

**Coefficient of individual variation for the mean
model**

```
PS$CV_logFID_m = PS$sd_IndID__logFID_Intercept / PS$b_logFID_Intercept

median(PS$CV_logFID_m);
```

```
## [1] 0.04367235
```

```
HPDinterval(as.mcmc(PS$CV_logFID_m),0.95)
```

```
##           lower      upper
## var1 0.03299228 0.05775245
## attr(,"Probability")
## [1] 0.95
```

**Coefficient of individual variation for the dispersion
model**

```
PS$CV_logFID_v = (sqrt(PS$v_IndID_v_logFID))/PS$within_v_logFID 

median(PS$CV_logFID_v);
```

```
## [1] 0.6241557
```

```
HPDinterval(as.mcmc(PS$CV_logFID_v),0.95)
```

```
##         lower     upper
## var1 0.421587 0.8438313
## attr(,"Probability")
## [1] 0.95
```

---

#### Jump Distance

**Coefficient of individual variation for the mean
model**

```
PS$CV_JumpDistance_m = PS$sd_IndID__JumpDistance_Intercept / PS$b_JumpDistance_Intercept

median(PS$CV_JumpDistance_m);
```

```
## [1] 0.2188579
```

```
HPDinterval(as.mcmc(PS$CV_JumpDistance_m),0.95)
```

```
##          lower     upper
## var1 0.1754361 0.2607968
## attr(,"Probability")
## [1] 0.95
```

**Coefficient of individual variation for the dispersion
model**

```
PS$CV_JumpDistance_v = (sqrt(PS$v_IndID_v_JumpDistance))/PS$within_v_JumpDistance 

median(PS$CV_JumpDistance_v);
```

```
## [1] 0.5105617
```

```
HPDinterval(as.mcmc(PS$CV_JumpDistance_v),0.95)
```

```
##          lower     upper
## var1 0.3345121 0.7345176
## attr(,"Probability")
## [1] 0.95
```

---

#### Jump Angle

**Coefficient of individual variation for the mean
model**

```
PS$CV_JumpAngle_m = PS$sd_IndID__JumpAngle_Intercept / PS$b_JumpAngle_Intercept

median(PS$CV_JumpAngle_m);
```

```
## [1] -3.120588
```

```
HPDinterval(as.mcmc(PS$CV_JumpAngle_m),0.95)
```

```
##          lower    upper
## var1 -43.38058 45.06265
## attr(,"Probability")
## [1] 0.95
```

**Coefficient of individual variation for the dispersion
model**

```
PS$CV_JumpAngle_v = (sqrt(PS$v_IndID_v_JumpAngle))/PS$within_v_JumpAngle 

median(PS$CV_JumpAngle_v);
```

```
## [1] 0.5452721
```

```
HPDinterval(as.mcmc(PS$CV_JumpAngle_v),0.95)
```

```
##          lower     upper
## var1 0.3501548 0.7336086
## attr(,"Probability")
## [1] 0.95
```

### Results

**Posterior samples summaries**

```
EstMultiMod = summarise_draws(PS) %>% 
  select(variable, median, sd, rhat, ess_bulk) 
  
HPDMultiMod = as.data.frame(HPDinterval(as.mcmc(PS, combine_chains = TRUE))) %>% 
  rename(lower_HPD = lower, upper_HPD = upper) %>% 
  rownames_to_column(var = "variable")

EstMultiMod = left_join(EstMultiMod, HPDMultiMod, by = "variable")
```

**Fixed effects (b), random effects (Rp) and coefficient of
individual variation (CV)**

```
EstModel = EstMultiMod %>% 
  filter(str_detect(variable, "b_|CV_|Rp_")) %>% 
   mutate(variable = str_replace(variable,"TreatmentC", "Treatment"),
          variable = str_replace(variable,"SexC", "Sex"),
          variable = str_replace(variable,"DayTimeC", "Daytime"),
          variable = str_replace(variable,"JumpOrderC", "JumpOrder"),
          variable = str_replace(variable,"Treatment:Sex", "Treatment:Sex"),
     variable = str_replace(variable,"b_logFID", "b_average_logFID"),
          variable = str_replace(variable,"b_JumpDistance","b_average_JumpDistance"),
          variable = str_replace(variable,"b_JumpAngle", "b_average_JumpAngle"),
          variable = str_replace(variable,"CV_logFID_m", "CV_average_logFID_IndID"),
          variable = str_replace(variable,"CV_JumpDistance_m", "CV_average_JumpDistance_IndID"),
          variable = str_replace(variable,"CV_JumpAngle_m", "CV_average_JumpAngle_IndID"),
          variable = str_replace(variable,"CV_logFID_v", "CV_sigma_logFID_IndID"),
          variable = str_replace(variable,"CV_JumpDistance_v", "CV_sigma_JumpDistance_IndID"),
          variable = str_replace(variable,"CV_JumpAngle_v", "CV_sigma_JumpAngle_IndID"),
          variable = str_replace(variable,"Rp_IndID_m_logFID", "R2_average_logFID_IndID"),
          variable = str_replace(variable,"Rp_IndID_m_JumpDistance", "R2_average_JumpDistance_IndID"),
          variable = str_replace(variable,"Rp_IndID_m_JumpAngle", "R2_average_JumpAngle_IndID"),
          variable = str_replace(variable,"Rp_IndID_v_logFID", "R2_sigma_logFID_IndID"),
          variable = str_replace(variable,"Rp_IndID_v_JumpDistance", "R2_sigma_JumpDistance_IndID"),
          variable = str_replace(variable,"Rp_IndID_v_JumpAngle", "R2_sigma_JumpAngle_IndID"),
          variable = str_replace(variable,"Rp_PhenotypingDate_m_logFID", "R2_average_logFID_Date"),
          variable = str_replace(variable,"Rp_PhenotypingDate_m_JumpDistance", "R2_average_JumpDistance_Date"),
          variable = str_replace(variable,"Rp_PhenotypingDate_m_JumpAngle", "R2_average_JumpAngle_Date"),
          variable = str_replace(variable,"Rp_PhenotypingDate_v_logFID", "R2_sigma_logFID_Date"),
          variable = str_replace(variable,"Rp_PhenotypingDate_v_JumpDistance", "R2_sigma_JumpDistance_Date"),
          variable = str_replace(variable,"Rp_PhenotypingDate_v_JumpAngle", "R2_sigma_JumpAngle_Date"),
          variable = str_replace(variable,"Rp_Res_m_logFID", "R2_average_logFID_Residual"),
          variable = str_replace(variable,"Rp_Res_m_JumpDistance", "R2_average_JumpDistance_Residual"),
          variable = str_replace(variable,"Rp_Res_m_JumpAngle", "R2_average_JumpAngle_Residual"),
          variable = str_replace(variable,"Rp_Res_v_logFID", "R2_sigma_logFID_Residual"),
          variable = str_replace(variable,"Rp_Res_v_JumpDistance", "R2_sigma_JumpDistance_Residual"),
          variable = str_replace(variable,"Rp_Res_v_JumpAngle", "R2_sigma_JumpAngle_Residual")) %>%  
  separate(col = variable,into = c("coefficient", "variable","trait","parameter"), sep="_") %>% 
  arrange(trait, variable);EstModel
```

**Correlations**

```
EstCorrelations = EstMultiMod %>% 
  filter(str_detect(variable, "cor_"))%>% 
   mutate(variable = str_replace(variable,"__sigma_logFID_Intercept", "__sigma_logFID"),
          variable = str_replace(variable,"__sigma_JumpDistance_Intercept", "__sigma_JumpDistance"),
          variable = str_replace(variable,"__sigma_JumpAngle_Intercept", "__sigma_JumpAngle"),
          variable = str_replace(variable,"__sigma_logFID_Intercept", "__sigma_logFID"),
          variable = str_replace(variable,"__sigma_JumpDistance_Intercept", "__sigma_JumpDistance"),
          variable = str_replace(variable,"__sigma_JumpAngle_Intercept", "__sigma_JumpAngle"),
          variable = str_replace(variable,"__logFID_Intercept", "__average_logFID"),
          variable = str_replace(variable,"__JumpDistance_Intercept", "__average_JumpDistance"),
          variable = str_replace(variable,"__JumpAngle_Intercept", "__average_JumpAngle"),
          variable = str_replace(variable,"__logFID_Intercept", "__average_logFID"),
          variable = str_replace(variable,"__JumpDistance_Intercept", "__average_JumpDistance"),
          variable = str_replace(variable,"__JumpAngle_Intercept", "__average_JumpAngle"),
          variable = str_replace(variable,"rescor_", "Residual_"),
          variable = str_replace(variable,"cor_","")) %>% 
  separate(col = variable,into = c("RandomEffect", "Trait 1","Trait 2"), sep="__");EstCorrelations
```

---

### Model fit checks

#### Gelman-Rubin convergence criteria

Maximum \(\hat{r message=FALSE,
warning=FALSE}\) value

```
max(EstMultiMod$rhat)
```

```
## [1] 1.004948
```

```
mcmc_plot(MultiMod, type = "rhat")
```

---

#### Effective sample size

Minimum effective sample size

```
min(EstMultiMod$ess_bulk)
```

```
## [1] 1528.44
```

```
mcmc_plot(MultiMod, type="neff")
```

---

#### Trace plots

Trace plots for checking convergence

##### FID

```
mcmc_trace(as_draws_df(MultiMod), 
           pars = vars(contains("b_logFID"), contains("sd_IndID__logFID"), contains("sd_PhenotypingDate__logFID"),contains("b_sigma_logFID"), contains("sd_IndID__sigma_logFID"), contains("sd_PhenotypingDate__sigma_logFID"),-contains("prior")),
           np = nuts_params(MultiMod),
           facet_args = list(ncol = 4), 
           size = 0.15) +
    labs(title = "Flight initiation distance")+
  theme(text = element_text(size = 10), legend.position = "bottom")
```

##### Jump Distance

```
mcmc_trace(as_draws_df(MultiMod), 
           pars = vars(contains("b_JumpDistance"), contains("sd_IndID__JumpDistance"), contains("sd_PhenotypingDate__JumpDistance"),contains("b_sigma_JumpDistance"), contains("sd_IndID__sigma_JumpDistance"), contains("sd_PhenotypingDate__sigma_JumpDistance"),-contains("prior")),
           np = nuts_params(MultiMod),
           facet_args = list(ncol = 4), 
           size = 0.15) +
  labs(title = "Jump distance")+
  theme(text = element_text(size = 10), legend.position = "bottom")
```

##### Jump Angle

```
mcmc_trace(as_draws_df(MultiMod), 
           pars = vars(contains("b_JumpAngle"), contains("sd_IndID__JumpAngle"), contains("sd_PhenotypingDate__JumpAngle"),contains("b_sigma_JumpAngle"), contains("sd_IndID__sigma_JumpAngle"), contains("sd_PhenotypingDate__sigma_JumpAngle"),-contains("prior")),
           np = nuts_params(MultiMod),
           facet_args = list(ncol = 4), 
           size = 0.15) +
    labs(title = "Jump Angle")+
  theme(text = element_text(size = 10), legend.position = "bottom")
```

---

#### Posterior predictive checks

##### FID

```
pp_check(MultiMod, resp = "logFID", type = "dens_overlay", ndraws = 30)
```

```
pp_check(MultiMod, resp = "logFID", type = "error_scatter_avg")
```

```
pp_check(MultiMod, resp = "logFID", type = "intervals")
```

```
pp_check(MultiMod, resp = "logFID", type = "loo_pit_qq")
```

```
pp_check(MultiMod, resp = "logFID", type = "stat_2d")
```

```
pp_check(MultiMod, resp = "logFID", type = "violin_grouped", 
          group= "TreatmentC")
```

---

##### Jump Distance

```
pp_check(MultiMod, resp = "JumpDistance", type = "dens_overlay", ndraws = 30)
```

```
pp_check(MultiMod, resp = "JumpDistance", type = "error_scatter_avg")
```

```
pp_check(MultiMod, resp = "JumpDistance", type = "intervals")
```

```
pp_check(MultiMod, resp = "JumpDistance", type = "loo_pit")
```

```
pp_check(MultiMod, resp = "JumpDistance", type = "stat_2d")
```

```
pp_check(MultiMod, resp = "JumpDistance", type = "violin_grouped", 
          group= "TreatmentC")
```

---

##### Jump Angle

```
pp_check(MultiMod, resp = "JumpAngle", type = "dens_overlay", ndraws = 30)
```

```
pp_check(MultiMod, resp = "JumpAngle", type = "error_scatter_avg")
```

```
pp_check(MultiMod, resp = "JumpAngle", type = "intervals")
```

```
pp_check(MultiMod, resp = "JumpAngle", type = "loo_pit")
```

```
pp_check(MultiMod, resp = "JumpAngle", type = "stat_2d")
```

```
pp_check(MultiMod, resp = "JumpAngle", type = "violin_grouped", 
          group= "TreatmentC")
```

---

#### Prior samples

Checking the influence of brms default priors on the posterior
samples.

```
priordraws <- prior_draws(MultiMod)

priordrawslong = select(priordraws, starts_with("sd")) %>%
  pivot_longer(cols = 1:12, names_to = "variable", values_to = "value")%>%
  add_column(name = "prior")

PSlong = select(PS, starts_with("sd")) %>%
  pivot_longer(cols = 1:12, names_to = "variable", values_to = "value") %>%
  add_column(name = "posterior")

priorposterior= rbind.fill(priordrawslong,PSlong)%>%
  unite("variable", variable,name, remove = FALSE)
```

##### FID

**Average behavior**

*Individual ID*

```
IndID_logFID_m_prior_log = priorposterior %>%
  filter(str_detect(variable, "IndID__logFID")) %>%
  mutate(value=log(value))
  
ggplot(data=IndID_logFID_m_prior_log, aes(x=value, fill = name))+
  geom_density(color=NA, alpha=0.6)+
  scale_fill_manual(values=c("#219EBC","#8ECAE6"))+ 
 labs(x="Individual ID standard deviation in flight initiation distance  (log)",y="Density")+
  theme_test()
```

*Phenotyping date*

```
PhenotypingDate_logFID_m_prior_log = priorposterior %>%
  filter(str_detect(variable, "PhenotypingDate__logFID")) %>%
  mutate(value=log(value))
  
ggplot(data=PhenotypingDate_logFID_m_prior_log, aes(x=value, fill = name))+
  geom_density(color=NA, alpha=0.6)+
  scale_fill_manual(values=c("#219EBC","#8ECAE6"))+ 
 labs(x="Phenotyping date standard deviation in flight initiation distance  (log)",y="Density")+
  theme_test()
```

**(Un)predictability**

*Individual ID*

```
IndID_JumpDistance_v_prior_log = priorposterior %>%
  filter(str_detect(variable, "IndID__sigma_JumpDistance")) %>%
  mutate(value=log(value))
  
ggplot(data=IndID_JumpDistance_v_prior_log, aes(x=value, fill = name))+
  geom_density(color=NA, alpha=0.6)+
  scale_fill_manual(values=c("#219EBC","#8ECAE6"))+ 
 labs(x="Individual ID standard deviation in flight initiation distance (un)predictability (log)",y="Density")+
  theme_test()
```

*Phenotyping date*

```
PhenotypingDate_JumpDistance_v_prior_log = priorposterior %>%
  filter(str_detect(variable, "PhenotypingDate__sigma_JumpDistance"))%>%
  mutate(value=log(value))
  
ggplot(data=PhenotypingDate_JumpDistance_v_prior_log, aes(x=value, fill = name))+
  geom_density(color=NA, alpha=0.6)+
  scale_fill_manual(values=c("#219EBC","#8ECAE6"))+ 
 labs(x="Phenotyping date standard deviation in flight initiation distance (un)predictability (log)",y="Density")+
  theme_test()
```

---

##### Jump Distance

**Average behavior**

*Individual ID*

```
IndID_JumpDistance_m_prior_log = priorposterior %>%
  filter(str_detect(variable, "IndID__JumpDistance")) %>%
  mutate(value=log(value))
  
ggplot(data=IndID_JumpDistance_m_prior_log, aes(x=value, fill = name))+
  geom_density(color=NA, alpha=0.6)+
  scale_fill_manual(values=c("#219EBC","#8ECAE6"))+ 
 labs(x="Individual ID standard deviation in jump distance  (log)",y="Density")+
  theme_test()
```

*Phenotyping date*

```
PhenotypingDate_JumpDistance_m_prior_log = priorposterior %>%
  filter(str_detect(variable, "PhenotypingDate__JumpDistance")) %>%
  mutate(value=log(value))
  
ggplot(data=PhenotypingDate_JumpDistance_m_prior_log, aes(x=value, fill = name))+
  geom_density(color=NA, alpha=0.6)+
  scale_fill_manual(values=c("#219EBC","#8ECAE6"))+ 
 labs(x="Phenotyping date standard deviation in jump distance  (log)",y="Density")+
  theme_test()
```

**(Un)predictability**

*Individual ID*

```
IndID_JumpDistance_v_prior_log = priorposterior %>%
  filter(str_detect(variable, "IndID__sigma_JumpDistance")) %>%
  mutate(value=log(value))
  
ggplot(data=IndID_JumpDistance_v_prior_log, aes(x=value, fill = name))+
  geom_density(color=NA, alpha=0.6)+
  scale_fill_manual(values=c("#219EBC","#8ECAE6"))+ 
 labs(x="Individual ID standard deviation in jump distance (un)predictability (log)",y="Density")+
  theme_test()
```

*Phenotyping date*

```
PhenotypingDate_JumpDistance_v_prior_log = priorposterior %>%
  filter(str_detect(variable, "PhenotypingDate__sigma_JumpDistance")) %>%
  mutate(value=log(value))
  
ggplot(data=PhenotypingDate_JumpDistance_v_prior_log, aes(x=value, fill = name))+
  geom_density(color=NA, alpha=0.6)+
  scale_fill_manual(values=c("#219EBC","#8ECAE6"))+ 
 labs(x="Phenotyping date standard deviation in jump distance (un)predictability (log)",y="Density")+
  theme_test()
```

---

##### Jump Angle

**Average behavior**

*Individual ID*

```
IndID_JumpAngle_m_prior_log = priorposterior %>%
  filter(str_detect(variable, "IndID__JumpAngle")) %>%
  mutate(value=log(value))
  
ggplot(data=IndID_JumpAngle_m_prior_log, aes(x=value, fill = name))+
  geom_density(color=NA, alpha=0.6)+
  scale_fill_manual(values=c("#219EBC","#8ECAE6"))+ 
 labs(x="Individual ID standard deviation in jump angle  (log)",y="Density")+
  theme_test()
```

*Phenotyping date*

```
PhenotypingDate_JumpAngle_m_prior_log = priorposterior %>%
  filter(str_detect(variable, "PhenotypingDate__JumpAngle")) %>%
  mutate(value=log(value))
  
ggplot(data=PhenotypingDate_JumpAngle_m_prior_log, aes(x=value, fill = name))+
  geom_density(color=NA, alpha=0.6)+
  scale_fill_manual(values=c("#219EBC","#8ECAE6"))+ 
 labs(x="Phenotyping date standard deviation in jump angle  (log)",y="Density")+
  theme_test()
```

**(Un)predictability**

*Individual ID*

```
IndID_JumpAngle_v_prior_log = priorposterior %>%
  filter(str_detect(variable, "IndID__sigma_JumpAngle")) %>%
  mutate(value=log(value))
  
ggplot(data=IndID_JumpAngle_v_prior_log, aes(x=value, fill = name))+
  geom_density(color=NA, alpha=0.6)+
  scale_fill_manual(values=c("#219EBC","#8ECAE6"))+ 
 labs(x="Individual ID standard deviation in jump angle (un)predictability (log)",y="Density")+
  theme_test()
```

*Phenotyping date*

```
PhenotypingDate_JumpAngle_v_prior_log = priorposterior %>%
  filter(str_detect(variable, "PhenotypingDate__sigma_JumpAngle")) %>%
  mutate(value=log(value))
  
ggplot(data=PhenotypingDate_JumpAngle_v_prior_log, aes(x=value, fill = name))+
  geom_density(color=NA, alpha=0.6)+
  scale_fill_manual(values=c("#219EBC","#8ECAE6"))+ 
  labs(x = "Phenotyping date standard deviation in jump angle (un)predictability (log)", y = "Density")+
  theme_test()
```

---

### Figures

#### Fixed Effects

##### FID

**Average behavior**

Z transform the slopes (slopes standardized for the standard
deviation of each covariate)

```
PS$b_logFID_TreatmentCZ = PS$b_logFID_TreatmentC*(sd(c(0,1)))
PS$b_logFID_SexCZ = PS$b_logFID_SexC*(sd(c(-0.5,0.5)))
PS$b_logFID_DayTimeCZ = PS$b_logFID_DayTimeC* (sd(md$DayTimeC))
PS$b_logFID_JumpOrderCZ = PS$b_logFID_JumpOrderC* (sd(c(1:10)))
PS$'b_logFID_TreatmentC:SexCZ' = PS$'b_logFID_TreatmentC:SexC' * 
                                          (sd(md$TreatmentC*md$SexC))


PS_FE_m_logFID = gather(PS,name_FE_m_logFID, b_z_m_logFID, 
                 b_logFID_TreatmentCZ,
                 b_logFID_SexCZ,
                 b_logFID_DayTimeCZ,
                 b_logFID_JumpOrderCZ,
                 'b_logFID_TreatmentC:SexCZ')

ggplot(PS_FE_m_logFID,(aes(y = name_FE_m_logFID, x = b_z_m_logFID))) +  
         stat_halfeye(slab_type="pdf") +
         stat_pointinterval(.width = c(.5, .95)) +
         geom_vline(xintercept=0,color = "red")+
   labs(x="Posterior distributions of fixed effect slopes", y=NULL,title = "Flight initiation distance",subtitle = "Average behavior") + 
  scale_y_discrete(labels=c("Daytime", "Jump order", "Sex","Treatment:Sex", "Treatment"))+
         theme_tidybayes()
```

**(Un)predictability**

Z transform the slopes (slopes standardized for the standard
deviation of each covariate)

```
PS$b_sigma_logFID_TreatmentCZ = PS$b_sigma_logFID_TreatmentC*(sd(c(0,1)))
PS$b_sigma_logFID_SexCZ = PS$b_sigma_logFID_SexC*(sd(c(-0.5,0.5)))
PS$b_sigma_logFID_DayTimeCZ = PS$b_sigma_logFID_DayTimeC* (sd(md$DayTimeC))
PS$b_sigma_logFID_JumpOrderCZ = PS$b_sigma_logFID_JumpOrderC* (sd(c(1:10)))
PS$'b_sigma_logFID_TreatmentC:SexCZ' = PS$'b_sigma_logFID_TreatmentC:SexC' * (sd(md$TreatmentC*md$SexC))


PS_v_logFID = gather(PS,name_FE_v_logFID, b_z_v_logFID, 
                 b_sigma_logFID_TreatmentCZ,
                 b_sigma_logFID_SexCZ,
                 b_sigma_logFID_DayTimeCZ,
                 b_sigma_logFID_JumpOrderCZ,
                 'b_sigma_logFID_TreatmentC:SexCZ')

ggplot(PS_v_logFID,(aes(y = name_FE_v_logFID, x = b_z_v_logFID))) +  
         stat_halfeye(slab_type="pdf") +
         stat_pointinterval(.width = c(.5, .95)) +
         geom_vline(xintercept=0,color = "red")+
    scale_y_discrete(labels=c("Daytime", "Jump order", "Sex","Treatment:Sex", "Treatment"))+
     labs(x="Posterior distributions of fixed effect slopes", y=NULL,title = "Flight initiation distance",subtitle = "Unpredictability") + 
         theme_tidybayes()
```

---

##### JumpDistance

**Average behavior**

Z transform the slopes (slopes standardized for the standard
deviation of each covariate)

```
PS$b_JumpDistance_TreatmentCZ = PS$b_JumpDistance_TreatmentC*(sd(c(0,1)))
PS$b_JumpDistance_SexCZ = PS$b_JumpDistance_SexC*(sd(c(-0.5,0.5)))
PS$b_JumpDistance_DayTimeCZ = PS$b_JumpDistance_DayTimeC* (sd(md$DayTimeC))
PS$b_JumpDistance_JumpOrderCZ = PS$b_JumpDistance_JumpOrderC* (sd(c(1:10)))
PS$'b_JumpDistance_TreatmentC:SexCZ' = PS$'b_JumpDistance_TreatmentC:SexC' * 
                                          (sd(md$TreatmentC*md$SexC))


PS_FE_m_JumpDistance = gather(PS,name_FE_m_JumpDistance, b_z_m_JumpDistance, 
                 b_JumpDistance_TreatmentCZ,
                 b_JumpDistance_SexCZ,
                 b_JumpDistance_DayTimeCZ,
                 b_JumpDistance_JumpOrderCZ,
                 'b_JumpDistance_TreatmentC:SexCZ')

ggplot(PS_FE_m_JumpDistance,aes(y = name_FE_m_JumpDistance, x = b_z_m_JumpDistance)) +  stat_halfeye(slab_type="pdf") +
    stat_pointinterval(.width = c(.5, .95)) +
   geom_vline(xintercept=0,color = "red")+
    scale_y_discrete(labels=c("Daytime", "Jump order", "Sex","Treatment:Sex", "Treatment"))+
     labs(x="Posterior distributions of fixed effect slopes", y=NULL,title = "Jump distance",subtitle = "Average behavior") + 
     theme_tidybayes()
```

**(Un)predictability**

Z transform the slopes (slopes standardized for the standard
deviation of each covariate)

```
PS$b_sigma_JumpDistance_TreatmentCZ = PS$b_sigma_JumpDistance_TreatmentC*(sd(c(0,1)))
PS$b_sigma_JumpDistance_SexCZ = PS$b_sigma_JumpDistance_SexC*(sd(c(-0.5,0..5)))
PS$b_sigma_JumpDistance_DayTimeCZ = PS$b_sigma_JumpDistance_DayTimeC* (sd(md$DayTimeC))
PS$b_sigma_JumpDistance_JumpOrderCZ = PS$b_sigma_JumpDistance_JumpOrderC* (sd(c(1:10)))
PS$'b_sigma_JumpDistance_TreatmentC:SexCZ' = PS$'b_sigma_JumpDistance_TreatmentC:SexC' * (sd(md$TreatmentC*md$SexC))


PS_v_JumpDistance = gather(PS,name_FE_v_JumpDistance, b_z_v_JumpDistance, 
                 b_sigma_JumpDistance_TreatmentCZ,
                 b_sigma_JumpDistance_SexCZ,
                 b_sigma_JumpDistance_DayTimeCZ,
                 b_sigma_JumpDistance_JumpOrderCZ,
                 'b_sigma_JumpDistance_TreatmentC:SexCZ')

ggplot(PS_v_JumpDistance,(aes(y = name_FE_v_JumpDistance, x = b_z_v_JumpDistance))) +  
         stat_halfeye(slab_type="pdf") +
         stat_pointinterval(.width = c(.5, .95)) +
         geom_vline(xintercept=0,color = "red")+
    scale_y_discrete(labels=c("Daytime", "Jump order", "Sex","Treatment:Sex", "Treatment"))+
       labs(x="Posterior distributions of fixed effect slopes", y=NULL,title = "Jump distance",subtitle = "Unpredictability") + 

         theme_tidybayes()
```

---

##### Jump Angle

**Average behavior**

Z transform the slopes (slopes standardized for the standard
deviation of each covariate)

```
PS$b_JumpAngle_TreatmentCZ = PS$b_JumpAngle_TreatmentC*(sd(c(0,1)))
PS$b_JumpAngle_SexCZ = PS$b_JumpAngle_SexC*(sd(c(-0.5,0.5)))
PS$b_JumpAngle_DayTimeCZ = PS$b_JumpAngle_DayTimeC* (sd(md$DayTimeC))
PS$b_JumpAngle_JumpOrderCZ = PS$b_JumpAngle_JumpOrderC* (sd(c(1:10)))
PS$'b_JumpAngle_TreatmentC:SexCZ' = PS$'b_JumpAngle_TreatmentC:SexC' * 
                                          (sd(md$TreatmentC*md$SexC))


PS_FE_m_JumpAngle = gather(PS,name_FE_m_JumpAngle, b_z_m_JumpAngle, 
                 b_JumpAngle_TreatmentCZ,
                 b_JumpAngle_SexCZ,
                 b_JumpAngle_DayTimeCZ,
                 b_JumpAngle_JumpOrderCZ,
                 'b_JumpAngle_TreatmentC:SexCZ')

ggplot(PS_FE_m_JumpAngle,aes(y = name_FE_m_JumpAngle, x = b_z_m_JumpAngle)) +  stat_halfeye(slab_type="pdf") +
    stat_pointinterval(.width = c(.5, .95)) +
   geom_vline(xintercept=0,color = "red")+
    scale_y_discrete(labels=c("Daytime", "Jump order", "Sex","Treatment:Sex", "Treatment"))+
       labs(x="Posterior distributions of fixed effect slopes", y=NULL,title = "Jump Angle",subtitle = "Average behavior") + 

     theme_tidybayes()
```

**(Un)predictability**

Z transform the slopes (slopes standardized for the standard
deviation of each covariate)

```
PS$b_sigma_JumpAngle_TreatmentCZ = PS$b_sigma_JumpAngle_TreatmentC*(sd(c(0,1)))
PS$b_sigma_JumpAngle_SexCZ = PS$b_sigma_JumpAngle_SexC*(sd(c(-0.5,0.5)))
PS$b_sigma_JumpAngle_DayTimeCZ = PS$b_sigma_JumpAngle_DayTimeC* (sd(md$DayTimeC))
PS$b_sigma_JumpAngle_JumpOrderCZ = PS$b_sigma_JumpAngle_JumpOrderC* (sd(c(1:10)))
PS$'b_sigma_JumpAngle_TreatmentC:SexCZ' = PS$'b_sigma_JumpAngle_TreatmentC:SexC' * (sd(md$TreatmentC*md$SexC))


PS_v_JumpAngle = gather(PS,name_FE_v_JumpAngle, b_z_v_JumpAngle, 
                 b_sigma_JumpAngle_TreatmentCZ,
                 b_sigma_JumpAngle_SexCZ,
                 b_sigma_JumpAngle_DayTimeCZ,
                 b_sigma_JumpAngle_JumpOrderCZ,
                 'b_sigma_JumpAngle_TreatmentC:SexCZ')

ggplot(PS_v_JumpAngle,(aes(y = name_FE_v_JumpAngle, x = b_z_v_JumpAngle))) +  
         stat_halfeye(slab_type="pdf") +
         stat_pointinterval(.width = c(.5, .95)) +
         geom_vline(xintercept=0,color = "red")+
    scale_y_discrete(labels=c("Daytime", "Jump order", "Sex","Treatment:Sex", "Treatment"))+
        labs(x="Posterior distributions of fixed effect slopes", y=NULL,title = "Jump Angle",subtitle = "Unpredictability") + 
         theme_tidybayes()
```

---

#### Correlations

**Average correlation among average traits**

```
g1 = select(PS,
cor_IndID__logFID_Intercept__JumpDistance_Intercept, cor_IndID__logFID_Intercept__JumpAngle_Intercept,
cor_IndID__JumpDistance_Intercept__JumpAngle_Intercept) %>%
  pivot_longer(col= 1:3, names_to = "cov", values_to= "val")
mean(g1$val)
```

```
## [1] 0.1125109
```

**Average correlation among predictabilities**

```
g2 = select(PS,
cor_IndID__sigma_logFID_Intercept__sigma_JumpDistance_Intercept,
cor_IndID__sigma_logFID_Intercept__sigma_JumpAngle_Intercept,    
cor_IndID__sigma_JumpDistance_Intercept__sigma_JumpAngle_Intercept) %>%
  pivot_longer(col= 1:3, names_to = "cov", values_to= "val")
mean(g2$val)
```

```
## [1] 0.09111174
```

**Average correlation between average traits and
predictabilities within trait**

```
g3 = select(PS,
cor_IndID__logFID_Intercept__sigma_logFID_Intercept,
cor_IndID__JumpDistance_Intercept__sigma_JumpDistance_Intercept,
cor_IndID__JumpAngle_Intercept__sigma_JumpAngle_Intercept) %>%
  pivot_longer(col= 1:3, names_to = "cov", values_to= "val")
mean(g3$val)
```

```
## [1] 0.02158879
```

**Average correlation between average traits and
predictabilities among traits**

```
g4 = select(PS,
cor_IndID__sigma_logFID_Intercept__JumpDistance_Intercept,
cor_IndID__logFID_Intercept__sigma_JumpDistance_Intercept,
cor_IndID__sigma_logFID_Intercept__JumpAngle_Intercept,                     
cor_IndID__sigma_JumpDistance_Intercept__JumpAngle_Intercept,
cor_IndID__logFID_Intercept__sigma_JumpAngle_Intercept,                      
cor_IndID__JumpDistance_Intercept__sigma_JumpAngle_Intercept) %>%
  pivot_longer(col= 1:6, names_to = "cov", values_to= "val")
mean(g4$val)
```

```
## [1] -0.08883375
```

```
PS_cor = pivot_longer(PS,
                               cols = starts_with("cor"),
                               names_to = "Combination",
                               values_to = "Correlation") %>%
                               select(Combination, Correlation)

PS_cor$RE = PS_cor$Combination
PS_cor = PS_cor %>% 
    separate(RE, c( "A", "RE")) %>%
    select(-"A")
    

                 
PS_cor$Combination = factor(PS_cor$Combination, 
                                     levels = c(
"cor_IndID__logFID_Intercept__JumpDistance_Intercept",
"cor_PhenotypingDate__logFID_Intercept__JumpDistance_Intercept",
"cor_PhenotypingDate__logFID_Intercept__JumpAngle_Intercept",
"cor_PhenotypingDate__logFID_Intercept__sigma_logFID_Intercept",
"cor_PhenotypingDate__logFID_Intercept__sigma_JumpDistance_Intercept",
"cor_PhenotypingDate__logFID_Intercept__sigma_JumpAngle_Intercept",
"cor_IndID__logFID_Intercept__JumpAngle_Intercept",
"cor_IndID__JumpDistance_Intercept__JumpAngle_Intercept",
"cor_PhenotypingDate__JumpDistance_Intercept__JumpAngle_Intercept",
"cor_PhenotypingDate__sigma_logFID_Intercept__JumpDistance_Intercept",
"cor_PhenotypingDate__JumpDistance_Intercept__sigma_JumpDistance_Intercept",
"cor_PhenotypingDate__JumpDistance_Intercept__sigma_JumpAngle_Intercept",
"cor_IndID__logFID_Intercept__sigma_logFID_Intercept",
"cor_IndID__sigma_logFID_Intercept__JumpDistance_Intercept",
"cor_IndID__sigma_logFID_Intercept__JumpAngle_Intercept",
"cor_PhenotypingDate__sigma_logFID_Intercept__JumpAngle_Intercept",
"cor_PhenotypingDate__sigma_JumpDistance_Intercept__JumpAngle_Intercept",
"cor_PhenotypingDate__JumpAngle_Intercept__sigma_JumpAngle_Intercept",
"cor_IndID__logFID_Intercept__sigma_JumpDistance_Intercept",
"cor_IndID__JumpDistance_Intercept__sigma_JumpDistance_Intercept",
"cor_IndID__sigma_JumpDistance_Intercept__JumpAngle_Intercept",
"cor_IndID__sigma_logFID_Intercept__sigma_JumpDistance_Intercept",
"cor_PhenotypingDate__sigma_logFID_Intercept__sigma_JumpDistance_Intercept",
"cor_PhenotypingDate__sigma_logFID_Intercept__sigma_JumpAngle_Intercept",
"cor_IndID__logFID_Intercept__sigma_JumpAngle_Intercept",
"cor_IndID__JumpDistance_Intercept__sigma_JumpAngle_Intercept",
"cor_IndID__JumpAngle_Intercept__sigma_JumpAngle_Intercept",
"cor_IndID__sigma_logFID_Intercept__sigma_JumpAngle_Intercept",
"cor_IndID__sigma_JumpDistance_Intercept__sigma_JumpAngle_Intercept",
"cor_PhenotypingDate__sigma_JumpDistance_Intercept__sigma_JumpAngle_Intercept"),
labels = c(
"IndID m_logFID x m_JumpDistance",
"Date m_logFID x m_JumpDistance",
"Date m_logFID x m_JumpAngle",
"Date m_logFID x v_logFID",
"Date m_logFID x v_JumpDistance",
"Date m_logFID x v_JumpAngle",
"IndID m_logFID x m_JumpAngle",
"IndID m_JumpDistance x m_JumpAngle",
"Date m_JumpDistance x m_JumpAngle",
"Date v_logFID x m_JumpDistance",
"Date m_JumpDistance x v_JumpDistance",
"Date m_JumpDistance x v_JumpAngle",
"IndID m_logFID x v_logFID",
"IndID v_logFID x m_JumpDistance",
"IndID v_logFID x m_JumpAngle",
"Date v_logFID x m_JumpAngle",
"Date v_JumpDistance x m_JumpAngle",
"Date m_JumpAngle x v_JumpAngle",
"IndID m_logFID x v_JumpDistance",
"IndID m_JumpDistance x v_JumpDistance",
"IndID v_JumpDistance x m_JumpAngle",
"IndID v_logFID x v_JumpDistance",
"Date v_logFID x v_JumpDistance",
"Date v_logFID x v_JumpAngle",
"IndID m_logFID x v_JumpAngle",
"IndID m_JumpDistance x v_JumpAngle",
"IndID m_JumpAngle x v_JumpAngle",
"IndID v_logFID x v_JumpAngle",
"IndID v_JumpDistance x v_JumpAngle",
"Date v_JumpDistance x v_JumpAngle"))
  

ggplot(PS_cor,(aes(y = Combination, x = Correlation, fill=RE))) +  
         stat_halfeye(slab_type="pdf", alpha=0.6) +
         stat_pointinterval(.width = c(.5, .95)) +
         geom_vline(xintercept=0,color = "red")+
        facet_wrap(~Combination, nrow =  5, ncol = 6,  scales = "free_y")+
        scale_fill_manual(values=c("#023047","#FFB703"))+
          theme_tidybayes()  +
          theme(axis.text.y = element_blank(),
                axis.title.y = element_blank(),
                axis.ticks.y = element_blank(),
                strip.text.x = element_text(size=6),
                legend.position = "bottom")
```

---

#### Random Effects

##### FID

**Average behavior**

```
PS_RE_m_logFID = data.frame(
                    group = c("Individual ID", "Date", "Residual"),
                    value = c(
                      round(mean(PS$Rp_IndID_m_logFID)*100,1),
                      round(mean(PS$Rp_PhenotypingDate_m_logFID)*100,1),
                      round(mean(PS$Rp_Res_m_logFID)*100,1))
                          ) 

ggplot(PS_RE_m_logFID, aes(x= "",y=value, fill=group))+
  geom_bar(width = 1, stat = "identity", color="white",size = 1)+
  coord_polar("y", start=0)+
  scale_fill_manual(values=c("#FFB703", "#023047", "#E4E4E4"))+
   geom_text(aes(label = value),
            position = position_stack(vjust = 0.5))+
  theme_test()+
  theme(axis.title = element_blank(),
        axis.text = element_blank(),
        axis.ticks = element_blank(),
        panel.border = element_blank())
```

**(Un)predictability**

```
PS_RE_v_logFID = data.frame(
                    group = c("Individual ID", "Date", "Residual"),
                    value = c(
                      round(mean(PS$Rp_IndID_v_logFID)*100,1),
                      round(mean(PS$Rp_PhenotypingDate_v_logFID)*100,1),
                      round(mean(PS$Rp_Res_v_logFID)*100,1))
                          ) 

ggplot(PS_RE_v_logFID, aes(x= "",y=value, fill=group))+
  geom_bar(width = 1, stat = "identity", color="white",size = 1)+
  coord_polar("y", start=0)+
  scale_fill_manual(values=c("#FFB703", "#023047", "#E4E4E4"))+
   geom_text(aes(label = value),
            position = position_stack(vjust = 0.5))+ 
  theme_test()+
  theme(axis.title = element_blank(),
        axis.text = element_blank(),
        axis.ticks = element_blank(),
        panel.border = element_blank()
        )
```

---

##### Jump Distance

**Average behavior**

```
PS_RE_m_JumpDistance = data.frame(
                    group = c("Individual ID", "Date", "Residual"),
                    value = c(
                      round(mean(PS$Rp_IndID_m_JumpDistance)*100,1),
                      round(mean(PS$Rp_PhenotypingDate_m_JumpDistance)*100,1),
                      round(mean(PS$Rp_Res_m_JumpDistance)*100,1))
                          ) 

ggplot(PS_RE_m_JumpDistance, aes(x= "",y=value, fill=group))+
  geom_bar(width = 1, stat = "identity", color="white",size = 1)+
  coord_polar("y", start=0)+
  scale_fill_manual(values=c("#FFB703", "#023047", "#E4E4E4"))+
   geom_text(aes(label = value),
            position = position_stack(vjust = 0.5))+ 
  theme_test()+
  theme(axis.title = element_blank(),
        axis.text = element_blank(),
        axis.ticks = element_blank(),
        panel.border = element_blank()
        )
```

**(Un)predictability**

```
PS_RE_v_JumpDistance = data.frame(
                    group = c("Individual ID", "Date", "Residual"),
                    value = c(
                      round(mean(PS$Rp_IndID_v_JumpDistance)*100,1),
                      round(mean(PS$Rp_PhenotypingDate_v_JumpDistance)*100,1),
                      round(mean(PS$Rp_Res_v_JumpDistance)*100,1))
                          ) 

ggplot(PS_RE_v_JumpDistance, aes(x= "",y=value, fill=group))+
  geom_bar(width = 1, stat = "identity", color="white",size = 1)+
  coord_polar("y", start=0)+
  scale_fill_manual(values=c("#FFB703", "#023047", "#E4E4E4"))+
   geom_text(aes(label = value),
            position = position_stack(vjust = 0.5))+ 
  theme_test()+
  theme(axis.title = element_blank(),
        axis.text = element_blank(),
        axis.ticks = element_blank(),
        panel.border = element_blank()
        )
```

---

##### Jump Angle

**Average behavior**

```
PS_RE_m_JumpAngle = data.frame(
                    group = c("Individual ID", "Date", "Residual"),
                    value = c(
                      round(mean(PS$Rp_IndID_m_JumpAngle)*100,1),
                      round(mean(PS$Rp_PhenotypingDate_m_JumpAngle)*100,1),
                      round(mean(PS$Rp_Res_m_JumpAngle)*100,1))
                          ) 

ggplot(PS_RE_m_JumpAngle, aes(x= "",y=value, fill=group))+
  geom_bar(width = 1, stat = "identity", color="white",size = 1)+
  coord_polar("y", start=0)+
  scale_fill_manual(values=c("#FFB703", "#023047", "#E4E4E4"))+
   geom_text(aes(label = value),
            position = position_stack(vjust = 0.5))+ 
  theme_test()+
  theme(axis.title = element_blank(),
        axis.text = element_blank(),
        axis.ticks = element_blank(),
        panel.border = element_blank()
        )
```

**(Un)predictability**

```
PS_RE_v_JumpAngle = data.frame(
                    group = c("Individual ID", "Date", "Residual"),
                    value = c(
                      round(mean(PS$Rp_IndID_v_JumpAngle)*100,1),
                      round(mean(PS$Rp_PhenotypingDate_v_JumpAngle)*100,1),
                      round(mean(PS$Rp_Res_v_JumpAngle)*100,1))
                          ) 

ggplot(PS_RE_v_JumpAngle, aes(x= "",y=value, fill=group))+
  geom_bar(width = 1, stat = "identity", color="white",size = 1)+
  coord_polar("y", start=0)+
  scale_fill_manual(values=c("#FFB703", "#023047", "#E4E4E4"))+
   geom_text(aes(label = value),
            position = position_stack(vjust = 0.5))+ 
  theme_test()+
  theme(axis.title = element_blank(),
        axis.text = element_blank(),
        axis.ticks = element_blank(),
        panel.border = element_blank()
        )
```

---

#### Repeatabilities

##### IndID

**Average behavior**

```
Rp_IndID_m = PS %>%
  select(starts_with("Rp"))%>%
  gather("name", "value") %>%
  filter(str_detect(name, "IndID")) %>%
  filter(str_detect(name, "_m_"))

ggplot(Rp_IndID_m,aes(y = name, x = value)) +  stat_halfeye(slab_type="pdf") +
    stat_pointinterval(.width = c(.5, .95)) +
    labs(x="Individual ID repeatability (average behavior)", y= "Trait")+
  scale_y_discrete(labels = c("Jump angle", "Jump distance", "FID"))+
   theme_tidybayes()
```

*logFID x Jump Distance*

```
Rp_IndID_m_logFID_JumpDistance = Rp_IndID_m %>%
  filter(str_detect(name, "logFID")|str_detect(name, "JumpDistance"))

print(t.test(value~name, data=Rp_IndID_m_logFID_JumpDistance))
```

```
## 
##  Welch Two Sample t-test
## 
## data:  value by name
## t = 164.9, df = 2983.5, p-value < 0.00000000000000022
## alternative hypothesis: true difference in means between group Rp_IndID_m_JumpDistance and group Rp_IndID_m_logFID is not equal to 0
## 95 percent confidence interval:
##  0.2010379 0.2058765
## sample estimates:
## mean in group Rp_IndID_m_JumpDistance       mean in group Rp_IndID_m_logFID 
##                            0.27658623                            0.07312904
```

*logFID x Jump Angle*

```
Rp_IndID_m_logFID_JumpAngle = Rp_IndID_m %>%
  filter(str_detect(name, "logFID")|str_detect(name, "JumpAngle"))

print(t.test(value~name, data=Rp_IndID_m_logFID_JumpAngle))
```

```
## 
##  Welch Two Sample t-test
## 
## data:  value by name
## t = 40.419, df = 3834.3, p-value < 0.00000000000000022
## alternative hypothesis: true difference in means between group Rp_IndID_m_JumpAngle and group Rp_IndID_m_logFID is not equal to 0
## 95 percent confidence interval:
##  0.03439666 0.03790369
## sample estimates:
## mean in group Rp_IndID_m_JumpAngle    mean in group Rp_IndID_m_logFID 
##                         0.10927921                         0.07312904
```

*Jump Distance x Jump Angle*

```
Rp_IndID_m_JumpDistance_JumpAngle = Rp_IndID_m %>%
  filter(str_detect(name, "JumpDistance")|str_detect(name, "JumpAngle"))

print(t.test(value~name, data=Rp_IndID_m_JumpDistance_JumpAngle))
```

```
## 
##  Welch Two Sample t-test
## 
## data:  value by name
## t = -128.79, df = 3378.8, p-value < 0.00000000000000022
## alternative hypothesis: true difference in means between group Rp_IndID_m_JumpAngle and group Rp_IndID_m_JumpDistance is not equal to 0
## 95 percent confidence interval:
##  -0.1698541 -0.1647599
## sample estimates:
##    mean in group Rp_IndID_m_JumpAngle mean in group Rp_IndID_m_JumpDistance 
##                             0.1092792                             0.2765862
```

**Unpredictability**

```
Rp_IndID_v = PS %>%
  select(starts_with("Rp"))%>%
  gather("name", "value") %>%
  filter(str_detect(name, "IndID")) %>%
  filter(str_detect(name, "_v_"))

ggplot(Rp_IndID_v,aes(y = name, x = value)) +  stat_halfeye(slab_type="pdf") +
    stat_pointinterval(.width = c(.5, .95)) +
    labs(x="Individual ID repeatability (unpredictability)", y= "Trait")+
  scale_y_discrete(labels = c("Jump angle", "Jump distance", "FID"))+
     theme_tidybayes()
```

*logFID x Jump Distance*

```
Rp_IndID_v_logFID_JumpDistance = Rp_IndID_v %>%
  filter(str_detect(name, "logFID")|str_detect(name, "JumpDistance"))

print(t.test(value~name, data=Rp_IndID_v_logFID_JumpDistance))
```

```
## 
##  Welch Two Sample t-test
## 
## data:  value by name
## t = -33.066, df = 3162.2, p-value < 0.00000000000000022
## alternative hypothesis: true difference in means between group Rp_IndID_v_JumpDistance and group Rp_IndID_v_logFID is not equal to 0
## 95 percent confidence interval:
##  -0.02020183 -0.01794010
## sample estimates:
## mean in group Rp_IndID_v_JumpDistance       mean in group Rp_IndID_v_logFID 
##                            0.03027787                            0.04934883
```

*logFID x JumpAngle*

```
Rp_IndID_v_logFID_JumpAngle = Rp_IndID_v %>%
  filter(str_detect(name, "logFID")|str_detect(name, "JumpAngle"))

print(t.test(value~name, data=Rp_IndID_v_logFID_JumpAngle))
```

```
## 
##  Welch Two Sample t-test
## 
## data:  value by name
## t = 35.072, df = 3976.2, p-value < 0.00000000000000022
## alternative hypothesis: true difference in means between group Rp_IndID_v_JumpAngle and group Rp_IndID_v_logFID is not equal to 0
## 95 percent confidence interval:
##  0.02267531 0.02536053
## sample estimates:
## mean in group Rp_IndID_v_JumpAngle    mean in group Rp_IndID_v_logFID 
##                         0.07336675                         0.04934883
```

*JumpDistance x JumpAngle*

```
Rp_IndID_v_JumpDistance_JumpAngle = Rp_IndID_v %>%
  filter(str_detect(name, "JumpDistance")|str_detect(name, "JumpAngle"))

print(t.test(value~name, data=Rp_IndID_v_JumpDistance_JumpAngle))
```

```
## 
##  Welch Two Sample t-test
## 
## data:  value by name
## t = 78.94, df = 3306, p-value < 0.00000000000000022
## alternative hypothesis: true difference in means between group Rp_IndID_v_JumpAngle and group Rp_IndID_v_JumpDistance is not equal to 0
## 95 percent confidence interval:
##  0.04201866 0.04415911
## sample estimates:
##    mean in group Rp_IndID_v_JumpAngle mean in group Rp_IndID_v_JumpDistance 
##                            0.07336675                            0.03027787
```

---

##### Phenotyping Date

**Average behavior**

```
Rp_PhenotypingDate_m = PS %>%
  select(starts_with("Rp"))%>%
  gather("name", "value") %>%
  filter(str_detect(name, "PhenotypingDate")) %>%
  filter(str_detect(name, "_m_"))

ggplot(Rp_PhenotypingDate_m,aes(y = name, x = value)) +  stat_halfeye(slab_type="pdf") +
    stat_pointinterval(.width = c(.5, .95)) +
      labs(x="Phenotyping date repeatability (average behavior)", y= "Trait")+
  scale_y_discrete(labels = c("Jump angle", "Jump distance", "FID"))+
   theme_tidybayes()
```

*logFID x Jump Distance*

```
Rp_PhenotypingDate_m_logFID_JumpDistance = Rp_PhenotypingDate_m %>%
  filter(str_detect(name, "logFID")|str_detect(name, "JumpDistance"))

print(t.test(value~name, data=Rp_PhenotypingDate_m_logFID_JumpDistance))
```

```
## 
##  Welch Two Sample t-test
## 
## data:  value by name
## t = -68.742, df = 3270.7, p-value < 0.00000000000000022
## alternative hypothesis: true difference in means between group Rp_PhenotypingDate_m_JumpDistance and group Rp_PhenotypingDate_m_logFID is not equal to 0
## 95 percent confidence interval:
##  -0.2398291 -0.2265274
## sample estimates:
## mean in group Rp_PhenotypingDate_m_JumpDistance 
##                                       0.1108601 
##       mean in group Rp_PhenotypingDate_m_logFID 
##                                       0.3440384
```

*logFID x Jump Angle*

```
Rp_PhenotypingDate_m_logFID_JumpAngle = Rp_PhenotypingDate_m %>%
  filter(str_detect(name, "logFID")|str_detect(name, "JumpAngle"))

print(t.test(value~name, data=Rp_PhenotypingDate_m_logFID_JumpAngle))
```

```
## 
##  Welch Two Sample t-test
## 
## data:  value by name
## t = -106.38, df = 2210.7, p-value < 0.00000000000000022
## alternative hypothesis: true difference in means between group Rp_PhenotypingDate_m_JumpAngle and group Rp_PhenotypingDate_m_logFID is not equal to 0
## 95 percent confidence interval:
##  -0.3234948 -0.3117838
## sample estimates:
## mean in group Rp_PhenotypingDate_m_JumpAngle 
##                                   0.02639906 
##    mean in group Rp_PhenotypingDate_m_logFID 
##                                   0.34403837
```

*Jump Distance x Jump Angle*

```
Rp_PhenotypingDate_m_JumpDistance_JumpAngle = Rp_PhenotypingDate_m %>%
  filter(str_detect(name, "JumpDistance")|str_detect(name, "JumpAngle"))

print(t.test(value~name, data=Rp_PhenotypingDate_m_JumpDistance_JumpAngle))
```

```
## 
##  Welch Two Sample t-test
## 
## data:  value by name
## t = -45.213, df = 2577.4, p-value < 0.00000000000000022
## alternative hypothesis: true difference in means between group Rp_PhenotypingDate_m_JumpAngle and group Rp_PhenotypingDate_m_JumpDistance is not equal to 0
## 95 percent confidence interval:
##  -0.08812410 -0.08079799
## sample estimates:
##    mean in group Rp_PhenotypingDate_m_JumpAngle 
##                                      0.02639906 
## mean in group Rp_PhenotypingDate_m_JumpDistance 
##                                      0.11086010
```

**Unpredictability**

```
Rp_PhenotypingDate_v = PS %>%
  select(starts_with("Rp"))%>%
  gather("name", "value") %>%
  filter(str_detect(name, "PhenotypingDate")) %>%
  filter(str_detect(name, "_v_"))

ggplot(Rp_PhenotypingDate_v,aes(y = name, x = value)) +  stat_halfeye(slab_type="pdf") +
    stat_pointinterval(.width = c(.5, .95)) +
        labs(x="Phenotyping date repeatability (unpredictability)", y= "Trait")+
  scale_y_discrete(labels = c("Jump angle", "Jump distance", "FID"))+
   theme_tidybayes()
```

*logFID x Jump Distance*

```
Rp_PhenotypingDate_v_logFID_JumpDistance = Rp_PhenotypingDate_v %>%
  filter(str_detect(name, "logFID")|str_detect(name, "JumpDistance"))

print(t.test(value~name, data=Rp_PhenotypingDate_v_logFID_JumpDistance))
```

```
## 
##  Welch Two Sample t-test
## 
## data:  value by name
## t = -12.968, df = 3251.9, p-value < 0.00000000000000022
## alternative hypothesis: true difference in means between group Rp_PhenotypingDate_v_JumpDistance and group Rp_PhenotypingDate_v_logFID is not equal to 0
## 95 percent confidence interval:
##  -0.008680173 -0.006400140
## sample estimates:
## mean in group Rp_PhenotypingDate_v_JumpDistance 
##                                     0.008950222 
##       mean in group Rp_PhenotypingDate_v_logFID 
##                                     0.016490379
```

*logFID x Jump Angle*

```
Rp_PhenotypingDate_v_logFID_JumpAngle = Rp_PhenotypingDate_v %>%
  filter(str_detect(name, "logFID")|str_detect(name, "JumpAngle"))

print(t.test(value~name, data=Rp_PhenotypingDate_v_logFID_JumpAngle))
```

```
## 
##  Welch Two Sample t-test
## 
## data:  value by name
## t = 8.2976, df = 3841, p-value < 0.00000000000000022
## alternative hypothesis: true difference in means between group Rp_PhenotypingDate_v_JumpAngle and group Rp_PhenotypingDate_v_logFID is not equal to 0
## 95 percent confidence interval:
##  0.005016757 0.008120995
## sample estimates:
## mean in group Rp_PhenotypingDate_v_JumpAngle 
##                                   0.02305926 
##    mean in group Rp_PhenotypingDate_v_logFID 
##                                   0.01649038
```

*Jump Distance x Jump Angle*

```
Rp_PhenotypingDate_v_JumpDistance_JumpAngle = Rp_PhenotypingDate_v %>%
  filter(str_detect(name, "JumpDistance")|str_detect(name, "JumpAngle"))

print(t.test(value~name, data=Rp_PhenotypingDate_v_JumpDistance_JumpAngle))
```

```
## 
##  Welch Two Sample t-test
## 
## data:  value by name
## t = 20.695, df = 2885.2, p-value < 0.00000000000000022
## alternative hypothesis: true difference in means between group Rp_PhenotypingDate_v_JumpAngle and group Rp_PhenotypingDate_v_JumpDistance is not equal to 0
## 95 percent confidence interval:
##  0.01277225 0.01544582
## sample estimates:
##    mean in group Rp_PhenotypingDate_v_JumpAngle 
##                                     0.023059255 
## mean in group Rp_PhenotypingDate_v_JumpDistance 
##                                     0.008950222
```

---

##### Residual

**Average behavior**

```
Rp_Res_m = PS %>%
  select(starts_with("Rp"))%>%
  gather("name", "value") %>%
  filter(str_detect(name, "Res")) %>%
  filter(str_detect(name, "_m_"))

ggplot(Rp_Res_m,aes(y = name, x = value)) +  stat_halfeye(slab_type="pdf") +
    stat_pointinterval(.width = c(.5, .95)) +
      labs(x="Residual repeatability (average behavior)", y= "Trait")+
  scale_y_discrete(labels = c("Jump angle", "Jump distance", "FID"))+
   theme_tidybayes()
```

*logFID x Jump Distance*

```
Rp_Res_m_logFID_JumpDistance = Rp_Res_m %>%
  filter(str_detect(name, "logFID")|str_detect(name, "JumpDistance"))

print(t.test(value~name, data=Rp_Res_m_logFID_JumpDistance))
```

```
## 
##  Welch Two Sample t-test
## 
## data:  value by name
## t = 9.8517, df = 3099.5, p-value < 0.00000000000000022
## alternative hypothesis: true difference in means between group Rp_Res_m_JumpDistance and group Rp_Res_m_logFID is not equal to 0
## 95 percent confidence interval:
##  0.02380588 0.03563629
## sample estimates:
## mean in group Rp_Res_m_JumpDistance       mean in group Rp_Res_m_logFID 
##                           0.6125537                           0.5828326
```

*logFID x Jump Angle*

```
Rp_Res_m_logFID_JumpAngle = Rp_Res_m %>%
  filter(str_detect(name, "logFID")|str_detect(name, "JumpAngle"))

print(t.test(value~name, data=Rp_Res_m_logFID_JumpAngle))
```

```
## 
##  Welch Two Sample t-test
## 
## data:  value by name
## t = 100.76, df = 2452.3, p-value < 0.00000000000000022
## alternative hypothesis: true difference in means between group Rp_Res_m_JumpAngle and group Rp_Res_m_logFID is not equal to 0
## 95 percent confidence interval:
##  0.2760107 0.2869675
## sample estimates:
## mean in group Rp_Res_m_JumpAngle    mean in group Rp_Res_m_logFID 
##                        0.8643217                        0.5828326
```

*Jump Distance x Jump Angle*

```
Rp_Res_m_JumpDistance_JumpAngle = Rp_Res_m %>%
  filter(str_detect(name, "JumpDistance")|str_detect(name, "JumpAngle"))

print(t.test(value~name, data=Rp_Res_m_JumpDistance_JumpAngle))
```

```
## 
##  Welch Two Sample t-test
## 
## data:  value by name
## t = 147.72, df = 3334.1, p-value < 0.00000000000000022
## alternative hypothesis: true difference in means between group Rp_Res_m_JumpAngle and group Rp_Res_m_JumpDistance is not equal to 0
## 95 percent confidence interval:
##  0.2484264 0.2551097
## sample estimates:
##    mean in group Rp_Res_m_JumpAngle mean in group Rp_Res_m_JumpDistance 
##                           0.8643217                           0.6125537
```

**Unpredictability**

```
Rp_Res_v = PS %>%
  select(starts_with("Rp"))%>%
  gather("name", "value") %>%
  filter(str_detect(name, "Res")) %>%
  filter(str_detect(name, "_v_"))

ggplot(Rp_Res_v,aes(y = name, x = value)) +  stat_halfeye(slab_type="pdf") +
    stat_pointinterval(.width = c(.5, .95)) +
     labs(x="Residual repeatability (unpredictability)", y= "Trait")+
  scale_y_discrete(labels = c("Jump angle", "Jump distance", "FID"))+
   theme_tidybayes()
```

*logFID x Jump Distance*

```
Rp_Res_v_logFID_JumpDistance = Rp_Res_v %>%
  filter(str_detect(name, "logFID")|str_detect(name, "JumpDistance"))

print(t.test(value~name, data=Rp_Res_v_logFID_JumpDistance))
```

```
## 
##  Welch Two Sample t-test
## 
## data:  value by name
## t = 31.828, df = 3248.6, p-value < 0.00000000000000022
## alternative hypothesis: true difference in means between group Rp_Res_v_JumpDistance and group Rp_Res_v_logFID is not equal to 0
## 95 percent confidence interval:
##  0.02497181 0.02825043
## sample estimates:
## mean in group Rp_Res_v_JumpDistance       mean in group Rp_Res_v_logFID 
##                           0.9607719                           0.9341608
```

*logFID x Jump Angle*

```
Rp_Res_v_logFID_JumpAngle = Rp_Res_v %>%
  filter(str_detect(name, "logFID")|str_detect(name, "JumpAngle"))

print(t.test(value~name, data=Rp_Res_v_logFID_JumpAngle))
```

```
## 
##  Welch Two Sample t-test
## 
## data:  value by name
## t = -31.623, df = 3953.5, p-value < 0.00000000000000022
## alternative hypothesis: true difference in means between group Rp_Res_v_JumpAngle and group Rp_Res_v_logFID is not equal to 0
## 95 percent confidence interval:
##  -0.03248312 -0.02869047
## sample estimates:
## mean in group Rp_Res_v_JumpAngle    mean in group Rp_Res_v_logFID 
##                        0.9035740                        0.9341608
```

*Jump Distance x Jump Angle*

```
Rp_Res_v_JumpDistance_JumpAngle = Rp_Res_v %>%
  filter(str_detect(name, "JumpDistance")|str_detect(name, "JumpAngle"))

print(t.test(value~name, data=Rp_Res_v_JumpDistance_JumpAngle))
```

```
## 
##  Welch Two Sample t-test
## 
## data:  value by name
## t = -73.855, df = 3460, p-value < 0.00000000000000022
## alternative hypothesis: true difference in means between group Rp_Res_v_JumpAngle and group Rp_Res_v_JumpDistance is not equal to 0
## 95 percent confidence interval:
##  -0.05871636 -0.05567947
## sample estimates:
##    mean in group Rp_Res_v_JumpAngle mean in group Rp_Res_v_JumpDistance 
##                           0.9035740                           0.9607719
```

---

## References

O’Dea, R. E., Noble, D. W. A., & Nakagawa, S. (2022). Unifying
individual differences in personality, predictability and plasticity: A
practical guide. Methods in Ecology and Evolution, 13, 278– 293. https://doi..org/10.1111/2041-210X.13755
